# Massive lateral transfer of genes encoding plant cell wall-degrading enzymes to the mycoparasitic fungus *Trichoderma* from its plant-associated hosts

**DOI:** 10.1371/journal.pgen.1007322

**Published:** 2018-04-09

**Authors:** Irina S. Druzhinina, Komal Chenthamara, Jian Zhang, Lea Atanasova, Dongqing Yang, Youzhi Miao, Mohammad J. Rahimi, Marica Grujic, Feng Cai, Shadi Pourmehdi, Kamariah Abu Salim, Carina Pretzer, Alexey G. Kopchinskiy, Bernard Henrissat, Alan Kuo, Hope Hundley, Mei Wang, Andrea Aerts, Asaf Salamov, Anna Lipzen, Kurt LaButti, Kerrie Barry, Igor V. Grigoriev, Qirong Shen, Christian P. Kubicek

**Affiliations:** 1 Microbiology and Applied Genomics Group, Research Area Biochemical Technology, Institute of Chemical, Environmental & Bioscience Engineering, TU Wien, Vienna, Austria; 2 Jiangsu Provincial Key Lab of Organic Solid Waste Utilization, Nanjing Agricultural University, Nanjing, China; 3 Environmental and Life Sciences, Universiti Brunei Darussalam, Bandar Seri Begawan, Brunei Darussalam; 4 Architecture et Fonction des Macromolécules Biologiques, CNRS, Aix-Marseille Université, Marseille, France; 5 INRA, USC 1408 AFMB, Marseille, France; 6 Department of Biological Sciences, King Abdulaziz University, Jeddah, Saudi Arabia; 7 US Department of Energy Joint Genome Institute, Walnut Creek, CA, United States of America; 8 Department of Plant and Microbial Biology, University of California Berkeley, Berkeley, CA, United States of America; FRANCE

## Abstract

Unlike most other fungi, molds of the genus *Trichoderma* (Hypocreales, Ascomycota) are aggressive parasites of other fungi and efficient decomposers of plant biomass. Although nutritional shifts are common among hypocrealean fungi, there are no examples of such broad substrate versatility as that observed in *Trichoderma*. A phylogenomic analysis of 23 hypocrealean fungi (including nine *Trichoderma* spp. and the related *Escovopsis weberi*) revealed that the genus *Trichoderma* has evolved from an ancestor with limited cellulolytic capability that fed on either fungi or arthropods. The evolutionary analysis of *Trichoderma* genes encoding plant cell wall-degrading carbohydrate-active enzymes and auxiliary proteins (pcwdCAZome, 122 gene families) based on a gene tree / species tree reconciliation demonstrated that the formation of the genus was accompanied by an unprecedented extent of lateral gene transfer (LGT). Nearly one-half of the genes in *Trichoderma* pcwdCAZome (41%) were obtained via LGT from plant-associated filamentous fungi belonging to different classes of Ascomycota, while no LGT was observed from other potential donors. In addition to the ability to feed on unrelated fungi (such as Basidiomycota), we also showed that *Trichoderma* is capable of endoparasitism on a broad range of Ascomycota, including extant LGT donors. This phenomenon was not observed in *E*. *weberi* and rarely in other mycoparasitic hypocrealean fungi. Thus, our study suggests that LGT is linked to the ability of *Trichoderma* to parasitize taxonomically related fungi (up to adelphoparasitism in strict sense). This may have allowed primarily mycotrophic *Trichoderma* fungi to evolve into decomposers of plant biomass.

## Author summary

Individual fungi rely on particular host organisms or substrates for their nutrition. Therefore, the genomes of fungi feeding on plant biomass necessarily contain genes encoding plant cell wall-degrading enzymes, while animal parasites may depend on proteolytic activity. Molds in the genus *Trichoderma* (Ascomycota) display a unique nutritional versatility. They can feed on other fungi, attack animals, and degrade plant debris. The later property is so efficient that one species (*T*. *reesei*) is commercially used for the production of cellulolytic enzymes required for making biofuels and other industry. In this work, we have investigated the evolution of proteins required for plant cell wall degradation in nine *Trichoderma* genomes and found an unprecedented number of lateral gene transfer (LGT) events for genes encoding these enzymes. Interestingly, the transfers specifically occurred from Ascomycota molds that feed on plants. We detected no cases of LGT from other fungi (e.g., mushrooms or wood-rotting fungi from Basidiomycota) that are frequent hosts of *Trichoderma*. Therefore, we propose that LGT may be linked to the ability of *Trichoderma* to parasitize on related organisms. This is a characteristic ecological trait that distinguishes *Trichoderma* from other mycoparasitic fungi. In this report, we demonstrate that the lateral transfer of genes may result in a profound nutritional expansion and contribute to the emergence of a generalist capable of feeding on organic matter of any origin.

## Introduction

Fungi are heterotrophs that live either inside or on the surface of their food. They feed by secreting cocktails of digestive enzymes that break down a diversity of biopolymers, such as cellulose, hemicellulose, lignin, chitin, lipids, and proteins. The resulting soluble products are subsequently absorbed into the fungal cells and metabolised. Many fungi form biotrophic interactions with other organisms (e.g. parasitism), while others decompose dead organic matter (polyphagy, see Supporting Information S1 Text for terminology) [1]. Similar to other heterotrophs, individual fungi usually rely on particular host organisms or substrates for their nutrition. This is reflected in the diverse composition of their genetically encoded digestive enzymes. Thus, fungi feeding on plant biomass (phytophags and plant parasites; Supporting Information S1 Text) use mainly lignocellulolytic enzymes [2, 3], while animal pathogens deploy proteolytic activities for this purpose [3].

Fungi of the genus *Trichoderma* (Hypocreales, Pezizomycotina, Ascomycota) display a unique nutritional versatility (Supporting Information S1 Text) as they can form biotrophic interactions with fungi (mycoparasites [4]), animals (opportunistic parasites of immunocompromised humans [5–7]), and plants (phytoparasites [8]). *Trichoderma* spp. can also feed on dead fungi (mycophagy) and efficiently degrade plant debris (phytophagy) [4]. One such species, *T*. *reesei*, is commercially used for the production of cellulolytic enzymes required to produce biofuels [9–11]. Other *Trichoderma* spp. are used to develop biofungicides, an attractive alternative and supplement to chemical pesticides [12]. Although the two nutritional strategies (feeding on plant biomass and on fungi) were initially attributed to different species, ecophysiological studies have shown that all *Trichoderma* species are efficient mycoparasites, including *T*. *reesei* [1, 4, 13–15]. Many species possess high cellulolytic activity [16–18] and/or are symptomless parasites of plants (endophytes) [19]. A brief review of the nutritional versatility of *Trichoderma* spp. is given in Supporting Information S1 Text. Genus-wide studies of the nutritional traits of *Trichoderma* have revealed that shifts from ancestral mycoparasitism to phytophagy and back again occurred several times during *Trichoderma* evolution [16]. The best available explanation for such inter-kingdom (fungi <–> plants) nutritional jumps is the host-habitat hypothesis [20], which posits that sympatric cohabitation increases the chance of host/substrate shifts. For *Trichoderma*, a widely accepted theory proposes that ancestral species could parasitize fungal hyphae growing in decaying wood and, thus have evolved the ability to degrade plant biomass [21]. However, the mechanisms underpinning this transition are not known. Interestingly, the genome analysis of another mycoparasitic hypocrealean fungus, *Escovopsis weberi*, (which feeds on cellulolytic fungal gardens of leaf-cutting ants and therefore lives in proximity to lignocellulose) did not reveal any enrichment for genes encoding cellulases and xylanases [22]. This finding challenges the host-habitat hypothesis and shows that parasitism on a lignocellulolytic host does not necessarily result in an enhancement of lignocellulolytic machinery.

To understand the evolutionary mechanisms that lead *Trichoderma* to grow on plant biomass and, thereby expand its nutritional range, we performed a phylogenetic analysis of the plant cell wall-degrading carbohydrate-active enzymes and auxiliary proteins encoded in the genomes of nine species of *Trichoderma* that are members of three major infrageneric clades [23] plus twelve other Hypocreales fungi. Our gene tree / species tree reconciliation analysis revealed massive lateral transfer of genes (LGT) encoding plant cell wall-degrading enzymes to *Trichoderma* from plant-associated Ascomycota hosts. The results suggest that LGT from other ascomycetes was likely facilitated by expansion of *Trichoderma* mycoparasitic host range to these fungi, and this genetic phenomenon has been an important event in the evolution of this trait.

## Results

### All *Trichoderma* spp. can feed on plant and fungal biomass

To assess *Trichoderma* nutritional preferences with respect to plant and fungal biomass, we compared nine species belonging to the three major infrageneric groups (*T*. *reesei*, *T*. *parareesei*, *T*. *longibrachiatum* and *T*. *citrinoviride* from section *Longibrachiatum*; *T*. *harzianum*, *T*. *guizhouense*, *T*. *virens* from section *Pachybasium;* and *T*. *atroviride* and *T*. *asperellum* from section *Trichoderma*, Supporting Information S1 Table) with mycoparasitic *E*. *weberi* (Hypocreales, Ascomycota) and the cellulolytic and endophytic *Pestalotiopsis fici* (Xylariales, Ascomycota [24]). To approximate conditions in nature, we used (i) cell walls of fungus *Ganoderma lucidum* (Polyporales, Basidiomycota) and (ii) epiphyte-free dried leaves and biologically pre-degraded wood for the species *Shorea johorensis* (Dipterocarpaceae, Plantae). *G*. *lucidum* and *S*. *johorensis* were selected as sources of biomass because of the tropical occurrence of *T*. *reesei*, *E*. *weberi*, and *P*. *fici*, while other fungi were considered cosmopolitan. Also, we tested a diversity of plant-related substrates, such as coniferous commercial wood, microcrystalline cellulose, wheat straw, and pectin (Supporting Information S1 Fig). All fungi grew well on the cell walls of *G*. *lucidum*. Aside from this substrate, *E*. *weberi* only formed a small amount of biomass on leaves. *Trichoderma* spp. and *P*. *fici* grew equally well on all substrates that consisted of plant biomass. In general, the nine *Trichoderma* spp. showed remarkable similarities in their ability to feed on plant biomass. In contrast, *E*. *weberi* did not exhibit this nutritional versatility.

### *Trichoderma* and *Escovopsis* form a monophyletic clade and share a common ancestor with entomoparasitic fungi

**Fig 1:**
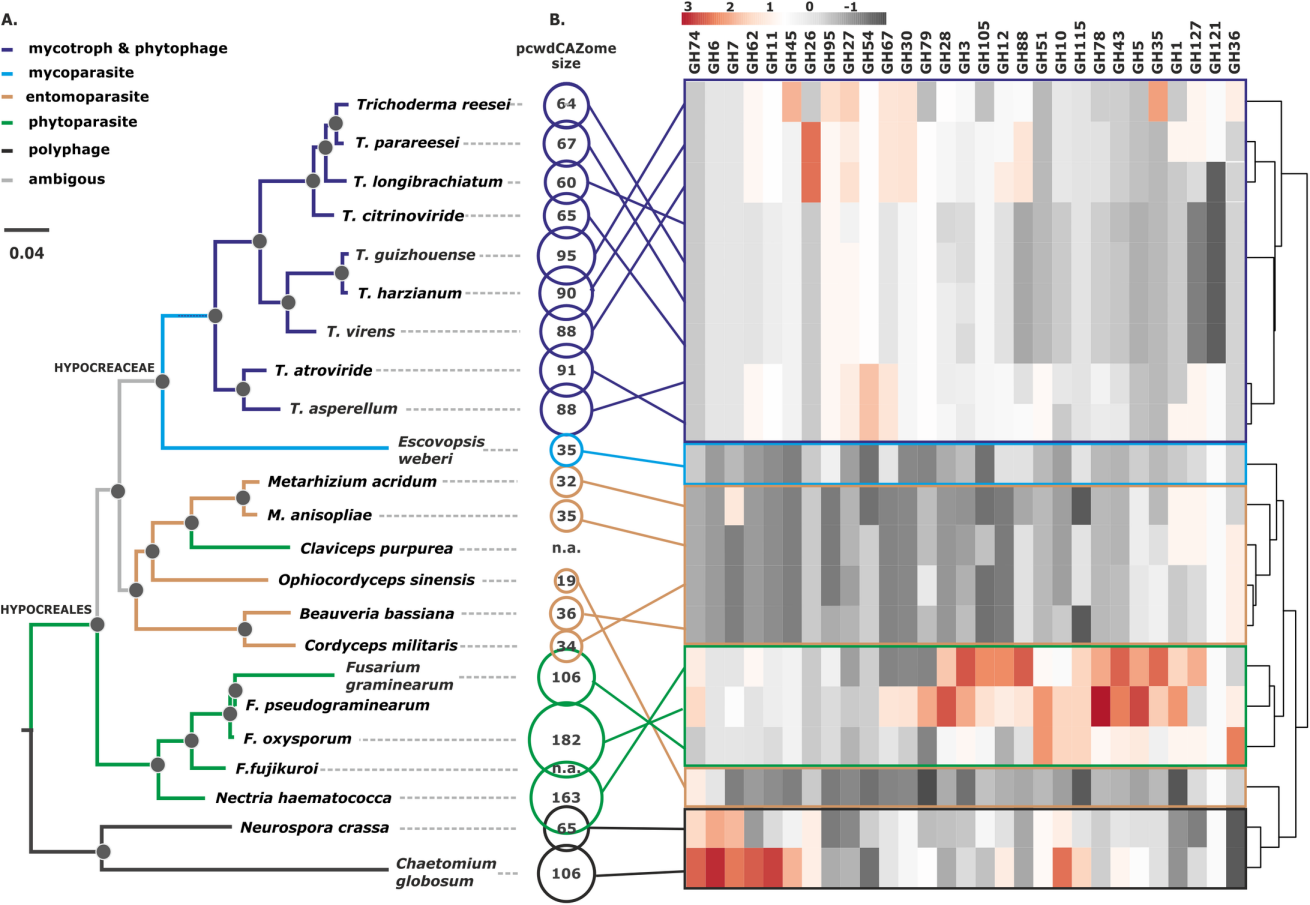
Phylogeny of Hypocreales and the composition of their pcwdCAZomes. **A**. Bayesian phylogram obtained based on the curated concatenated alignments of 100 orthologous neutrally evolving proteins of Hypocreales and two other Sordariomycetes. Black dots above nodes indicate posterior probability support > 0.95. The colors of the branches indicate the major nutritional strategy in the group (see insert) as described in Supporting Information S1 Text. **B**. The size of each pcwdCAZome per species is shown as a circle; n.a. means not available. The heat map shows the gene number for each GH family in the Hypocreales fungi examined; cluster analysis was performed with Euclidian distance and complete linkage for rows. The corresponding data matrix is presented in Supporting Information S3 Table. GH indicates glycosyl hydrolase family.

Considering the abundance of plant-associated fungi in the order Hypocreales, we hypothesized that the phytophagy of *Trichoderma* was maintained during its evolution, whereas *E*. *weberi* may have lost this ability over the course of its specialization that allowed it to parasitize on Agaricales mushrooms cultivated by ants [22]. To test this hypothesis, we reconstructed the evolutionary history of *Trichoderma*. We used 21 whole-genome sequences for fungi of the order Hypocreales, including *E*. *weberi* [22], five newly sequenced genomes of *Trichoderma* (*T*. *longibrachiatum*, *T*. *citrinoviride*, *T*. *harzianum*, *T*. *guizhouense*, and *T*. *asperellum*), and previously published genomes of *T*. *reesei* [25], *T*. *virens* [26], *T*. *atroviride* [26], and *T*. *parareesei* [27] (Supporting Information S1 Table). We selected 100 orthologous, neutrally evolving, unlinked genes encoding proteins required for a diversity of cellular functions (Supporting Information S2 Table). We reconstructed their individual phylogenies based on both nucleotide and amino acid sequences (Data deposited at http://itol.embl.de/shared/druzhininaetal). Each gene was tested for a neutral evolution using Tajima’s D test (Supporting Information S2 Table) and concatenated into an alignment of 47,726 amino acids in length (Supporting Information S2 Table). The details of the phylogenetic analyses are given in Supporting Information S2 Table. This analysis revealed that the monophyletic family Hypocreaceae, represented here by the *Escovopsis* and *Trichoderma* genera, shared a last common ancestor with fungi from the families Cordycipitaceae, Ophiocordycipitaceae, and Clavicipitaceae, which are dominated by extant entomoparasitic fungi (Fig 1A). The branch leading to the plant-associated Nectriaceae family diverged earlier in the course of the evolution of the Hypocreales.

### The *Trichoderma* pcwdCAZome is distinct from that of other hypocrealean fungi

The evolutionary history of *Trichoderma* explains its ability to efficiently derive nutrition from living and dead fungi ([4], see above) and its interactions with animals [4]. If the ability of *Trichoderma* to degrade plant biomass was inherited via vertical gene transfer, its phytophagy should resemble that of other Hypocreales fungi, especially those of the phytoparasitic family Nectriaceae. To test this, we identified all genes of the nine *Trichoderma* species that encode carbohydrate-active enzymes (CAZome, as defined at http://www.cazy.org/Genomes.html) in *Trichoderma* and selected those that are known to be involved in the plant cell wall degradation (pcwdCAZome). We retrieved proteins from all glycoside hydrolase (GH) families, which are active in hydrolysis of cellulose, the xylan backbone, other hemicelluloses and hemicellulose side chains, and pectin and its side chains. This search resulted in a total of 32 GH families. The PL1 family of pectate lyases and two accessory protein families (the AA9 lytic polysaccharide monooxygenases and the expansin-like protein swollenin) were included comprising a total of 746 proteins from the nine *Trichoderma* spp. genomes (Supporting Information S3 Table).

**Fig 2:**
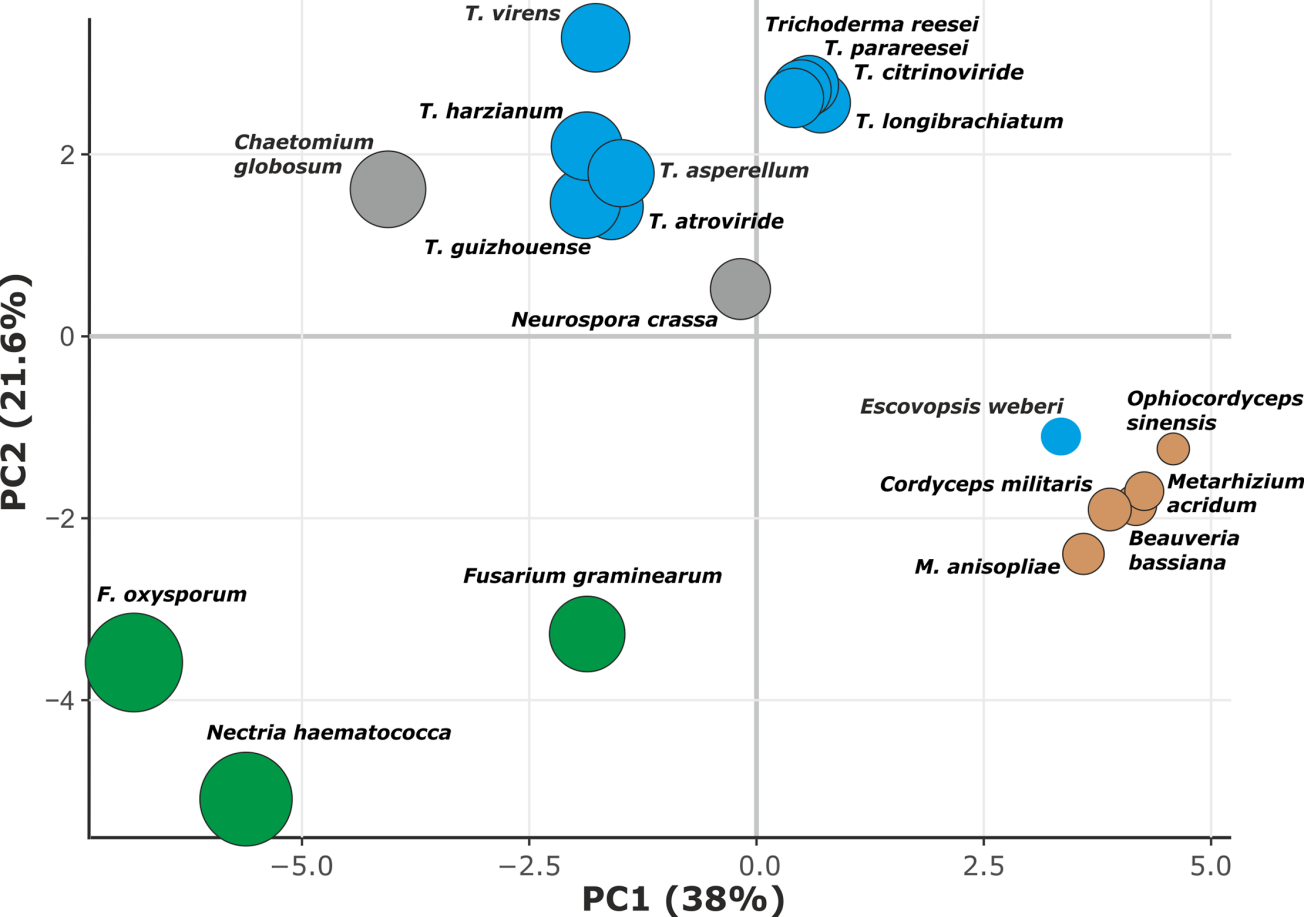
Principal component analysis based on the diversity of Hypocreales genes in GH families involved in plant cell wall degradation. Size of the dot corresponds to the total size of pcwdCAZome as shown in Fig 1B. Brown, blue, and green colors indicate parasitism on insects, fungi, and plants, respectively. Saprotrophic fungi are shown in grey.

For this comparison, we counted genes encoding enzymes for 29 of the GH families in other Hypocreales genomes [22, 28–36] (Supporting Information S3 Table). The unrelated polyphagous fungi, *Neurospora crassa* [37] and *Chaetomium globosum* [38] (both Sordariales, Ascomycota), were used as outgroups. A comparative analysis of these fungi showed that the pcwdCAZomes of phytoparasitic *Fusarium* and *Nectria* spp. are significantly larger than those of the entomoparasitic and mycoparasitic lineages, including *Trichoderma*. However, the cluster analysis revealed similarities between the pcwdCAZome composition of the mycoparasitic *E*. *weberi* and that of the entomoparasites, but not that of *Trichoderma* (Fig 1B). The latter genus possessed a pcwdCAZome that was more than twice as large as that of *E*. *weberi*. A principal component analysis (Fig 2) separated the pcwdCAZomes of *Trichoderma* spp. from those of *E*. *weberi* and the entomoparasites. Interestingly, the pcwdCAZomes were also separated from the phytoparasitic Nectriaceae. The *Trichoderma* pcwdCAZome exhibited closest similarity to the taxonomically distant fungi *N*. *crassa* and *C*. *globosum*. These data, therefore, do not support the hypothesis that the composition of *Trichoderma* pcwdCAZome is the ancestral state. Instead, it is likely the evolutionarily derived state.

### Evolution of the *Trichoderma* pcwdCAZome

To trace back the evolution of the *Trichoderma* pcwdCAZome, we collected the respective protein sequences encoded in all nine genomes and individually subjected each GH family (as well as PL1, AA9, and swollenin) to phylogenetic analysis. This examination revealed a total of 122 distinct phylogenetic groups of orthologous proteins (Supporting Information S3 Table). 61 were present in all nine species, fifty only in one or two *Trichoderma* sections, and 11 occurred only in a single species: four in *T*. *virens*, four in *T*. *atroviride*, two in *T*. *asperellum* and one in *T*. *harzianum*; no orphan pcwdCAZymes were found in species from the section *Longibrachiatum*. The largest pcwdCAZomes, possessing 91–99 proteins per species, were observed in the sections *Pachybasium* and *Trichoderma*, while genomes in the section *Longibrachiatum* encoded only 66–70 such proteins. These variable sizes of the pcwdCAZome were proportional to the changes in the total number of genes in their genomes, yielding a constant value of 0.6–0.8%. Thus, none of the nine *Trichoderma* spp. are therefore specifically enriched in genes required for plant cell wall degradation, which corresponds to a similar ecophysiology for these species (see above). Three GH families (GH26 ß-D-mannanases, GH51 α-L-arabinofuranosidase, and GH121 β-L-arabinobiosidase) were absent from the section *Longibrachiatum*. The highest diversity and quantity of respective proteins within *Trichoderma* were found in the GH3 (ß-glycosidase), GH27 (α-D-galactosidase), GH43 (α-L-arabinofuranosidase and ß-xylosidase), and GH28 (polygalacturonases) families (Supporting Information S3 Table).

**Fig 3:**
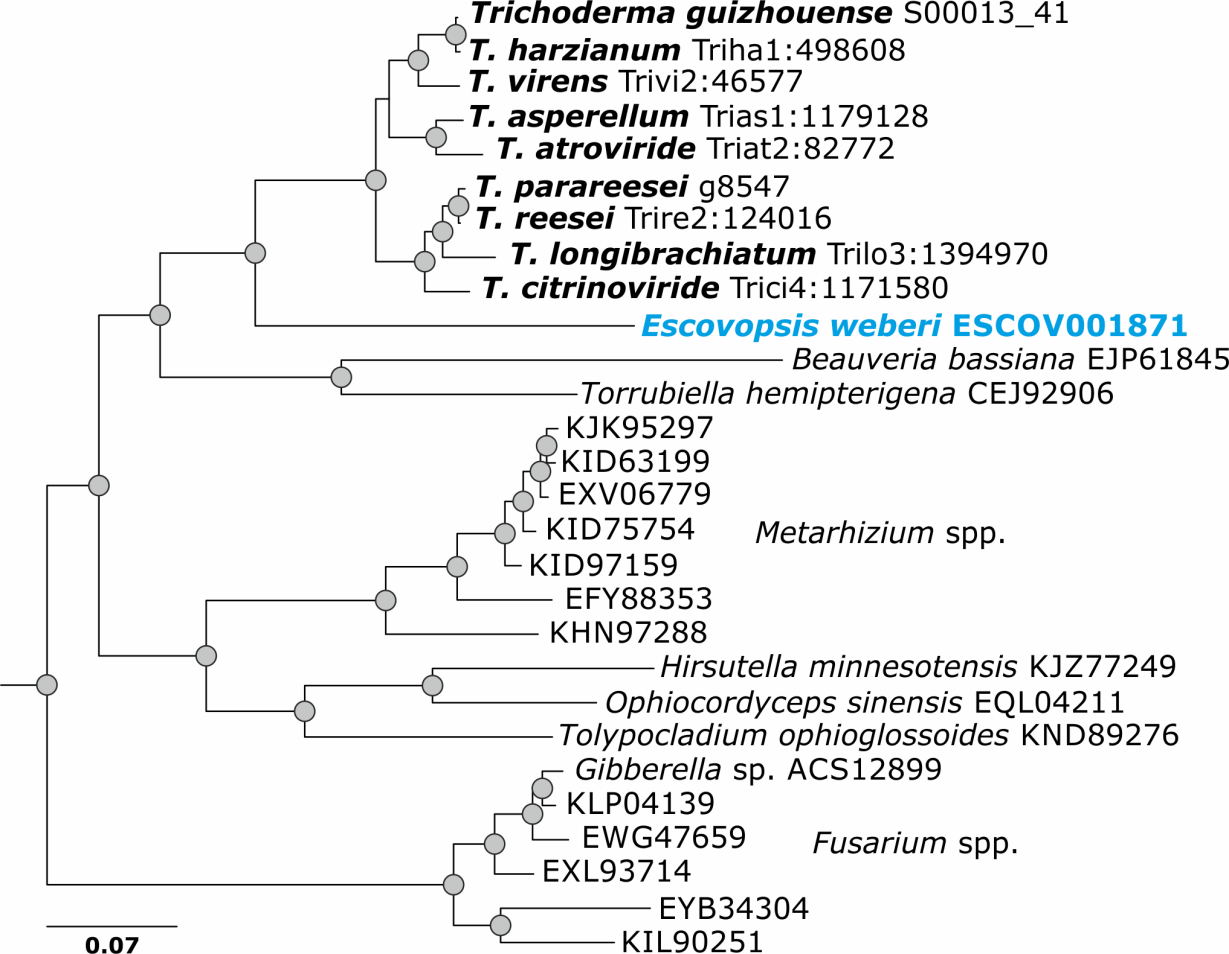
Evolution by vertical gene transfer of GH36 α-1,4-galactosidase Clade B (reference sequence Trire2:124016 of *T*. *reesei* QM 6a) in *Trichoderma*. Results for all pcwdCAZymes in *Trichoderma* are presented in Supporting Information S2 Fig.

**Fig 4:**
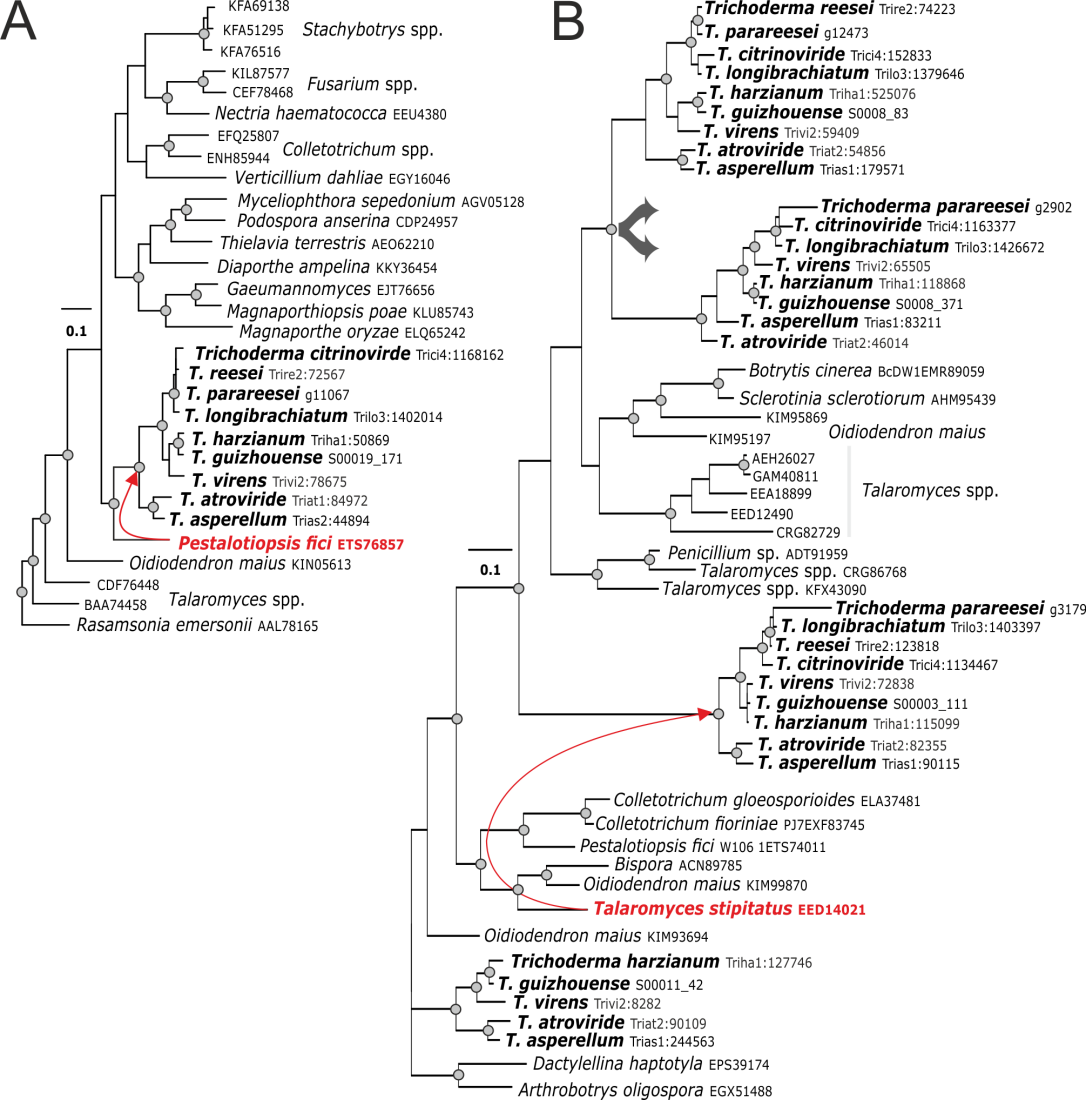
Evolution of selected pcwdCAZymes by putative lateral gene transfer. **A**. Evolution of GH6 cellobiohydrolase CEL6 (Trire2:72567) obtained by LGT from *Pestalotiopsis fici*. **B**. The GH11 endo-ß-1,4-xylanase gene (Trire2:74223) and its duplicated copies, which have incongruent tree topologies compared to the phylogenomic tree (see Fig 1A). *Talaromyces stipitatus* (Eurotiales) was confirmed to be an LGT donor for the clade containing Trire2:123818. The phylogenetic position of the GH11 clade including *T*. *atroviride* Triat2:90109 is unresolved (Supporting Information S2 Fig, S4 Table).

**Fig 5:**
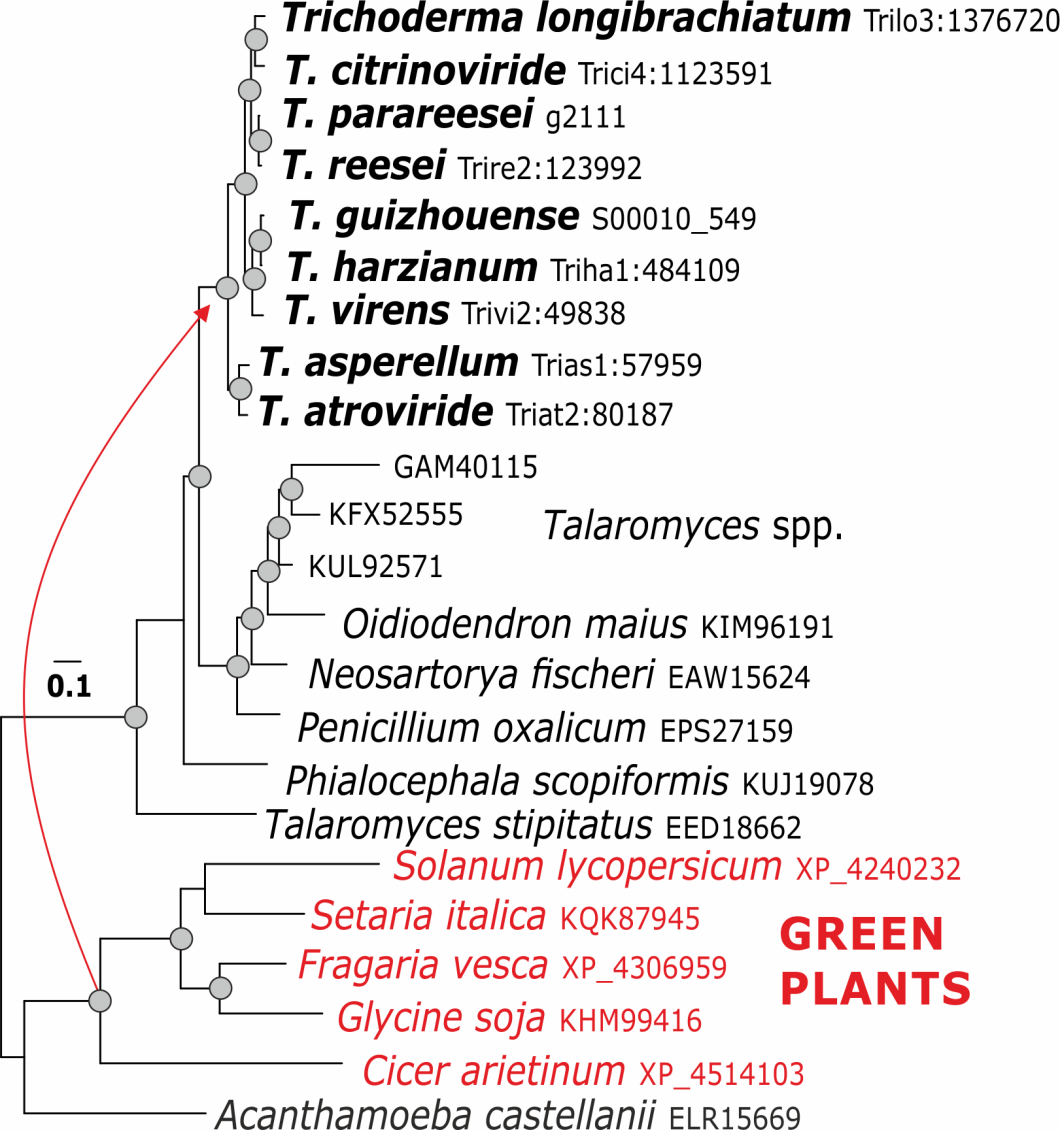
Evolution of swollenin in *Trichoderma*. The reference sequence Trire2:123992 of *T*. *reesei* QM 6a. Green plants have been identified as putative donors for LGT of this gene.

Representative sequences of each of the 122 phylogenetic groups (see above) were used as queries in a sequence similarity search in the NCBI Genbank database using the Blastp algorithm (see Materials and Methods for details). The hits with high sequence similarity (see description in Materials and Methods) were combined with the corresponding *Trichoderma* sequences from the nine species and subjected to phylogenetic analysis (Supporting Information S3 Table). When the topologies of the resulting 45 trees (Supporting Information S2 Fig) were compared to the phylogeny of *Trichoderma* (see Fig 1A for Hypocreales and Fitzpatrick et al. [39] for Ascomycota), only 29 (24%) of the 122 phylogenetic groups of *Trichoderma* pcwdCAZymes occurred at positions that were concordant with it (for example, GH36 in Fig 3). Among them, 16 were also present in the mycoparasitic fungus *E*. *weberi*. Thirteen phylogenetic groups of the pcwdCAZome (11%) belonged to clades that contained only *Trichoderma* proteins and, therefore, their evolutionary history remains unresolved. The phylogenetic position of the major part of pcwdCAZymes– 80 phylogenetic groups (66%)—was apparently not concordant with the evolution of the genus because these proteins shared last common ancestors with proteins of diverse Ascomycota fungi, such as phytoparasitic and phytophagous Eurotiomycetes, other Sordariomycetes, Leotiomycetes, and Dothideomycetes (for examples see Figs 4 and 5).

### Nearly half of the *Trichoderma* pcwdCAZome was obtained via LGT from lignocellulolytic Pezizomycotina fungi

**Fig 6:**
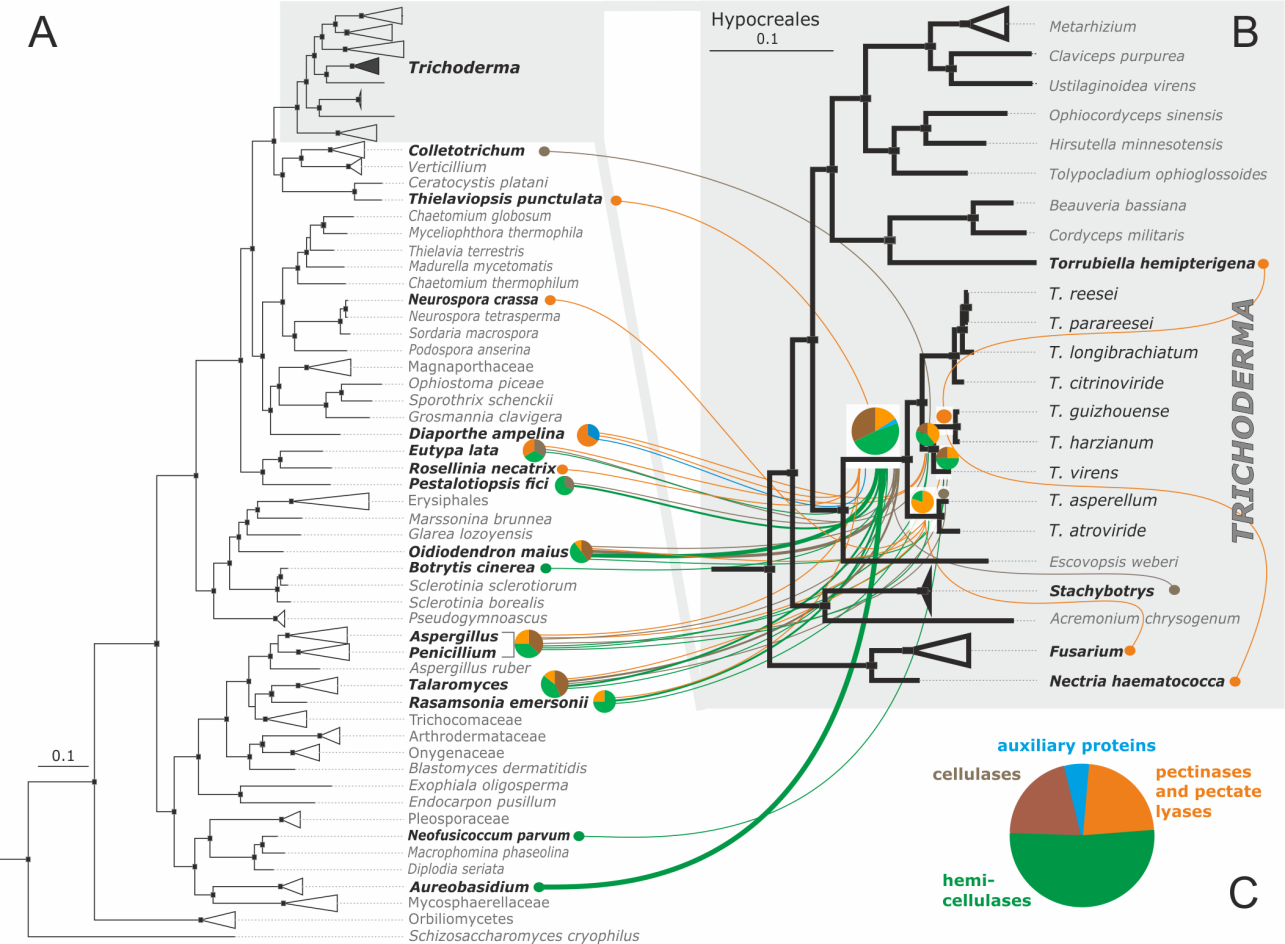
Evolutionary origin of *Trichoderma* pcwdCAZome obtained via putative LGT from Pezizomycotina donors mapped on Bayesian multilocus phylogram. **A**. The multilocus Bayesian phylogram of Ascomycota. **B**. The magnified Hypocreales clade from the phylogram on A. **A &B:** Black dots above nodes indicate posterior probability > 0.99. Individual lines correspond to LGT events, and the thickness of lines is proportional to the number of genes obtained from this donor. Statistically confirmed donor fungi are shown in bold. Colors correspond to the major groups of proteins composing the pcwdCAZome of *Trichoderma* (pie chart on **C**).

The incongruent topologies of the phylograms of individual pcwdCAZymes (Supporting Information S2 Fig) could be the result of gene duplication (GD), gene loss, or LGT. To distinguish among these possibilities, we reconciled each protein tree for each GH/AA9/PL1 family to the multilocus Ascomycota phylogeny shown in Fig 6 [40–42]. Using the approach of Wisecaver *et al*. [43], we assigned costs to GD, LGT and gene loss, and determined the most parsimonious combination of these three events to explain the individual pcwdCAZyme trees in view of the topology of the Ascomycota phylogeny (see Materials and Methods for details). Putative LGT events were only inferred when a CAZyme tree topology was contradictory to the Ascomycota phylogeny and could not be more parsimoniously reconciled by a combination of differential GD and gene loss. The respective NOTUNG results are given in Supporting Information S4 Table. This analysis suggested that at least 50 (41%) of the phylogenetic pcwdCAZome groups were obtained through LGTs from other fungi (Fig 6, Supporting Information S2 Fig & S4 Table). Most frequent putative donors were fungi from the order Eurotiales (16 cases), followed by the ericoid mycorrhizal fungus *Oidiodendron maius* (Leotiomycetes) (7 cases) and five cases for each of the cellulolytic Xylariales (three from *Pestalotiopsis fici* two from *Eutypa lata*), and three *Diaporthe ampelina* (Diaporthales) (Fig 6, Supporting Information S2 Fig & S4 Table). At the class level, donor fungi from Eurotiomycetes (16) and Sordariomycetes (15) were dominant, but transfers from Leotiomycetes (7) and Dothidiomycetes (2) were also detected. For one putative LGT event, the phytoparasitic *Colletotrichum* from the order Glomerellales (which is closely related to Hypocreales) was recognized as a donor. Thus, at least four putative cases of LGT to *Trichoderma* from Hypocreales fungi *Torrubiella*, *Stachybotrys*, *Fusarium*, and *Nectria*, respectively, have been detected (Fig 6, Supporting Information S2 Fig & S4 Table).

Surprisingly, no cases of LGT for pcwdCAZymes from Basidiomycota (which are the most commonly observed hosts/substrates for *Trichoderma in vivo* [4]) were detected, although they were present in several of the gene trees. Also no cases of horizontal gene transfer from prokaryotes were found. In our analysis, green plants were identified as putative LGT donors of the auxiliary protein swollenin for *Trichoderma* (Fig 5).

We also found three cases where LGT putatively occurred before the diversification of *Trichoderma* and *Escovopsis*, i.e. the major cellulase of *Trichoderma* (GH7; cellobiohydrolase CEL7A = CBH1), the GH 5 Endo-ß-1,4-mannnase and the pectate lyase PL1 (Supporting Information S2 Fig and S4 Table). The majority of genes obtained through LGT are present in all nine *Trichoderma* species and absent in *E*. *weberi*. Some pcwdCAZome genes are only present in sections of *Pachybasium* and *Trichoderma*, but not in the section *Longibrachiatum*.

**Fig 7:**
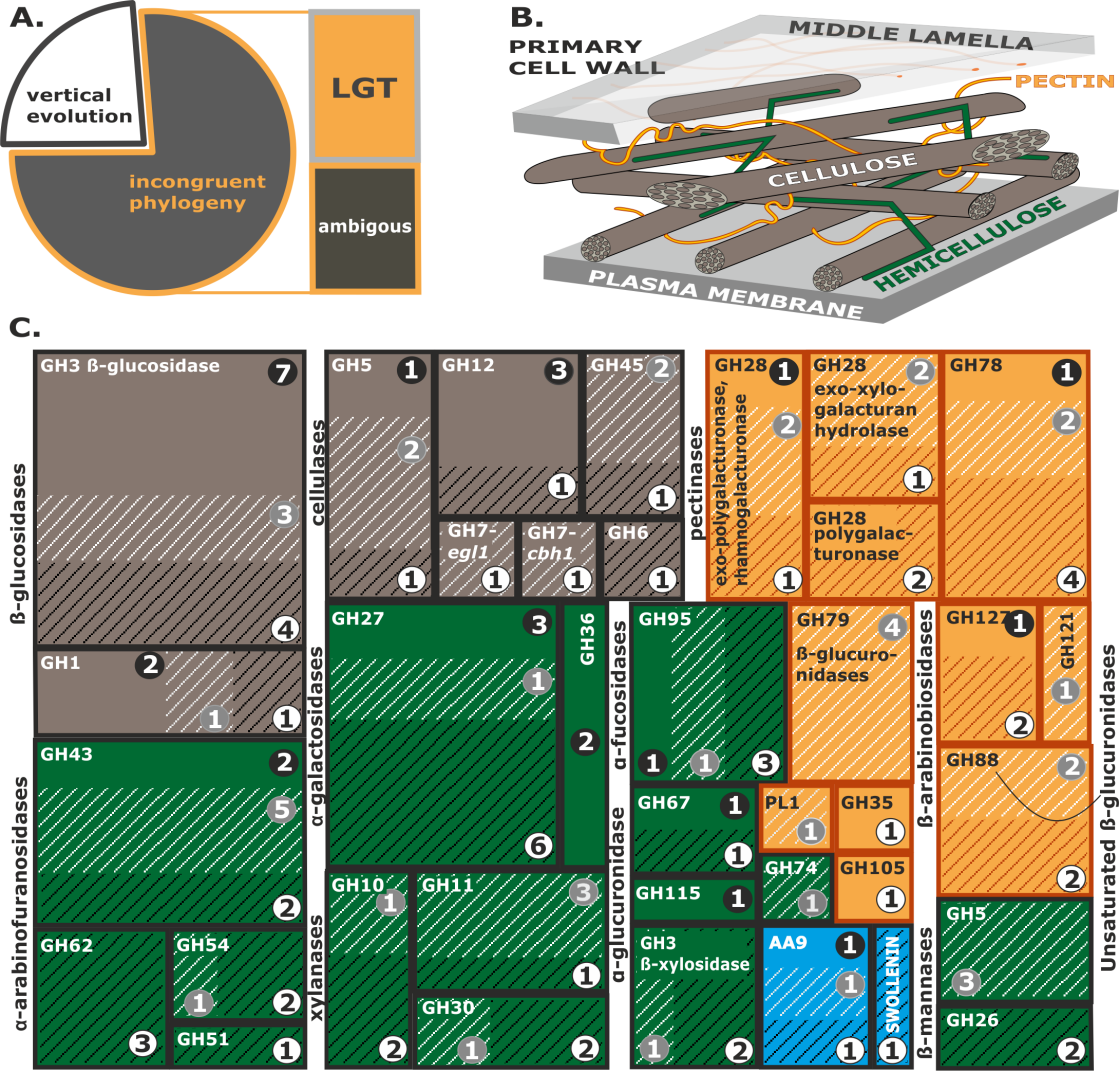
Composition and origin of the pcwdCAZome of *Trichoderma* based upon nine genomes. **A**. Summary of the evolutionary analysis and tests for LGT of individual proteins in *Trichoderma* pcwdCAZomes (N = 122) presented in Supporting Information S3 Table–S4 Table, S2 Fig. **B**. Schematic drawing of the primary plant cell wall. Cellulose, hemicellulose, and pectin are colored brown, green, and orange, respectively. **C**: The diversity and evolution of individual groups of *Trichoderma* pcwdCAZome. Brown, green, and orange rectangles correspond to enzymes involved in the degradation of cellulose, hemicellulose, and pectin, respectively. See B for the legend. Auxiliary proteins are shown in blue. Dark-shaded lines correspond to genes obtained through putative LGT, while light-shaded lines indicate additional cases of incongruent phylogeny and/or insufficient data. Numbers on white, black, or grey backgrounds correspond to the maximum total numbers of genes in each family that evolved through LGT, vertical evolution, or unknown mechanisms, respectively.

Members of four GH families (GH6, GH26, GH51, and GH62) seem to have entirely derived from LGT events (Fig 7), which correlates with the fact that these families are absent in the entomoparasitic Hypocreales species (Fig 1B). Twelve gene families exhibited a mosaic of vertical and lateral origin (Fig 7). In these, families with the highest proportion of LGT included the GH27 α-D-galactosidases, GH78 α-L-rhamnosidase, and GH95 α-D-fucosidases. Again, these are GH families that are absent from the entomoparasitic Hypocreales (Supporting Information S3 Table).

Twelve cases of putative gene duplications resulting in 24 genes were found, which comprised some cellulase (GH5, GH12, GH45), xylanase (GH10, GH11), and hemicellulase families (GH5 ß-mannanases, GH26, three in GH27 α-D-galactosidases, GH95 α-D-fucosidases, and GH28 exo-xylogalacturan hydrolases) (Supporting Information S2 Fig & S4 Table). Interestingly, many of them were present only in strains of section *Pachybasium* and *Trichoderma*, and in a few cases even only in a single species.

On a balance, considering the 29 vertically transmitted phylogenetic groups of pcwdCAZymes (including five gene duplication events that affected 10 of these genes), and the 50 phylogenetic groups that have been derived by LGT (among which 10 arose by five gene duplication events after a LGT event), we could putatively identify the evolutionary pattern of 79 phylogenetic groups (65%) of the *Trichoderma* pcwdCAZome. From the remaining 43 phylogenetic groups of pcwdCAZymes (35%) three also seem to have originated by LGT in the common ancestor of *Trichoderma* and *Escovopsis* (CEL7A, and GH5 Endo-ß-1,4-mannanase, and PL1 Supporting Information S2 Fig), thirteen (11%) formed isolated branches in the phylogenetic trees, and their origin cannot be determined. The remaining 28 (23%) phylogenetic groups of pcwdCAZymes exhibited tree topologies that were in conflict with the species tree, but not supported by NOTUNG analysis. Four of these genes (GH5) evolved by gene duplication (Supporting Information S2 Fig, S4 Table).

We also wondered whether any of the known regulatory proteins of *Trichoderma* pcwdCAZyme gene transcription (such as XYR1, ACE2, and ACE3) [4] would have been acquired via LGT. However, our results suggest that these genes evolved by vertical gene transfer and are present in non-lignocellulolytic entomoparasites and the mycoparasitic *E*. *weberi* (Supporting Information S2 Fig & S4 Table).

Because of the surprisingly large incidence of LGTs in the *Trichoderma* pcwdCAZome, we also tested whether other protein families would display such a high rate of LGT. To this end, we used a different approach: we screened the core genome of *Trichoderma* (consisting of about 7,000 orthologous genes that are shared among all *Trichoderma* spp. for which the genome sequences are available), but that are absent from genomes of *E*. *weberi* and other Hypocreales. This screen did not include the pcwdCAZyme encoding genes. This led to the identification of 738 genes, for which 123 genes had the nearest neighbors in blastp in Eurotiomycetes and various orders of Sordariomycetes that are taxonomically distant to *Trichoderma*. We emphasize that while these genes could have potentially been acquired by LGT—this conclusion is merely based on blastp the actual number of those genes actually derived by LGT is therefore most certainly smaller and in any case only speculative. However, it may constitute an upper limit of potential LGT events. Functional analysis showed that most of them encoded uncharacterized short-chain dehydrogenases and Zn_2_/Cys_6_ transcriptional regulators (Supporting Information S4 Table). Interestingly, we again could not detect basidiomycetes as putative donors of any of these genes. Cumulatively, this number of genes (123, see above) that could putatively have been obtained by LGT approximates only amount 1% of an average *Trichoderma* genome. It is in agreement with published estimations of 0.1–2.8% of LGT-derived genes for fungi [43] and significantly lower than that for pcwdCAZyme genes as reported in this paper.

### LGT events are not reflected in the clustering of pcwdCAZymes in *Trichoderma* genomes

LGT has frequently been shown to involve the transfer of large genomic fragments containing several genes [43]. Since a third of the *T*. *reesei* pcwdCAZome occurs in 20 discrete, loose clusters [25, 44], we tested whether these clusters are the consequence of LGT. An analysis of the synteny of the chromosomal loci of the above clusters in *T*. *reesei* with that in the other *Trichoderma* spp. showed that the clusters were highly syntenic (>80% of all gene positions were conserved), and this pattern was independent of their chromosomal location [45]. Thirty-three of the pcwdCAZymes of *T*. *reesei* were organized into a total of 16 clusters (Supporting Information S5 Table), but only 13 of these pcwdCAZyme genes had been acquired by LGT. In addition, the pcwdCAZyme genes in individual clusters were obtained from different donors. Therefore, we reject the hypothesis that the LGT-derived genes may have given rise to the origin of the CAZyme clusters proposed for *Trichoderma* [25].

### Alloparasitism of *Trichoderma* is complemented by parasitism on closely related Pezizomycotina, including adelphoparasitism on Hypocreales

Our analysis showed that *Trichoderma* phytophagy is indeed an apomorphic character that did not result from the convergent evolution of individual species or clades. Instead, it was obtained over the course of evolution through incidence of large-scale LGT. Putative donors include phytoparasitic fungi phylogenetically close to *Trichoderma* and possibly even neighboring groups. Interfungal interactions between *Trichoderma* and filamentous Ascomycota are rarely observed in nature [4]. However, the successful application of *Trichoderma*-based biofungicides against plant-pathogenic Ascomycota and respective studies of the roles of individual genes in mycoparasitism [46–55] support the hypothesis that such interactions take place alongside alloparasitism (parasitism on unrelated hosts) on Basidiomycota.

The possible cellular mechanisms for the uptake and incorporation of foreign DNA by fungi include conjugation, viral transduction, and conidial and hyphal fusion [56]. Although LGT between eukaryotes with cell walls has rarely been reported [57, 58], mycoparasitism has been viewed as a possible mechanism that could be linked to it [55, 59].

**Fig 8:**
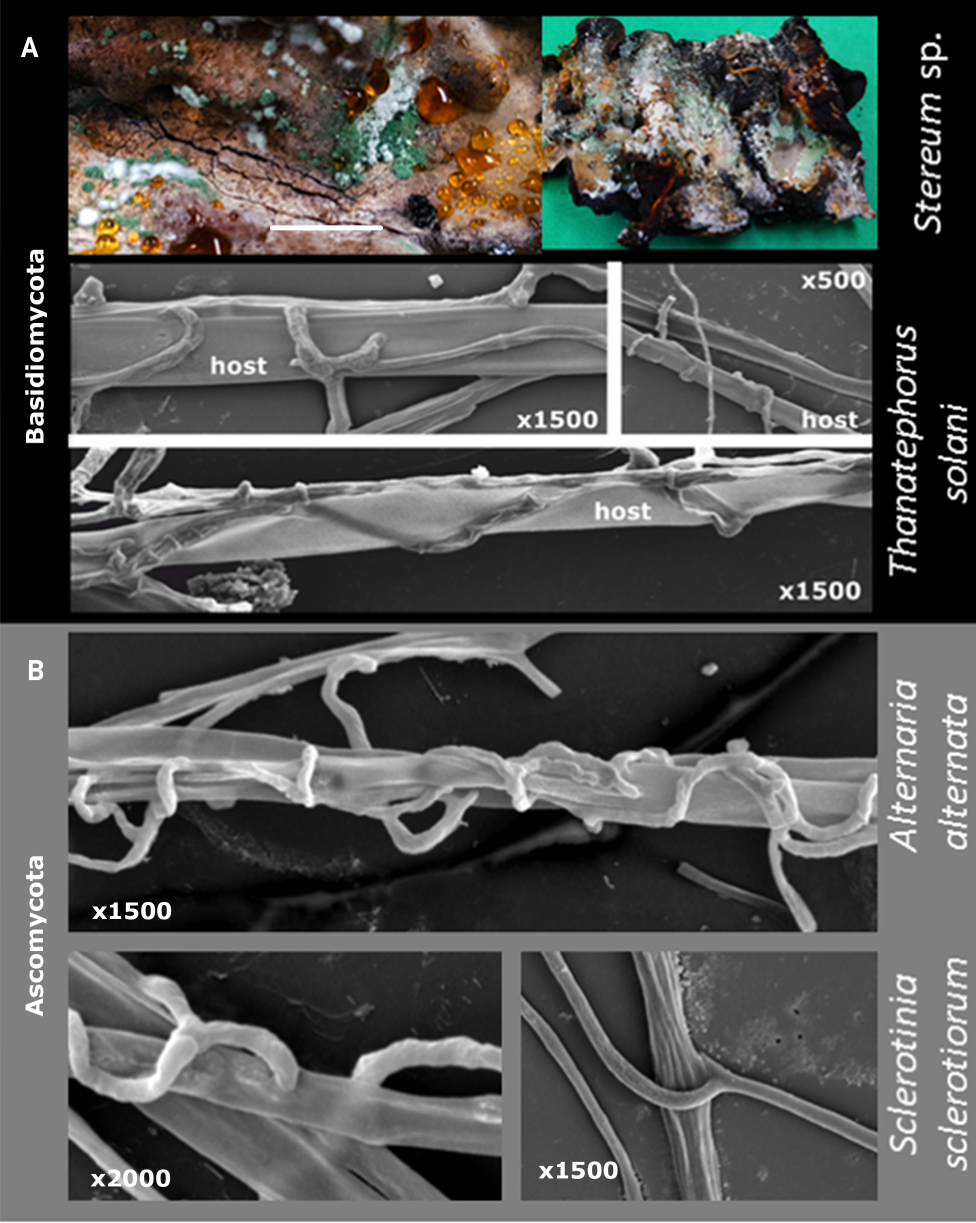
**Mycoparasitism of *Trichoderma* on Basidiomycota (A) and Ascomycota (B).** Macrophotography for **A** shows *T*. *simmonsi* TUCIM 6527 on *Stereum* sp. Bar indicates 1 cm. SEM images show hyphae of *T*. *guizhouense* NJAU 4742 on three hosts.

All fungi from the Hypocreaceae family are known to be aggressive alloparasites and they are common on sporocarps of Basidiomycota fungi *in situ* [4] (Fig 8A), *Trichoderma* spp. are effective against phytoparasites from Basidiomycota (for example [15, 26]), and the cause of the green mold disease on mushroom farms [60, 61]. In dual confrontation assays with colonies of *Lentinula edodes* (Agaricales, Basidiomycota), all *Trichoderma* spp. were able to parasitize this host, while *E*. *weberi* showed neither parasitism nor antagonistic reactions (Supporting Information S3 Fig). Similarly, all *Trichoderma* species were substantially more aggressive compared to *E*. *weberi* when confronted with its host fungus *Leucoagaricus gongylophorus* (Agaricales, Basidiomycota) (Supporting Information S3 Fig).

The evolutionary analysis of the pcwdCAZome of *Trichoderma* revealed LGT biased towards relatively close fungi (filamentous Pezizomycotina, Ascomycota). This selectivity could be explained by the ability of *Trichoderma* to parasitize Ascomycota fungi, which, in turn, is considered to be the major trait that sets *Trichoderma* apart from the other mycoparasitic Hypocreaceae fungi, such as *Escovopsis*, *Hypomyces*, and *Sphaerostilbella*, which parasitize Basidiomycota [16]. To test this hypothesis, we investigated the interactions of *Trichoderma* spp. with several model phytoparasitic Ascomycota. Scanning electron microscopy revealed similar interactions between *Trichoderma* hyphae and Basidiomycota (*Thanatephorus solani* [syn. *Rhizoctonia solani*, Cantharellales]) and Ascomycota hosts (Fig 8B), which include chasing, coilings, and penetration of the host hyphae.

**Fig 9:**
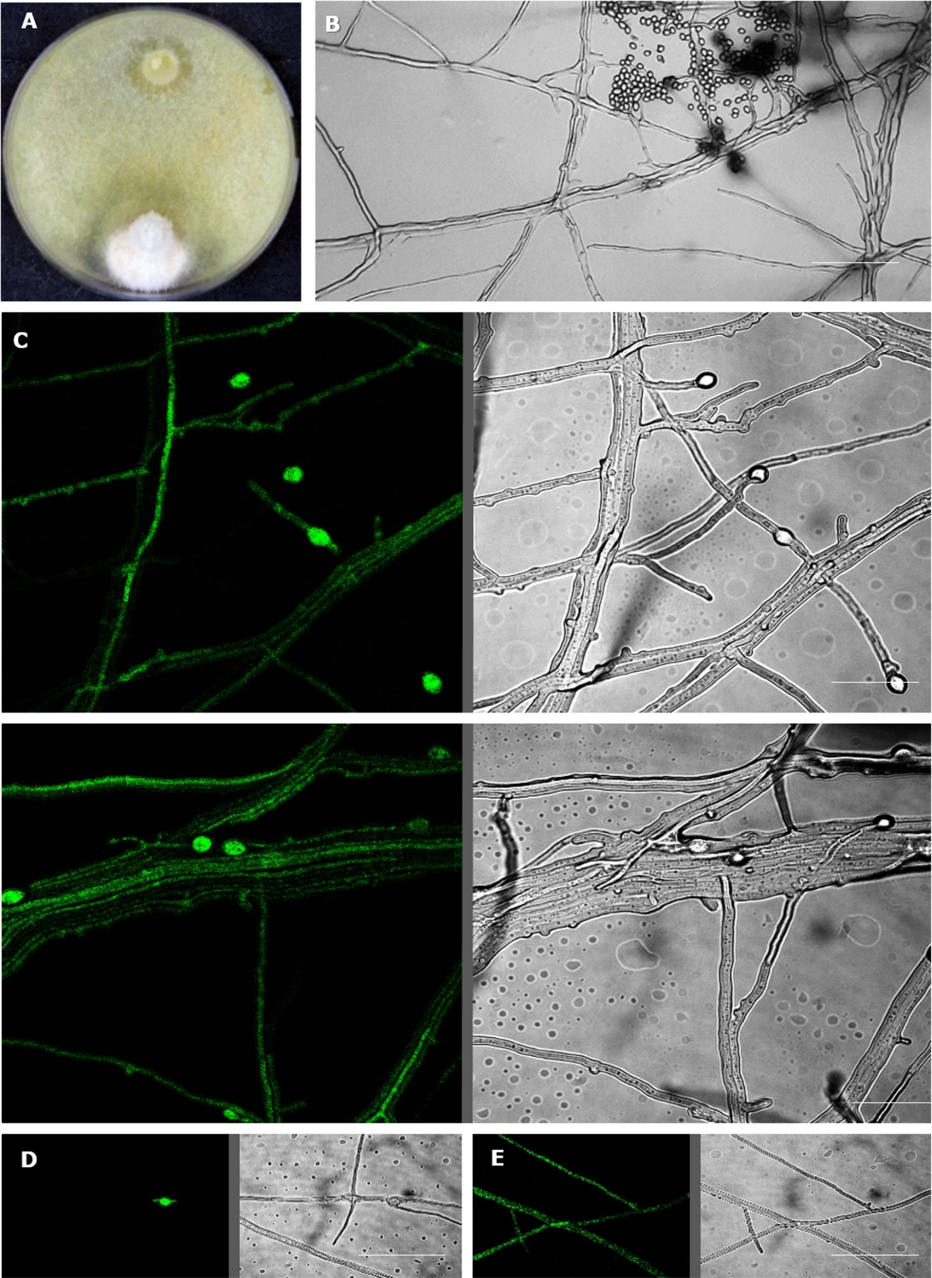
Mycoparasitism of GFP-labeled *T*. *reesei* TUCIM 4817 on *Pestalotiopsis fici* TUCIM 5788. **A**. Dual confrontation assay after 10 days of incubation at 28°C in darkness. **B**. Hyphal interactions observed using light microscopy (400x magnification). **C**. Confocal image showing endoparasitism of *T*. *reesei* on hyphae of *P*. *fici* on a glass slide prepared as shown in S3 Fig. **D.** Hyphae of *P*. *fici* TUCIM 5788 and a fluorescent chlamydospore of *T*. *reesei*. **E.** *T*. *reesei* TUCIM 4817 mycelium. Scale bar on C–E—40 μm.

We investigated interactions between *T*. *reesei* and the lignocellulolytic *P*. *fici* [24], which was several times identified as one of the putative LGT donors (Fig 6). *P*. *fici* was also selected because it has been isolated from the same ecosystem where *T*. *reesei* is common (the phyllosphere of *Shorea* sp., Borneo) and it has comparable growth rates *in vitro* (Supporting Information S1 Fig). In dual confrontation assays on agar plates, *T*. *reesei* overgrew a colony of *P*. *fici*, but did not kill it (Fig 9A). Microscopic examination revealed a tight association between the hyphae, suggesting endoparasitism of *P*. *fici* by *T*. *reesei* (Fig 9B). Confocal microscopy revealed that cords of *P*. *fici* hyphae were penetrated and colonized by the thinner hyphae of *T*. *reesei* (Fig 9C). This experiment shows that *Trichoderma* hyphae can grow inside hyphae of at least some extant putative Ascomycota donors. Dual confrontation assays with a set of randomly selected Eurotiales fungi showed that *Trichoderma* is capable of attacking these fungi as well (Supporting Information S3 Fig). However, endoparasitism was not observed, possibly because the hyphae of the tested Eurotiales fungi were comparable in size with *Trichoderma* spp., making internal penetration difficult.

**Fig 10:**
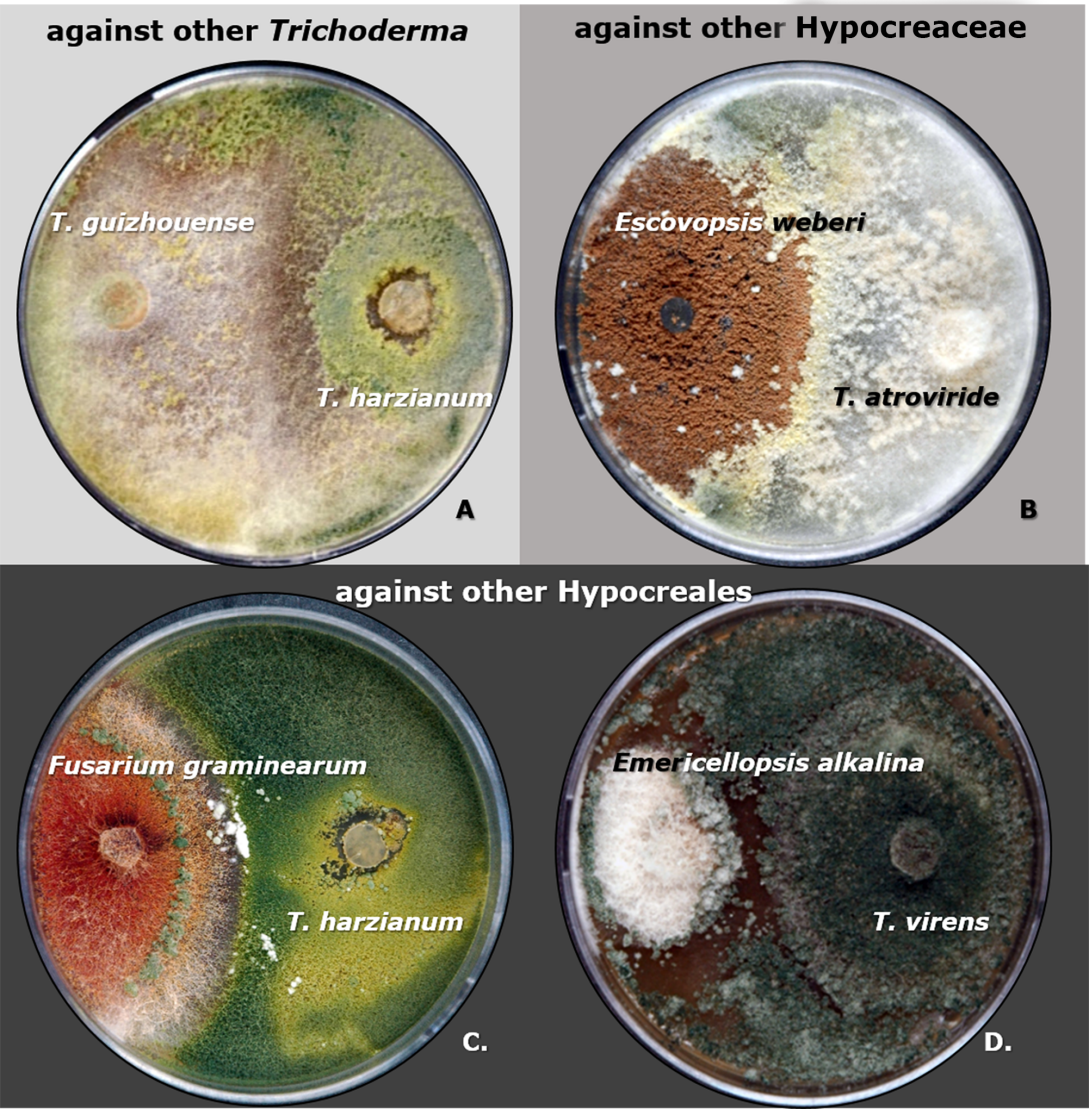
**Adelphoparasitism of *Trichoderma* on members of the same genus (A), same family (B), and same order (C, D).** Parasites were inoculated on the right side of each plate, and hosts are on the left side. Images were taken after 10 days of incubation at 28°C in the dark. Parasitism is assigned as a function of active overgrowth of the opponent colony. NCBI accession numbers for the DNA barcodes for fungi are given in Supporting Information S6 Table. Note to **A:** In this experiment, the host fungus *T*. *guizhouense* NJAU 4742 did not produce conidia (see other images in Supporting Information S3 Fig).

A surprising finding of this study was the detection of four cases of LGT of cellulolytic enzymes from other Hypocreales. Interestingly, *Trichoderma* is also capable of parasitizing fungi belonging to its very close phylogenetic neighbors (adelphoparasitism [62]), including *Fusarium* [55, 63]. To investigate the range of *Trichoderma* adelphoparasitism, we confronted different *Trichoderma* strains with fungi from the same genus, family, and order (Fig 10). The microscopic study revealed numerous cases of hyphal fusion that may be linked to self/non-self-recognition mechanisms in *Trichoderma* species and only in part to parasitism. Therefore, evidence for adelphoparasitism was only accepted when one colony overgrew the other. Our results showed that *T*. *harzianum* might attack its sister species, *T*. *guizhouense* (Fig 10A, see Fig 1 for phylogenomics). Any of the nine *Trichoderma* species can parasitize *E*. *weberi* (Fig 10B for *T*. *atroviride*), while the latter fungus did not attack *Trichoderma*. The majority of *Trichoderma* strains attacked and/or killed *Fusarium* spp. (Fig 10C) [55, 63], although individual strains of the latter host fungus resisted *Trichoderma* infections. A similar interaction was observed in a confrontation with *Emericellopsis alkalina*, which belongs to an *Acremonium* species complex in Hypocreales (Fig 10D). Our results show that all species of *Trichoderma* studied are capable of adelphoparasitism in the strictest sense of this term (parasitism on organisms belonging to the same genus or family [62]), and this property extends to interactions with other filamentous Ascomycota. Along with the unique ability to perform adelphoparasitism, *Trichoderma* maintains its alloparasitic properties (Supporting Information S3 Fig).

## Discussion

### LGT as an evolutionary shortcut to achieving nutritional versatility

In this work, we uncovered a possible evolutionary process that contributed to the development of the nutritional versatility of *Trichoderma*. Phylogenomic analysis showed that the genus shared a last common ancestor with entomoparasitic hypocrealean fungi (Cordycipitaceae, Ophiocordycipitaceae, and Clavicipitaceae). Since then, *Trichoderma* evolution has been directed towards mycotrophy. Although this path has also been taken by a number of other fungi of the family Hypocreaceae [20,55], *Trichoderma* is the most taxonomically diverse mycoparasitic fungus, harboring at least 260 molecularly defined species [64] found world-wide (NCBI Taxonomy browser, Nov. 2016). *Trichoderma* can also interact with animals [65], although the evolutionary state and mechanisms are not understood (see also Supporting Information S1 Text). It is known that the evolutionary history of some hypocrealean fungi involved the emergence of mycotrophy from a entomoparasitic/sarcophagic background. For example, *Elaphocordyceps* spp., deriving from the mainly entomoparasitic order Cordycipitaceae, are parasites of false truffles of the genus *Elaphomyces* (Eurotiales) [66].

Nikoh and Fukatsu [20] invoked the host-habitat hypothesis for such a “jump” from feeding on cicada nymphs to parasitism on truffles.

Due to the chemical composition of animals and fungi, the host shift from feeding on arthropods to feeding on fungi does not appear to be a difficult metabolic transition. Instead, it would only require a fine-tuning of ecological adaptations for specific hosts in one or another kingdom (i.e., mechanisms for recognition, defense, and overcoming the host). In contrast, feeding on plant biomass is an evolutionary challenge for any fungus specialized for feeding on insects or fungi. Our comparative analysis of the pcwdCAZome of hypocrealean fungi revealed that members of entomoparasitic families have a relatively poor repertoire of genes required for degradation of plant biomass compared to those of the hypocrealean phytoparasites. This paucity is also present in the *Escovopsis weberi*, a parasite of Agaricales and the closest phylogenetic neighbor of *Trichoderma* for which genome information is available [22]. The reduced number of pcwdCAZymes of *E*. *weberi* contradicts the predictions of the host-habitat hypothesis (see above) because the habitat of this fungus is directly linked to plant biomass, which is used by ants to cultivate *E*. *weberi*’s host fungus *Leucoagaricus* spp. The pcwdCAZome of the nine *Trichoderma* spp. investigated here was found to be of intermediate size between entomoparasitic and phytoparasitic Hypocreales fungi. We demonstrate that the abilities of *Trichoderma* to feed on plant and fungal biomass are equally developed in the studied species. Consequently, nutritional extension—not shifts or “jumps”—results in nutritional versatility and provides the basis for the general environmental opportunism of this genus [4].

### *Trichoderma* gained pcwdCAZymes from filamentous Ascomycota hosts

Our data suggest that nearly half of the genes encoding pcwdCAZymes have been obtained by LGT from other fungi. Gene duplication, which has been described as a major source of gene innovation in fungi [67] and other organisms [68] apparently played only a minor role in the evolution of the *Trichoderma* pcwdCAZome. It has been reported that 0.1–3% of the genes in a given Pezizomycotina genome were derived by LGT, usually indicating interdomain exchanges [43, 67]. When this estimation is applied to the 122 proteins of the pcwdCAZome of *Trichoderma*, maximally five genes would be expected to have originated from LGT. This suggests that the frequency of LGT in pcwdCAZome is an exceptional case. Surprisingly, we did not detect any transfer event from prokaryotes, and we also did not observe LGT events from Basidiomycota fungi. Marcet-Houben and Gabaldon [69] and Savory *et al*. [59] reviewed LGT events between bacteria and fungi, and listed *T*. *reesei* as one of the fungi comprising the highest number of bacterial-derived proteins. However, the genes transferred encoded arsenite reductases, catalases, different racemases and enzymes of peptidoglycan metabolism, but no pcwdCAzymes. Because the transfer of bacterial glycoside hydrolase genes to ciliates [70] or rotifers [71] has been demonstrated to have shaped their adaptation to polysaccharide-rich environments, we expected to find such cases for *Trichoderma*. However, none of the 50 LGT events detected in this study involved a bacterial donor.

The only example of non-fungal putative LGT to *Trichoderma* was that of the gene encoding the auxiliary protein swollenin [72, 73]. The plant expansins were described to have undergone at least two LGT events to other organisms, including one event that gave rise to amoebozoa expansins and fungal swollenins and another that gave rise to the bacterial expansins [73]. Our data are in accordance with these findings and further suggest that *Trichoderma* was among the first fungal genera to undergo LGT from plants (either directly or through other fungi).

### Which features of *Trichoderma* mycoparasitism may be linked to LGT?

Historically, LGT between eukaryotes containing cell walls has been considered to be rare and linked to phagotrophy [74]. However, nearly two decades ago, Wöstemeyer *et al*. [56] hypothesized that hyphal fusion mycoparasitism might offer nearly ideal conditions for interfungal DNA exchanges. They demonstrated the transfer of genes *in vitro* from the Mucoromycotina mycoparasite *Parasitella parasitica* to its Mucoromycotina host, *Absidia glauca* [75]. Our discovery that a massive but taxonomically restricted putative LGT of pcwdCAZymes occurred in *Trichoderma* from filamentous Ascomycota hosts correlates with the expansion of *Trichoderma* mycoparasitic host range to Ascomycota. This has not occurred in other Hypocreaceae (*Escovopsis*, *Hypomyces*, *Sphaerostilbella*, etc.) that feed on Basidiomycota. Chaverri and Samuels [16] proposed that the ability to parasitize Ascomycota is likely a dominant force that has driven diversification in *Trichoderma*.

In nature, alloparasitism (parasitism of taxonomically remote hosts) is widespread, while adelphoparasitism is rare. This has mainly been described as social parasitism in Hymenoptera (Arthropoda, Animalia), while cases of cellular interactions are limited to the Rhodophyta (red algae) *Gracilariopsis andersonii* and its closely related endoparasite, *Gracilariophila oryzoides* [76]. Interestingly, a case of adelphoparasitism has also been reported recently in Hypocreales for the clavicipitoid ergot parasite *Tyrannicordyceps sclerotium*, which attacks closely related species [66]. Contrary to nutritional expansions in *Trichoderma*, *T*. *sclerotium* offers an additional example of the apparently common nutritional shift, at least in Hypocreales.

The mycoparasitism of *Trichoderma* on Pezizomycotina has been intensively studied *in vitro* for its use in plant protection, and, therefore, these studies are biased towards plant pathogenic fungi that are not necessarily the natural hosts. In nature, *Trichoderma* has only rarely been observed on sporophores of ascomycetes from Xylariales and Helotiales [77]. We have investigated the interactions between *Trichoderma* and extant fungi that may represent or be descendants of ancient LGT donors. A particularly convenient model donor for *Trichoderma* spp. is *Pestalotiopsis fici* (Xylariales) because both fungi are ecophysiologically compatible *in vitro*. Interestingly, Gazis and Chaverri [78] found *Pestalotiopsis* and *Trichoderma* are the most frequent endophytic fungi on the leaves and stems of rubber trees (*Hevea brasiliensis*), which confirms their sympatric occurrence in nature and, thus, the possibility for LGT. Notably, in our experiments, *P*. *fici* was not killed by *Trichoderma*, although intrahyphal growth was observed.

Another very interesting finding of our study is the absence of putative LGT events of pcwdCAZymes from Basidiomycota fungi, which are common *Trichoderma* hosts or substrates in nature [4, 13] and on mushroom farms [61]. Our results and numerous previous observations show that *Trichoderma* is capable of penetrating the cell wall of fungi, such as *Thanatephorus solani* and *Athelia rolfsii* (Basidiomycota). This indicates that neither fusion mycoparasitism alone nor the host-habitat hypothesis predict interfungal DNA exchanges.

The reason why LGT from Basidiomycota was not detected is not easy to explain. This finding seems to not be restricted to pcwdCAZymes because we also found no hints of LGT from basidiomycete donors in other gene families. A single case of a putative LGT from Basidiomycota to *T*. *reesei* has been suggested by Slot and Hibbett [77] for the nitrate-utilizing gene cluster. Their analysis suggested *Ustilago maydis* (Ustilaginales) to be a donor. Interestingly, the Ustilaginales belong to the simple-septate Basidiomycota fungi, which do not have the complex dolipore septae found in Agaricomycotina mushrooms [79]. The dolipore may prevent the penetration of the host hyphae by *Trichoderma*. It could also be that LGT requires the growth of the parasite inside the host because during the proliferation of both hyphae, the cytoplasm and nuclei of both organisms may come in contact during mitosis. This might facilitate DNA exchange.

The likely physical difficulty to grow inside of hyphae of Basidiomycota with dolipores also suggests that mycoparasitic Hypocreaceae (*Escovopsis*, *Hypomyces*, *Sphaerostilbella*, etc.), which feed exclusively on such Basidiomycota, will not obtain genes from them. Indeed, such a case has not been reported thus far. Therefore, we hypothesize that the ability of *Trichoderma* to parasitize similar fungi (Pezizomycotina), even in extreme cases of adelphoparasitism, has been a significant ecological adaptation of this genus that subsequently enabled the observed putative LGT. We show that *Trichoderma* spp. can parasitize (overgrow and kill) some fungi belonging to the same genus, family, or order. In this study, we observed that *E*. *weberi* lacks this ability because it was parasitized by *Trichoderma* in all assays or did not interact.

The nutritional expansion of *Trichoderma* towards plant biomass through LGT is theoretically concordant with the “you are what you eat” concept in which the integration of a foreign DNA is a key mechanism. Yet, *Trichoderma* LGT resulted only from feeding on a limited group of hosts.

### Glycoside hydrolase requirements for feeding on plant biomass

We propose that the major putative LGT events that resulted in the nutritional expansion from more ancient mycoparasitism to phytophagy took place before the diversification of *Trichoderma* into extant infrageneric groups (sections, clades and species). It is also evident that the fungus maintained both nutritional strategies. Thus, the composition of the pcwdCAZome allows us to speculate about the requirements for efficient feeding on plant biomass. The GH families, for which genes have been entirely acquired by LGT and are absent from the phylogenetic neighbors of *Trichoderma* (i.e. GH6, GH51, GH62, GH74, and swollenin), reveal that improvements in cellulose and hemicellulose degradation were a key trait for the phytophagy of this fungus. Specifically, the gain of CEL6A that proceeds from the nonreducing cellulose ends complements the presence of CEL7A that acts at the reducing end, and therefore allows a processive movement along cellulose and an increase the speed of its degradation [80]. The addition of swollenin, which disrupts the cellulose structure and generates additional free chain ends [81], provides an increased number of accessible points for the two cellobiohydrolases. Interestingly, *Trichoderma* also obtained a large number of GH27 α-D-galactosidases, GH28 pectinases, and GH10, GH11, and GH30 xylanases, suggesting their importance for the hydrolysis of both hemicelluloses and pectin.

In this regard, it is meaningful that several GH families, that are absent from the entomoparasitic hypocrealean fungi, are present in *Trichoderma* and *E*. *weberi* (GH7A, GH5 ß-mannanases, GH12, GH67, GH74, GH95). This suggests that a part of the pcwdCAZome repertoire must have already been acquired before the split of the genera. It may also indicate that *E*. *weberi* likely lost the nutritional versatility of its ancestor along with a specialization for parasitizing *Leucoagaricus* spp. [22].

### Conclusions

In this study, we propose that the parasitism of *Trichoderma* on phylogenetically close hosts (up to adelphoparasitism) enabled LGT to build its unique pcwdCAZome and nutritional versatility. In support of this, *Trichoderma* spp. are frequently detected as a members of endophytic fungal communities [78] where they may either parasitize their putative cellulolytic hosts or feed on plant biomass or do both. Further studies of the evolutionary consequences of adelphoparasitism may explain other unique genomic features of *Trichoderma*. In addition, the description of the complete pcwdCAZome of nine *Trichoderma* spp. and, in the case of LGT, the identification of their putative donors may be of considerable interest to researchers studying the cellulolytic activity of this fungus for industrial applications.

## Materials and methods

### Organisms used in this study

*Trichoderma* strains used for the whole genome sequencing are given in Supporting Information S1 Table. All fungal strains and other organisms used in experiments and their respective accession numbers for DNA barcode sequences deposited in public databases and/or references are given in Supporting Information S6 Table.

### Assessment of the growth on plant and fungal biomass

For inoculum preparation, fungi were cultivated on potato dextrose agar (Sigma Aldrich, Steinheim, Germany) at 28°C for 4 days. Spore suspensions (3x10^6^ spores/ml) were prepared in 0.9% (w/v) NaCl with 0.025% (w/v) Tween 20 (Carl Roth, Austria). Growth tests were performed in CELLSTAR 24 Well Cell Culture plates (Greiner bio-one International). 1 ml of spore suspension of the fungus to be tested was inoculated on the following substrates: i) heat-treated (100°C for 3 hours) dried fruiting bodies of *Ganoderma lucidum* (Polyporales, Basidiomycota) (0.3% w/v), ii) epiphyte-free dried leaves of *Shorea johorensis* (Malvales, Angiosperms, Plantae) (0.3% w/v), iii) naturally degraded dead wood of *S*. *johorensis* (0.3% w/v), iv) commercial saw dust (local supplier, Vienna, Austria) (0.3% w/v), v) microcrystalline cellulose (0.05 mM research grade; AMS Biotechnology, Milton park, UK) in 0.5% (w/v) Agar-Agar Kobe I (Carl-Roth, Mannheim, Germany), vi) 2% (w/v) pre-treated (steam exploded) wheat straw 3% (w/v) in Agar-Agar Kobe I, vii) 0.3% pectin (w/v) in Agar-Agar Kobe I. Growth in 0.5% (w/v) Agar-Agar Kobe I was also tested as a control. All non-powdered substrates were finely ground and then sterilized at 120°C for 20 min. Experiment was carried out in quadruples. The plates were photographed after incubation at 28°C for seven days in darkness.

### Interfungal interactions

Dual confrontation assays between fungi were done as described in Atanasova *et al*. [15]. For these experiments, fungi were incubated for 10 days on PDA at 25°C and 12 hours with cyclic illumination. When required, slower growing fungi (such as *Lentinula edodes* and *Leucoagaricus gongylophorus*) were inoculated 2–3 days prior to the inoculation with fast growing Hypocreales. The set of *Penicillium* spp. and *Pestalotiopsis fici* TUCIM 5788 strains was randomly selected from a pool of strains isolated from phylloplane of *Shorea johorensis* (Dipterocarpaceae, Plantae) from Borneo where *Trichoderma* spp. are common.

#### Confocal microscopy

To analyze the interfungal interaction by confocal microscopy, spores of *T*. *reesei* strain TUCIM 4817, carrying a *gfp* gene under the control of a histone 3A promoter, and *Pestalotiopsis fici* TUCIM 5788 were inoculated on two adjacent but separated PDA agar blocks mounted between the glass slides and the cover glass using a modified Riddell slide method [114] (Supporting information S3 Fig). The construct was incubated for 72 hours in a sterile wet chamber that hyphae of both fungi established the contacts. Live-cell imaging was performed using a Nikon C1 confocal laser scanning unit mounted on a Nikon Eclipse TE2000-E inverted microscope base (Nikon GmbH, Vienna, Austria). A Nikon Plan Apo VC 100×/1.4 with oil immersion objective lens was used. GFP was excited with an argon ion laser at 488 nm. The emitted fluorescence was separated by a Nikon MHX40500b/C100332 filter cube and detected with a photomultiplier tube within the range of 500–530 nm. Bright light images were captured simultaneously with a Nikon C1-TD transmitted light detector mounted behind the condenser turret.

#### Scanning electron microscopy

For scanning electron microscopy, a coverslip (1 cm^2^) was placed on the centre of an agar plate inoculated with partner fungi and incubated until contact between hyphae was established (in average for 72 hours). The hyphae were then fixed with 2.5% (v/v) glutaraldehyde in 0.5 M potassium phosphate buffer and used for examining by SEM (HITACHI S-3000N, Tokyo, Japan).

### Genome analysis

The genomes of five *Trichoderma* species (*T*. *longibrachiatum* ATCC 18648, *T*. *citrinoviride* TUCIM 6016, *T*. *harzianum* CBS 226.95, *T*. *guizhouense* NJAU 4742 and *T*. *asperellum* CBS 433.97) were sequenced for this work (Supporting information S1 Table). Four of them *(T*. *longibrachiatum*, *T*. *citrinoviride*, *T*. *harzianum*, and *T*. *asperellum)* were sequenced using an Illumina platform. To this end, Illumina fragments (270 bp insert size) and 4 kbp long mate-pair (LMP) libraries were combined. The fragment libraries were produced from 1 μg of genomic DNA, sheared to 270 bp using an E210 Focused-ultrasonicator (Covaris) and size selection was carried out using SPRIselect (Beckman Coulter). The fragments were treated with end-repair, A-tailing, and ligation of Illumina adapters (Eurofins MWG Operon), using a NEBNext Ultra DNA Library Prep Kit (New England Biolabs Inc.).

Two types of LMP libraries were used, CLIP (Cre-Lox Inverse PCR) and LFPE (Ligation Free Paired-End), both of which used 15 μg of genomic DNA sheared with HydroShear (Genomic Solutions) using a selection size of 4 kb. For CLIP, the size selected DNA was ligated to adaptors containing loxP and the Illumina specific primer sequence. This adaptor ligated DNA fragments were then circularized via recombination by a Cre (NEB) excision reaction. The circularized DNA templates were then digested with a cocktail of four base cutter restriction enzymes, i.e. NlaIII, MseI, HypCH4IV (NEB), followed by self-ligation. The paired end library was then amplified via inverted PCR using an Illumina specific primer set. The size of the amplified paired end library was selected by running on a 1.8% (w/v) agarose gel followed by gel purification of the desired fragment (300–600 bp).

For LFPE library, the sheared DNA was treated with end repair adapters and ligated with biotinylated adapters. The adapter ligated DNA fragments were circularized by intra-molecular hybridization. The circularized DNA templates were digested by T7 Exonuclease and S1 Nuclease (Thermo Fisher Scientific). The digested fragments were treated with A-tailing Enzyme (NEB), followed by immobilization of mate-paired fragments on strepavidin beads (Thermo Fisher Scientific). Illumina compatible adapters (IDT, Inc) were ligated to the mate paired fragments and 12 cycles of PCR was used to enrich for the final library (KAPA Biosystems).

All prepared libraries were quantified using KAPA Biosystem’s next-generation sequencing library qPCR kit and run on a Roche LightCycler 480 real-time PCR instrument. The quantified libraries were then prepared for sequencing on the Illumina HiSeq sequencing platform utilizing a TruSeq paired-end cluster kit v3 and Illumina’s cBot instrument to generate a clustered flowcell for sequencing. Sequencing of the flowcell was performed on the Illumina HiSeq2000 sequencer using a TruSeq SBS sequencing kit 200 cycles v3, following a 2 x 100 bp or 2 x 150 bp run recipe.

Illumina data were QC filtered for artifact/process contamination and subsequently assembled using Rnnotator [82] for transcriptomes, and AllPathsLG [83] for genomes. The Pacific Biosciences library was prepared from 5 μg of gDNA sheared using a Covaris LE220 focused-ultrasonicator with their Blue miniTUBES to generate sheared fragments of 3kb in length. The sheared DNA fragments were then prepared according to the Pacific Biosciences protocol and using their SMRTbell Template Preparation Kit, where the fragments were treated with DNA damage repair, had their ends repaired so that they were blunt-ended, and 5’ phosphorylated. Pacific Biosciences hairpin adapters were then ligated to the fragments to create the SMRTbell template for sequencing. The SMRTbell templates were then purified using exonuclease treatments and size-selected using AMPure PB beads. Sequencing primer was then annealed to the SMRTbell templates and Version C2 sequencing polymerase was bound to them. The prepared SMRTbell template libraries were then sequenced on a Pacific Biosciences RSII sequencer using Version C2 chemistry and 2x45min sequencing movie run times.

Genomes were annotated using the JGI Annotation pipeline and made available via JGI fungal genome portal MycoCosm (jgi.doe.gov/fungi [84]). They have also been deposited at DDBJ/EMBL/GenBank as specified in Supporting Information S1 Table.

The genome of *T*. *guizhouense* NJAU 4742 was shotgun sequenced using a Roche 454 GS FLX system at the Chinese National Human Genome Center (Zhangjiang Hi-tech Park, Shanghai, China) with 28.4X coverage. The fragment libraries were produced from 5 μg of genomic DNA, sheared to 300–500 bp using M220 Ultrasonicator (Covaris, America) and was purified with Agencourt Ampure beads (Beckman, America). The fragment libraries were constructed with Purified DNA fragments by using DNA Library Preparation kit (Roche Applied Science, Switzerland) and fixed on magnetic beads with GS emPCR kit (Roche Applied Science). The 569 Mb raw data were achieved from 454 GS FLX system with 1,435,699 reads.

For sequence scaffolding, Solexa Mate Pair reads were used to establish the genome scaffolds. 5 μg of genomic DNA was sheared with a Hydroshear device (Gene Machine) to generate 3–5 kb DNA fragments. The library was prepared by using TruSeq DNA Sample Prep Kit-SetA (illumina, America), and amplified using TruSeq PE Cluster Kit (illumina, America), and then sequenced in Solexa sequencing machine (illumina, America). Gene calls were generated using FGENESH [85], ExonHunter [86] and AUGUSTUS version 2.7 [87].

### Composition of *Trichoderma* pcwdCAZome

Annotation of the genes encoding carbohydrate active enzymes involved in plant cell wall degradation (pcwdCAZome) in the nine *Trichoderma* genomes was performed using the Carbohydrate-Active Enzyme database (CAZy) nomenclature [88, 89], by comparing each protein model from the genome by the sequence similarity search tool (blastp) to a collection of protein modules corresponding to catalytic and carbohydrate-binding modules derived from CAZy. Individual hits were then compared by HMMer to models corresponding to each CAZy family to allow an assignment of each identified protein.

Accession numbers of genes composing pcwdCAZome and respective regulatory proteins in *Trichoderma* genomes are given in Supporting Information S3 Table.

Principal component analysis and two-way cluster analysis of the Hypocreales pcwdCAZomes (Supporting Information S3 Table) were made with the use of https://biit.cs.ut.ee/clustvis/ [90]. Cluster analysis was made with Euclidian distance and complete linkage method.

### Genomic location of individual genes encoding for pcwdCAZome

To analyse whether the genomic location of the identified pcwdCAZomes would be syntenic among the nine *Trichoderma* species, we used the manually annotated chromosomes of *T*. *reesei* [45] as a template. Orthologs for each individual gene from the other *Trichoderma* spp. were then located on their genomic scaffolds, and at least five genes flanking its 5’ and 3’ area were retrieved and identified by blastp. A synteny value of 100% was assigned between *T*. *reesei* and another *Trichoderma* sp. when all investigated flanking genes were orthologues and their order was conserved.

### Phylogenetic analyses

#### Phylogenomic analysis of *Trichoderma* and other Hypocreales using 100 neutrally evolving genes

One-hundred genes were randomly selected from the genomes of the nine *Trichoderma* spp. and 12 reference Hypocreales (*Escovopsis weberi*, *Metarhizium acridium*, *M*. *robertsii*, *Calviceps purpurea*, *Ophiocordyceps sinensis*, *Beauveria bassiana*, *Cordyceps militaris*, *Fusarium graminearum*, *F*. *oxysporum* f. sp. *lycopersici*, *F*. *pseudograminearum*, *F*. *fujikuroi*, and *Nectria haematococca;* see Supporting Information S1 Table and S2 Table) based on two requirements: (a) they should display a syntenic position in all genomes, and (b) be true orthologues (no other gene encoding a protein with amino acid similarity >50% present). *Neurospora crassa* and *Chaetomium globosum* (Sordariales) were chosen as outgroups. For each gene the alignments of nucleotide sequences consisting of coding regions were prepared using ClustalW [91] and analyzed for the neutral evolution [92] using DNASp V5.10.01 [93] based on Tajima’s D test [94]as described by Rozas [95] (Supporting Information S2 Table). Multiple sequence alignments of each protein were done using ClustalW [91]. Resulting alignments were examined in Genedoc [96] and then subjected to the phylogenetic analysis online in PhyML [97] based on best amino acid substitution model acquired using “smart model selection” option (http://www.atgc-montpellier.fr/phyml-sms/). Maximum likelihood trees assessed using 1000 bootstrap replicates were also constructed individually for each of the 100 protein sequences and the phylome is deposited at http://itol.embl.de/shared/druzhininaetal. Accession numbers of all genes used in phylogenomic analysis are given in Supporting Information S2 Table.

For a combined analysis, a concatenated set of 100 proteins for each of 23 species was subjected to the alignment algorithm using the stand alone MAFFT tool [98] with G-INS-i parameters. Selection of conserved blocks was done using “relaxed” conditions in Gblocks [99]. The final concatenated alignment contained 47 726 amino acids. The selection of best amino acid substitution model was done using ProtTest 3 [100] based on BIC criterion. The Bayesian analysis was performed using MrBayes v3.2.5 [101, 102], 1 million generations and the Dayhoff I+G+F amino acid substitution model [103]. Two simultaneous, independent analyses starting from different random trees were run, each using three heated chains and one "cold" chain. Once the analyses were completed, 7500 trees were summarized after discarding the first 25% of the obtained 10,000 trees, resulting in a consensus tree. The parameters of phylogenetic analyses and accession numbers of individual genes are given in Supporting Information S2 Table.

#### Multilocus phylogeny of Ascomycota

To reconstruct a phylogenetic tree that included also all fungi for which putative pcwdCAZyme homologs to *Trichoderma* have been identified (see below), the amino acid sequences from four nuclear genes that have previously been shown to be suitable phylogenetic markers for Ascomycota multilocus phylogeny (histone acetyltransferase subunit of the RNA polymerase II holoenzyme, FG533; NAD-dependent glutamate dehydrogenase, FG570; translation initiation factor eIF-5, FG832; and TSR1p, a protein required for processing of 20S pre-rRNA, MS277) were retrieved from FunyBase [104] (http://genome.jouy.inra.fr/funybase), GenBank (http://www.ncbi.nlm.nih.gov/genbank/), the Joint Genome Institute (http://genome.jgi-psf.org/programs/fungi/index.jsf?projectList), EnsemblFungi (http://fungi.ensembl.org/index.html) and Broad Institute (http://www.broadinstitute.org/) databases. Complete sets of amino acid sequences for 128 fungi (including the nine *Trichoderma* spp.) were prepared. Concatenated alignments (provided in Supporting Information S1 Data) and an MCMC analysis using MrBayes v3.2.5 was performed as described above. Accession numbers of individual genes from Ascomycota fungi are given in Supporting Information S7 Table.

In the case of swollenin, where the best Blastp hits included plant species, an appropriate species tree was constructed using the NCBI Taxonomy Browser.

#### Phylogenetic analysis of individual proteins from the *Trichoderma* pcwdCAZome

Protein sequences from the nine species that belonged to the same GH family were aligned using CLUSTALW [88] and subjected to phylogenetic analysis with PhyML embedded in TOPALI v 2.5 [105]. To retrieve the respective closest pcwdCAZyme neighbors from other fungi, one or more *Trichoderma* proteins from each GH protein family (including PL1 and AA9 auxiliary proteins) were first subjected to a sequence similarity search by blastp against the NCBI database (finished by December 28, 2015). All hits with a query coverage of >90% and an E-value < 10^−100^ were collected. We initially used a less stringent E-value (< 10^−40^), but found that the validated close neighbors were all characterized by < 10^−100^. When this analysis failed to retrieve orthologs from closely related species, the analysis was repeated using tBlastn. Only published sequences were used. The final set of sequences was realigned using Muscle 3.8.425 [106] and integrated in the program Aliview [107]. Duplicate sequences from the same species were removed. Highly polymorphic regions were removed using the Gblocks [99] server with unconstrained parameters. The curated alignments were then subjected to evolutionary analysis using MrBayes v3.2.5 [102] as described above. The number of generations chosen for each phylogenetic tree depended on the number of sequences in the alignment. As a rule, the MCMC analysis was run for 1 million generations for all the alignments containing less than 100 sequences, and these were subsequently increased by 1 million until the standard deviation of split frequencies of the two parallel yet independent runs fell under 0.05 in the case of alignments containing more than 100 sequences. Parameters of individual phylogenetic analyses are given in Supporting Information S3 Table.

#### Inferring horizontal gene transfer, gene duplication and gene loss

The incongruent topologies of the phylograms of individual proteins from the pcwdCAZome compared to the topology of the Hypocreales phylogenomic tree and the multilocus phylogram of Ascomycota fungi could be the result of gene duplication (GD), gene loss, or LGT. To distinguish between these possibilities, we reconciled the protein trees of each GH/AA9/PL1 family to the multilocus Ascomycota phylogeny (Fig 6A) in NOTUNG [40–42]. Using the approach of Wisecaver *et al*.[43], we assigned costs to GD, LGT, and gene loss and determined the most parsimonious combination of three events to explain the individual pcwdCAZome trees in view of the topology of the Ascomycota phylogeny. An edge weight threshold of 0.9 was applied. To find the most appropriate parameters, we evaluated three different ratios of transfer to GD costs (2, 4 and 6) and compared the predicted gene transfers to those obtained by T-Rex [108]. The latter infers LGT by quantifying the proximity between two phylogenetic trees using a refinement of the Robinson and Foulds distance using midpoint rooting (see Supporting Information S4 Table). Finally, we used transfer costs twice as much as GD cost because it was the lowest ratio that identified LGT events that were in agreement with those suggested by the discordance of species and protein phylogeny. LGT events were only inferred when a CAZyme tree topology was contradictory to the Ascomycota phylogeny and could not be more parsimoniously reconciled by a combination of differential GD and gene loss. The scores are given in Supporting Information S4 Table.

## Supporting information

S1 Text Ecological terminology used in this study to describe types of nutrition found in Hypocreales fungi. (PDF)

S1 Table Properties of fungal genomes that were used in this study. (XLSX)

S2 Table Materials describing phylogenomic analysis of Hypocreales based on 100 orthologous proteins. A. Annotations of 100 orthologous proteins used in phylogenomic analysis and results of the neutrality tests. B. Protein accession numbers for 23 genomes.
(XLSX)

S3 Table Composition and evolution of pcwdCAZome of *Trichoderma* and related fungi. A. NCBI Accession numbers of genes composing the pcwdCAZome of *Trichoderma* and regulatory proteins analyzed in this study. B. Distribution of pcwdCAZymes in GH families in Hypocreales. C: Parameters of phylogenetic analyses of individual proteins from *Trichoderma* pcwdCAZome.
(XLSX)

S4 Table Results of statistical tests of the LGT hypothesis. A. The transfer costs for NOTUNG and comparison to LGT events predicted by T-REX. B: Results of the NOTUNG analysis of pcwdCAZome and relevant regulatory proteins. C. Summary on the evolutionary origin of pcwdCAZome of *Trichoderma* inferred in this study. D. Functional annotations of core genome *Trichoderma* genes that have no orthologous copies in other Hypocreales genomes.
(XLSX)

S5 Table Chromosomal location of individual genes from pcwdCAZome of *T*. *reesei*. (XLSX)

S6 Table Additional organisms used in this study. (XLSX)

S7 Table Accession numbers of genes used for the multilocus phylogeny of Ascomycota fungi. (XLSX)

S1 Fig Growth of *Trichoderma* spp., *Escovopsis weberi* and *Pestalotiopsis fici* on natural substrates resembling polymers in the fungal and plant cell walls. Strains were evaluated after 10 days of incubation at 28°C in darkness. Yellow, green and white shape outlines correspond to good, weak and no growth, respectively. Data are representatives of four separate experiments.
(PDF)

S2 Fig Phylogenetic trees of *Trichoderma* plant cell wall-degrading carbohydrate active enzymes and regulatory proteins. (PDF)

S3 Fig Mycoparasitism of *Trichoderma*. A. Allomycoparasitism of *Trichoderma* spp. and *E*. *weberi* on *Lentinula edodes*. B: Allomycoparasitism of *Trichoderma* and *E*. *weberi* on *Leucoagaricus gongylophorus*. The dashed lines indicate growth of the host fungus as deduced from back sides of the plates. C: Set up for the microscopic investigation of *Trichoderma* (right) parasitism on *Pestalotiopsis fici* (left). 2 x 2 cm agar plugs were located between a sterile microscopy glass slide and a 5 x 2.5 cm sterile glass cover slip and aseptically inoculated with spores of two partner fungi, respectively, using a microbiological needle. Inoculated cultures were maintained at 28°C in wet chamber until hyphal contact. Microscopic investigation was done for hyphae on the cover slip surface. D. Antagonism of selected *Trichoderma* species on *Penicillium* spp. Dashed line indicates growth of the opponent fungus.
(PDF)

S1 Data Multiple sequence alignment used for the multilocus phylogeny of Ascomycota fungi. (AA)
