# Supplementary material for: Massive lateral transfer of genes encoding plant cell wall-degrading enzymes to the mycoparasitic fungus Trichoderma from its plant-associated hosts
Source: PLoS Genet. 2018 Apr 9;14(4):e1007322. doi: 10.1371/journal.pgen.1007322 (PMC5908196; doi:10.1371/journal.pgen.1007322)
Supplement: S1 Text — (PDF) [file pgen.1007322.s001.pdf]

Feeding types of filamentous Ascomycota fungi with a special note regarding the orders Hypocreales and *Trichoderma*

The history of fungi initially being considered plants contributes to the considerable confusion regarding the use of botanical, zoological, and microbiological terms when describing fungal biology, such as terms for the assignment of their feeding types.

The Kingdom of Fungi comprises mainly sessile heterotrophic organisms that rely on absorptive nutrition [1]. It is now confirmed that they share a last common ancestor with animals, which existed at least one billion years ago [2] or even earlier [3].

To avoid confusion related to terminology, we specify the meaning of the technical ecological terms used in this article (Table A). We follow the scheme of Getz (2012) [4], which is based on the distinction between (i) **eating live** or **dead biomass** and (ii) **consumers of animals, plants, or fungi**. In this article, it is also essential to distinguish between **generalists** (capable of feeding on any of listed resources) and **specialists** (feeding on a particular food source). Moreover, following the suggestion of Getz (2012), we attribute filamentous Ascomycota fungi to **miners** because he describes them as "relatively sessile in locally exploiting a resource mass larger than themselves" compared to **gatherers**, which are "relatively mobile in searching out and consuming or sequestering packets of resources." Because the terms indicating feeding types of gatherers are based on the Latin "*vorus*" (to swallow), in this article we avoid using such terms as carnivorous, fungivorous, and herbivorous. However, we should note that the above-mentioned terms are frequently applied to fungi [5-8].

We use the term **parasite** to refer to any organism that feeds on a <u>live biomass of any type</u>. Mechanisms of interactions, interactions types, and benefits and losses for individual partners are not considered here. Consequently, frequently used terms referring to pathogenicity (entomopathogen, plant pathogen, etc.) are not used. Feeding on dead biomass is described using terms based on the Greek word "*phagos*" (to eat), while the terms based on the Greek word "*trophe*" (food, nourishment) are used to describe the act of feeding on life or dead biomass.

We also note that the term "**environmental opportunist**," which has been recently attributed to some *Trichoderma* species [9], does not specifically mean nutritional versatility. It also includes the ability to rapidly grow and resist environmental stresses. Consequently, the term "**generalist**" is used for nutritional versatility on dead or live biomass, while "parasite" and "polyphags" describe each of the latter two types of biomass, respectively.

Table A. Terms describing feeding types of filamentous Ascomycota fungi

State	Resource*	Term indicating nutrition type	Comments	References for *Trichoderma*
**Live**	**Parasite**			
Live	**Insects sensu lato**	Entomoparasite	Here a colloquial meaning of insects is used: insect may apply to any small arthropod similar to an insect including spiders, centipedes, millipedes, etc	Moths [10], aphids [11], bed bugs [12], corn borer [13]
Live	**Fungi**	Mycoparasite	May also include necrotrophic parasites of fungi	Broad spectrum [9, 14-19]
Live	**Plants**	Phytoparasite	The term may also include plant pathogenic organisms, croppers and also endophytes as symptomless parasites of plants. Note: In this article, we do not use the meaning of this term sensu Merriam Webster Medical Dictionary https://www.merriam-webster.com/medical/phytoparasite [Oct. 2017]: "a parasitic plant".	Mainly endophyte [20-25] , rarely plant pathogen [26]
Live	**Plants and/or fungi and/or animals**	Parasite	Feeding on live biomass of any type, biotrophy	Immunocompromised and immunocompetent humans [27, 28], nematodes [29-31]
Dead	Greek: *phagos* = eat			
Dead	**Insects sensu lato**	Sarcophage, necrophage	May include necrotrophic parasitism	Moths [32], aphids [33], bed bugs [34], corn borer [35]
Dead	**Fungi**	Mycophage	Also includes necrotrophic mycoparasites	[9]
Dead	**Plants**	Phytophage, Saprophytophage	In this article mainly organisms feeding on non-wooden biomass were studied; xylophagous fungi capable to degrade lignin were not considered.	Dead wood and herbaceous biomass[9, 36-38]
Dead	**Plants and/or fungi and/or animals**	Polyphage	Saprotrophic nutrition	[9]
Live/Dead	**Fungi**	Mycotrophy	All kinds of feeding on fungi and fungal biomass	
Live/Dead	**Animal, fungi and plants**	Nutritional versatility, Generalism	If at least two types of resources may be equally well consumed	

References:

1. Moore RT. Taxonomic proposals for the classification of marine yeasts and other yeast-like fungi including the smuts Botanica Marina. 1980;23(6):12.
2. Lucking R, Huhndorf S, Pfister DH, Plata ER, Lumbsch HT. Fungi evolved right on track. Mycologia. 2009;101(6):810-22. Epub 2009/11/26. PubMed PMID: 19927746.
3. Bengtson S, Rasmussen B, Ivarsson M, Muhling J, Broman C, Marone F, *et al*. Fungus-like mycelial fossils in 2.4-billion-year-old vesicular basalt. Nat Ecol Evol. 2017;1(6):141. Epub 2017/08/16. doi: 10.1038/s41559-017-0141. PubMed PMID: 28812648.
4. Getz WM. A Biomass Flow Approach to Population Models and Food Webs. Nat Resour Model. 2012;25(1):93-121. Epub 2012/02/01. doi: 10.1111/j.1939-7445.2011.00101.x. PubMed PMID: 27688596; PubMed Central PMCID: PMCPMC5038133.
5. Schmidt AR, Dorfelt H, Perrichot V. Carnivorous fungi from Cretaceous amber. Science. 2007;318(5857):1743. Epub 2007/12/15. doi: 10.1126/science.1149947. PubMed PMID: 18079393.
6. Sung G-H, Poinar GO, Spatafora JW. The oldest fossil evidence of animal parasitism by fungi supports a Cretaceous diversification of fungal–arthropod symbioses. Molecular Phylogenetics and Evolution. 2008;49(2):495-502. doi: 10.1016/j.ympev.2008.08.028.
7. Yang E, Xu L, Yang Y, Zhang X, Xiang M, Wang C, *et al.* Origin and evolution of carnivorism in the Ascomycota (fungi). Proc Natl Acad Sci U S A. 2012;109(27):10960-5. doi: 10.1073/pnas.1120915109.
8. Liu K, Tian J, Xiang M, Liu X. How carnivorous fungi use three-celled constricting rings to trap nematodes. Protein Cell. 2012;3(5):325-8. Epub 2012/04/25. doi: 10.1007/s13238-012-2031-8. PubMed PMID: 22528749; PubMed Central PMCID: PMCPMC4875469.
9. Druzhinina IS, Seidl-Seiboth V, Herrera-Estrella A, Horwitz BA, Kenerley CM, Monte E, *et al*. *Trichoderma*: the genomics of opportunistic success. Nature Reviews Microbiology. 2011;9(10):749-59. doi: 10.1038/nrmicro2637.
10. Berini F, Caccia S, Franzetti E, Congiu T, Marinelli F, Casartelli M, *et al*. Effects of *Trichoderma viride* chitinases on the peritrophic matrix of *Lepidoptera*. Pest Manag Sci. 2016;72(5):980-9. Epub 2015/07/17. doi: 10.1002/ps.4078. PubMed PMID: 26179981.
11. Evidente A, Andolfi A, Cimmino A, Ganassi S, Altomare C, Favilla M, *et al*. Bisorbicillinoids produced by the fungus *Trichoderma citrinoviride* affect feeding preference of the aphid *Schizaphis graminum*. J Chem Ecol. 2009;35(5):533-41. Epub 2009/05/07. doi: 10.1007/s10886-009-9632-6. PubMed PMID: 19418099.
12. Ab Majid AH, Zahran Z, Abd Rahim AH, Ismail NA, Abdul Rahman W, Mohammad Zubairi KS, et al. Morphological and molecular characterization of fungus isolated from tropical bed bugs in Northern Peninsular Malaysia, *Cimex hemipterus* (Hemiptera: Cimicidae). Asian Pacific Journal of Tropical Biomedicine. 2015;5(9):707-13. doi: https://doi.org/10.1016/j.apjtb.2015.04.012.
13. Li Y, Fu K, Gao S, Wu Q, Fan L, Li Y, et al. Impact on bacterial community in midguts of the Asian corn borer larvae by transgenic *Trichoderma* strain overexpressing a heterologous *chit42* gene with chitin-binding domain. PLoS ONE. 2013;8(2):e55555. Epub 2013/03/05. doi: 10.1371/journal.pone.0055555. PubMed PMID: 23457472; PubMed Central PMCID: PMCPMC3574091.
14. Carsolio C, Gutiérrez A, Jiménez B, Montagu MV, Herrera-Estrella A. Characterization of *ech-42*, a *Trichoderma harzianum* endochitinase gene expressed during mycoparasitism. PNAS. 1994;91(23):10903-7.
15. El-Katatny MH, Somitsch W, Robra KH, El-Katatny MS, Gübitz GM. Production of chitinase and β-1,3-glucanase by *Trichoderma harzianum* for control of the phytopathogenic fungus *Sclerotium rolfsii*. Food Technology and Biotechnology. 2000;38(3):173-80.
16. Rocha-Ramírez V, Omero C, Chet I, Horwitz BA, Herrera-Estrella A. *Trichoderma atroviride* G-Protein α-Subunit Gene *tga1* Is Involved in Mycoparasitic Coiling and Conidiation. Eukaryot Cell. 2002;1(4):594-605. doi: 10.1128/EC.1.4.594-605.2002.
17. Mukherjee AK, Sampath Kumar A, Kranthi S, Mukherjee PK. Biocontrol potential of three novel *Trichoderma* strains: isolation, evaluation and formulation. 3 Biotech. 2014;4(3):275-81. Epub 2014/01/01. doi: 10.1007/s13205-013-0150-4. PubMed PMID: 28324430; PubMed Central PMCID: PMCPMC4026447.
18. Chenthamara K, Druzhinina IS. 12 Ecological Genomics of Mycotrophic Fungi. In: Druzhinina IS, Kubicek CP, editors. Environmental and Microbial Relationships. The Mycota: Springer International Publishing; 2016. p. 215-46.
19. Karlsson M, Atanasova L, Jensen DF, Zeilinger S. Necrotrophic Mycoparasites and Their Genomes. Microbiol Spectr. 2017;5(2). Epub 2017/03/11. doi: 10.1128/microbiolspec.FUNK-0016-2016. PubMed PMID: 28281442.

20. Bae H, Sicher RC, Kim MS, Kim S-H, Strem MD, Melnick RL, *et al*. The beneficial endophyte *Trichoderma hamatum* isolate DIS 219b promotes growth and delays the onset of the drought response in *Theobroma cacao*. J Exp Bot. 2009;60(11):3279-95. doi: 10.1093/jxb/erp165.
21. Bailey BA, Strem MD, Wood D. *Trichoderma* species form endophytic associations within *Theobroma cacao* trichomes. Mycol Res. 2009;113(Pt 12):1365-76. Epub 2009/09/22. doi: 10.1016/j.mycres.2009.09.004. PubMed PMID: 19765658.
22. Bae H, Roberts DP, Lim HS, Strem MD, Park SC, Ryu CM, *et al*. Endophytic *Trichoderma* isolates from tropical environments delay disease onset and induce resistance against *Phytophthora capsici* in hot pepper using multiple mechanisms. Mol Plant Microbe Interact. 2011;24(3):336-51. Epub 2010/11/26. doi: 10.1094/MPMI-09-10-0221. PubMed PMID: 21091159.
23. Rosmana A, Samuels GJ, Ismaiel A, Ibrahim ES, Chaverri P, Herawati Y, *et al*. *Trichoderma asperellum*: A Dominant Endophyte Species in Cacao Grown in Sulawesi with Potential for Controlling Vascular Streak Dieback Disease. Trop plant pathol. 2015;40(1):19-25. doi: 10.1007/s40858-015-0004-1.
24. Rosmana A, Nasaruddin N, Hendarto H, Hakkar AA, Agriansyah N. Endophytic Association of *Trichoderma asperellum* within *Theobroma cacao* Suppresses Vascular Streak Dieback Incidence and Promotes Side Graft Growth. Mycobiology. 2016;44(3):180-6. Epub 2016/10/30. doi: 10.5941/MYCO.2016.44.3.180. PubMed PMID: 27790069; PubMed Central PMCID: PMCPMC5078131.
25. Chen JL, Sun SZ, Miao CP, Wu K, Chen YW, Xu LH, et al. Endophytic *Trichoderma gamsii* YIM PH30019: a promising biocontrol agent with hyperosmolar, mycoparasitism, and antagonistic activities of induced volatile organic compounds on root-rot pathogenic fungi of Panax notoginseng. J Ginseng Res. 2016;40(4):315-24. Epub 2016/10/18. doi: 10.1016/j.jgr.2015.09.006. PubMed PMID: 27746683; PubMed Central PMCID: PMCPMC5052430.
26. Li Destri Nicosia MG, Mosca S, Mercurio R, Schena L. Dieback of *Pinus nigra* Seedlings Caused by a Strain of *Trichoderma viride*. Plant Disease. 2014;99(1):44-9. doi: 10.1094/PDIS-04-14-0433-RE.
27. Gautheret A, Dromer F, Bourhis JH, Andremont A. *Trichoderma pseudokoningii* as a cause of fatal infection in a bone marrow transplant recipient. Clin Infect Dis. 1995;20(4):1063-4. Epub 1995/04/01. PubMed PMID: 7795053.
28. Furukawa H, Kusne S, Sutton DA, Manez R, Carrau R, Nichols L, *et al*. Acute invasive sinusitis due to *Trichoderma longibrachiatum* in a liver and small bowel transplant recipient. Clin Infect Dis. 1998;26(2):487-9. Epub 1998/03/21. PubMed PMID: 9502475.
29. Sahebani N, Hadavi N. Biological control of the root-knot nematode *Meloidogyne javanica* by *Trichoderma harzianum.* Soil Biology and Biochemistry. 2008;40(8):2016-20. doi: 10.1094/PHYTO.2001.91.7.687. PubMed PMID: 18942999.
30. Zhang S, Gan Y, Ji W, Xu B, Hou B, Liu J. Mechanisms and Characterization of T*richoderma longibrachiatum* T6 in Suppressing Nematodes (*Heterodera avenae*) in Wheat. Front Plant Sci. 2017;8:1491. Epub 2017/10/03. doi: 10.3389/fpls.2017.01491. PubMed PMID: 28966623; PubMed Central PMCID: PMCPMC5605630.
31. de Souza Maia Filho F, da Silva Fonseca AO, Persici BM, de Souza Silveira J, Braga CQ, Potter L, *et al*. *Trichoderma virens* as a biocontrol of *Toxocara canis*: In vivo evaluation. Rev Iberoam Micol. 2017;34(1):32-5. Epub 2017/01/23. doi: 10.1016/j.riam.2016.06.004. PubMed PMID: 28109772.
32. Ghosh SK, Pal S. Entomopathogenic potential of *Trichoderma longibrachiatum* and its comparative evaluation with malathion against the insect pest *Leucinodes orbonalis*. Environ Monit Assess. 2016;188(1):37. Epub 2015/12/18. doi: 10.1007/s10661-015-5053-x. PubMed PMID: 26676413.
33. Pacheco JC, Poltronieri AS, Porsani MV, Zawadneak MAC, Pimentel IC. Entomopathogenic potential of fungi isolated from intertidal environments against the cabbage aphid *Brevicoryne brassicae* (Hemiptera: aphididae). Biocontrol Science and Technology. 2017;27(4):496-509. doi: 10.1080/09583157.2017.1315053.
34. Zahran Z, Mohamed Nor NMI, Dieng H, Satho T, Ab Majid AH. Laboratory efficacy of mycoparasitic fungi (*Aspergillus tubingensis* and *Trichoderma harzianum*) against tropical bed bugs (*Cimex hemipterus*) (Hemiptera: Cimicidae). Asian Pacific Journal of Tropical Biomedicine. 2017;7(4):288-93. doi: https://doi.org/10.1016/j.apjtb.2016.12.021.
35. Li YY, Tang J, Fu KH, Gao SG, Wu Q, Chen J. Construction of transgenic *Trichoderma koningi* with *chit42* of *Metarhizium anisopliae* and analysis of its activity against the Asian corn

borer. J Environ Sci Health B. 2012;47(7):622-30. Epub 2012/05/09. doi: 10.1080/03601234.2012.668455. PubMed PMID: 22560024.
36. Cianchetta S, Galletti S, Burzi PL, Cerato C. Hydrolytic potential of *Trichoderma* sp. strains evaluated by microplate-based screening followed by switchgrass saccharification. Enzyme Microb Technol. 2012;50(6-7):304-10. Epub 2012/04/17. doi: 10.1016/j.enzmictec.2012.02.005. PubMed PMID: 22500897.
37. Bischof RH, Ramoni J, Seiboth B. Cellulases and beyond: the first 70 years of the enzyme producer *Trichoderma reesei*. Microb Cell Fact. 2016;15. doi: 10.1186/s12934-016-0507-6.
38. Keshavarz B, Khalesi M. *Trichoderma reesei*, a superior cellulase source for industrial applications. Biofuels. 2016;7(6):713-21. doi: 10.1080/17597269.2016.1192444.
