# Supplementary material for: Massive lateral transfer of genes encoding plant cell wall-degrading enzymes to the mycoparasitic fungus Trichoderma from its plant-associated hosts
Source: PLoS Genet. 2018 Apr 9;14(4):e1007322. doi: 10.1371/journal.pgen.1007322 (PMC5908196; doi:10.1371/journal.pgen.1007322)
Supplement: S1 Fig — Strains were evaluated after 10 days of incubation at 28°C in darkness. Yellow, green and white shape outlines correspond to good, weak and no growth, respectively. Data are representatives of four separate experiments. (PDF) [file pgen.1007322.s009.pdf]

S1 Figure: Growth of *Trichoderma* spp., *Escovopsis weberi* and *Pestalotiopsis fici* on natural substrates resembling polymers in the fungal and plant cell walls.

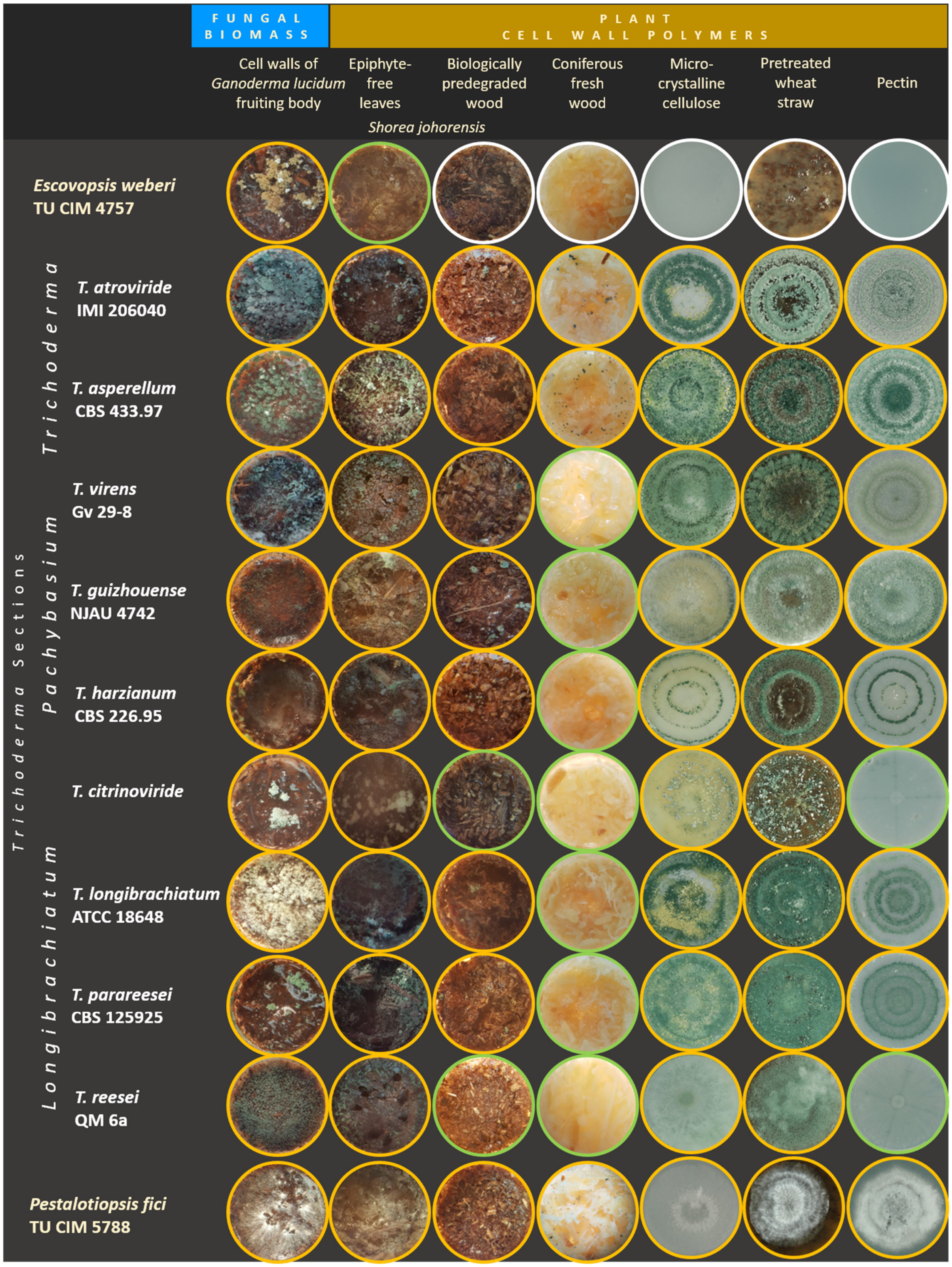

Strains were evaluated after 10 days of incubation at 28˚C in darkness. Yellow, green and white shape outlines correspond to good, weak and no growth, respectively. The first four assays were done in 24-well plates. The last three columns show assays in Petri plates, 9 cm in diameter. Data are representatives of four separate experiments.
