# Supplementary material for: Massive lateral transfer of genes encoding plant cell wall-degrading enzymes to the mycoparasitic fungus Trichoderma from its plant-associated hosts
Source: PLoS Genet. 2018 Apr 9;14(4):e1007322. doi: 10.1371/journal.pgen.1007322 (PMC5908196; doi:10.1371/journal.pgen.1007322)
Supplement: S2 Fig — (PDF) [file pgen.1007322.s010.pdf]

Supporting Information S2 Figure

COMPLETE PHYLOME OF *TRICHODERMA* CARBOHYDRATE ACTIVE ENZYMES INVOLVED IN DEGRADATION OF PLANT CELL WALL (pcwdCAZome) AND RESPECTIVE REGULATORY PROTEINS

Druzhinina *et al*.

pcwdCAZymes 4

GH6 EC 3.2.1.91 Cellobiohydrolase CEL6 4

GH7 EC 3.2.1.4 Endo-ß-1,4-glucanase CEL7B 5

GH7 EC 3.2.1.91 Cellobiohydrolase CEL7A 6

GH5 EC 3.2.1.4 Endo-ß-1,4-glucanase Clade A 7

GH5 EC 3.2.1.4 Endo-ß-1,4-glucanase Clade B 8

GH5 EC 3.2.1.4 Endo-ß-1,4-glucanase Clade C 9

GH12 EC 3.2.1.4 Endo-ß-1,4-glucanase Clade A 10

GH12 EC 3.2.1.4 Endo-ß-1,4-glucanase Clade B 11

GH45 EC 3.2.1.4 Endo-ß-1,4-glucanase 12

GH1 EC 3.2.1.21 ß-1,4-glucosidase Clade A 13

GH1 EC 3.2.1.21 ß-1,4-glucosidase Clade B 14

GH1 EC 3.2.1.21 ß-1,4-glucosidase Clade C 15

GH3 EC 3.2.1.21 ß-1,4-glucosidase 16

GH10 EC 3.2.1.8 Endo-ß-1,4-xylanase 18

GH11 EC 3.2.1.8 Endo-ß-1,4-xylanase 19

GH30 EC 3.2.1.8 Endo-ß-1,4-xylanase Clade A 20

GH30 EC 3.2.1.8 Endo-ß-1,4-xylanase Clade B 21

GH3 EC 3.2.1.37 Xylan-1,4-ß-xylosidase 22

GH74 EC3.2.1.151 Xyloglucanase 23

GH5 EC 3.2.1.78 Endo-ß-1,4-mannanase 24

GH26 EC 3.2.1.78 Endo-ß-1,4-mannanase 25

GH27 EC 3.2.1.22 α-1,4-galactosidase 26

GH36 EC 3.2.1.22 α-1,4-galactosidases clade A 28

GH36 EC 3.2.1.22 α-1,4-galactosidases clade B 29

GH43 EC 3.2.1.55, EC 3.2.1.37 α-L-arabinofuranosidase and ß-xylosidase 30

GH51 EC 3.2.1.55 α-L-arabinofuranosidase 31

GH54 EC 3.2.1.55 α-L-arabinofuranosidase 32

GH62 **EC 3.2.1.55 α-L-arabinofuranosidase** **33**

GH67 **EC 3.2.1.131 (xylan) α-1,2-glucuronidase** **34**

GH115 **EC 3.2.1.- α-(4-O-methyl)-glucuronidase** **35**

GH95 **EC 3.2.1.51 α-L-fucosidase** **36**

GH28 **EC 3.2.1.67, EC 3.2.1.171 Exo-polygalacturonase and rhamnogalacturonase** **37**

GH28 **EC 3.2.1.15 Polygalacturonase** **38**

GH28 **EC 3.2.1.- Exo-xylogalacturan hydrolase** **39**

GH78 **EC 3.2.1.40 α-L-rhamnosidase** **40**

PL1 **EC 4.2.2.2 Pectate lyase** **41**

GH35 **EC 3.2.1.23 ß-galactosidase** **42**

GH79 **EC 3.2.1.31 β-(4-O-methyl)-glucuronidase** **43**

GH88 **EC 3.2.1 Δ-4,5-unsaturated β-glucuronyl hydrolase** **44**

GH105 **EC 3.2.1.172 Unsaturated rhamnogalacturonyl hydrolase** **45**

GH121 **EC 3.2.1.- ß-L-arabinobiosidase** **46**

GH127 **EC 3.2.1.185 β-L-arabinofuranosidase Clade A** **47**

GH127 **EC 3.2.1.185 β-L-arabinofuranosidase Clade B** **48**

AA9 (formerly "GH61") Copper-dependent lytic polysaccharide monooxygenase **49**

Swollenin **51**

PCWDCAZYME REGULATORY PROTEINS 52

XYR1 **52**

ACE2 **53**

ACE3 **54**

pcwdCAZymes

GH6 EC 3.2.1.91 Cellobiohydrolase CEL6

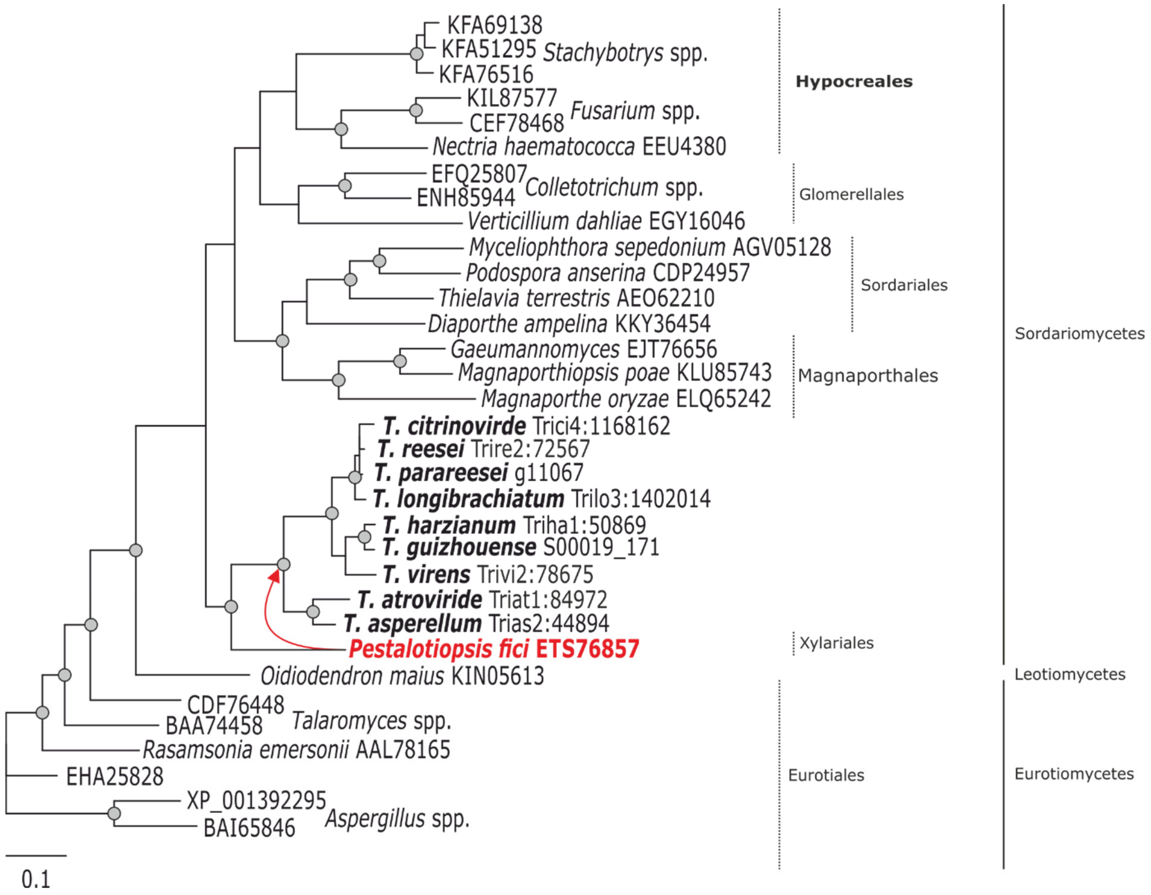

Phylogram based on Dayhoff amino acid substitution model using an alignment containing 20707 characters. Bayesian analysis was run for 1 million mcmc generations and a strict consensus tree was obtained by summarizing 7500 trees, after burning first 25% of obtained 10,000 trees. Mean tree length and variance are 7.269450E+00 and 3.9080650E-02, respectively. Posterior probabilities more than 95% are marked with circular nodes. Dashed vertical bars and non-dashed vertical bars represents the taxonomic order and taxonomic class in the phylum Ascomycota, respectively. LGT inferred by NOTUNG and Phylogeny is shown with a red arrow to the node and the respective donor is marked in red.

GH7 EC 3.2.1.4 Endo-ß-1,4-glucanase CEL7B

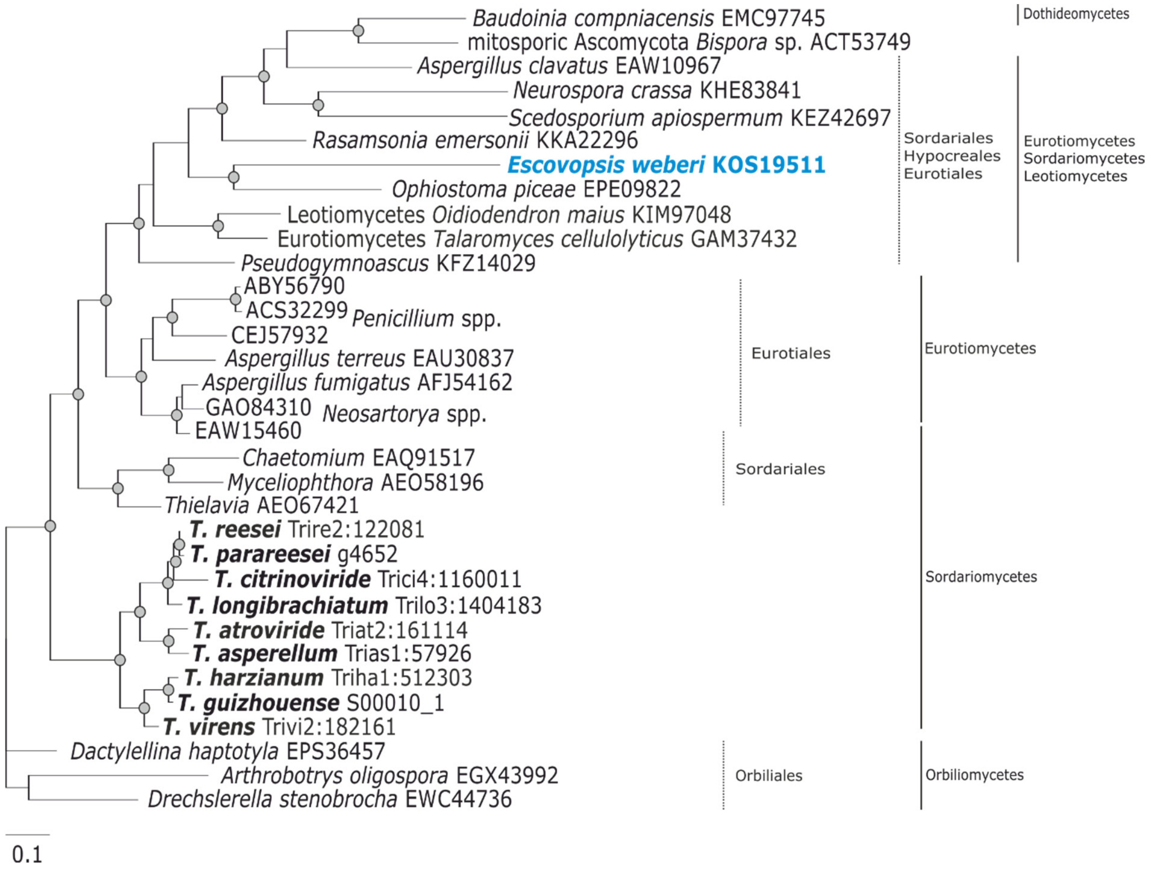

Phylogram based on Dayhoff amino acid substitution model using an alignment containing 18546 characters. Bayesian analysis was run for 1 million mcmc generations and a strict consensus tree was obtained by summarizing 7500 trees, after burning first 25% of obtained 10,000 trees. Mean tree length and variance are 7.457580E+00 and 2.1536720E-02, respectively. Posterior probabilities more than 95% are marked with circular nodes. Dashed vertical bars and non-dashed vertical bars represents the taxonomic order and taxonomic class in the phylum Ascomycota, respectively

GH7 EC 3.2.1.91 Cellobiohydrolase CEL7A

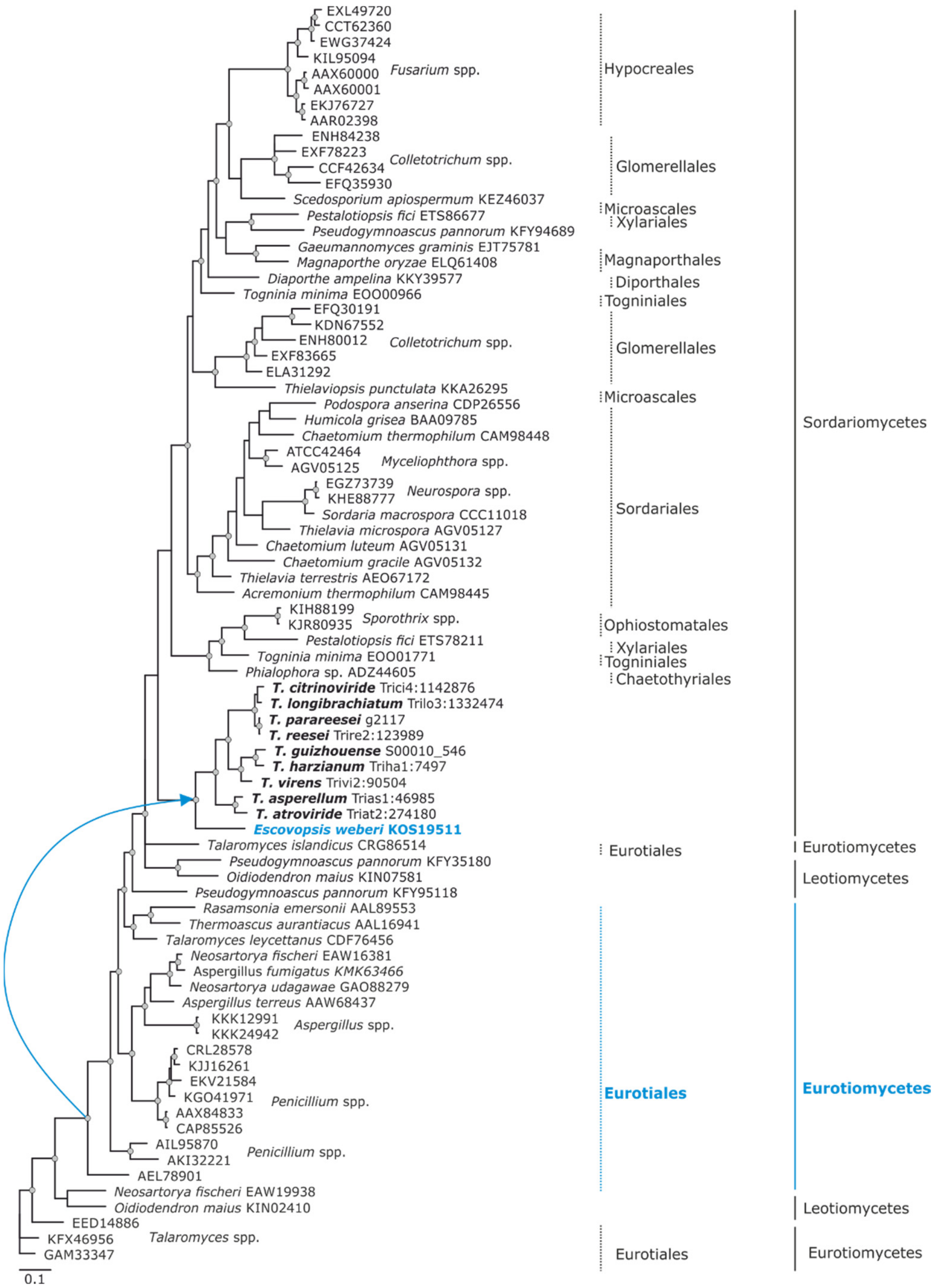

Phylogram based on Dayhoff amino acid substitution model using an alignment containing 38720 characters. Bayesian analysis was run for 1 million mcmc generations and a strict consensus tree was obtained by summarizing 7500 trees, after burning first 25% of obtained 10,000 trees. Mean tree length and variance are 9.480271E+00 and 2.2693320E-02, respectively. Posterior probabilities more than 95% are marked with circular nodes. Dashed vertical bars and non-dashed vertical bars represents the taxonomic order and taxonomic class in the phylum Ascomycota, respectively. LGT inferred by phylogeny and NOTUNG is shown with a blue arrow since it is occuring before *Trichoderma* and *Escovopsis* diverged and the respective donors are marked in blue.

GH5 EC 3.2.1.4 Endo-ß-1,4-glucanase Clade A

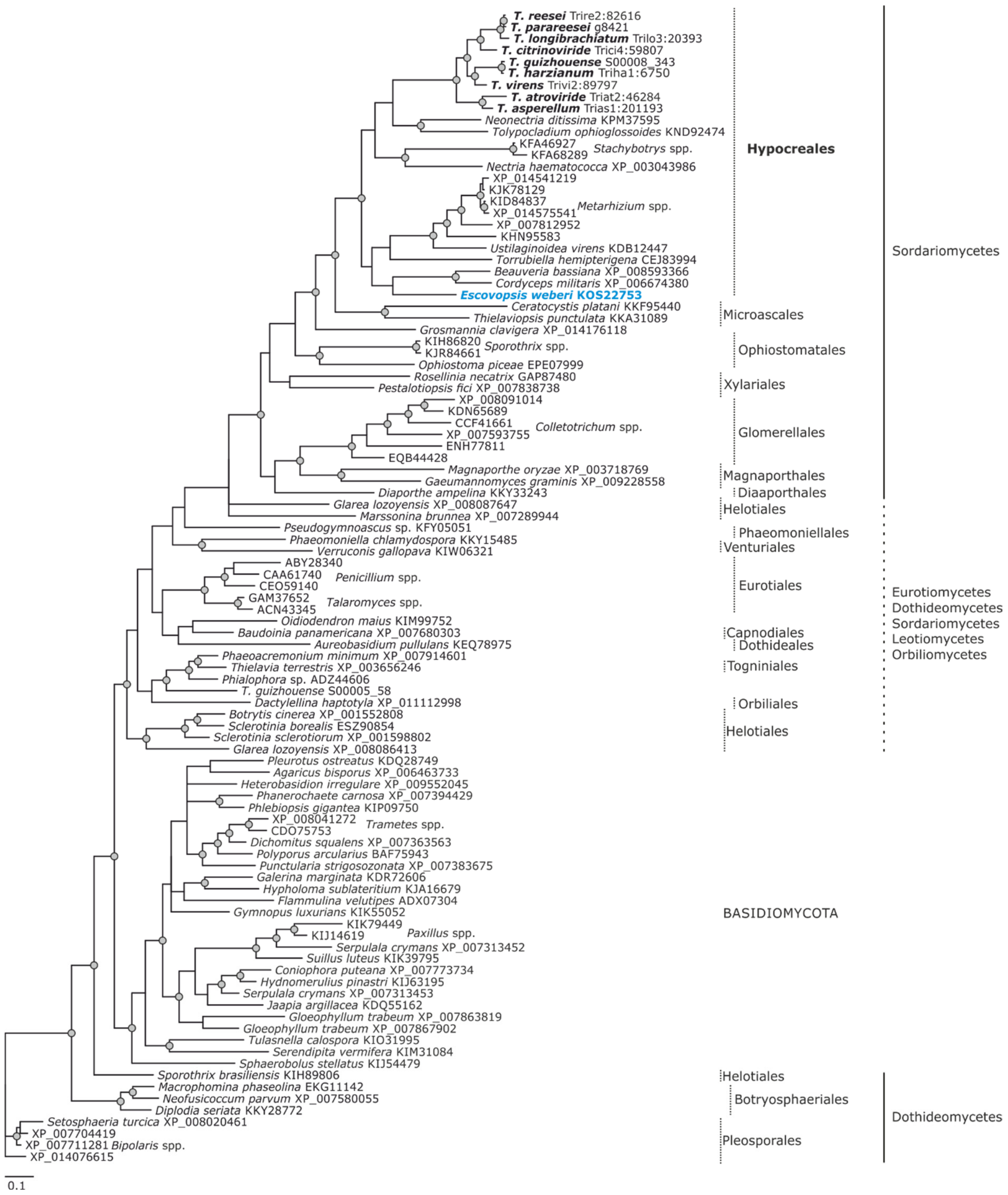

Phylogram based on Dayhoff amino acid substitution model using an alignment containing 70686 characters. Bayesian analysis was run for 1 million mcmc generations and a strict consensus tree was obtained by summarizing 7500 trees, after burning first 25% of obtained 10,000 trees. Mean tree length and variance are 2.425237E+01 and 7.0550690E-02, respectively. Posterior probabilities more than 95% are marked with circular nodes. Dashed vertical bars and non-dashed vertical bars represents the taxonomic order and taxonomic class in the phylum Ascomycota, respectively.

GH5 EC 3.2.1.4 Endo-ß-1,4-glucanase Clade B

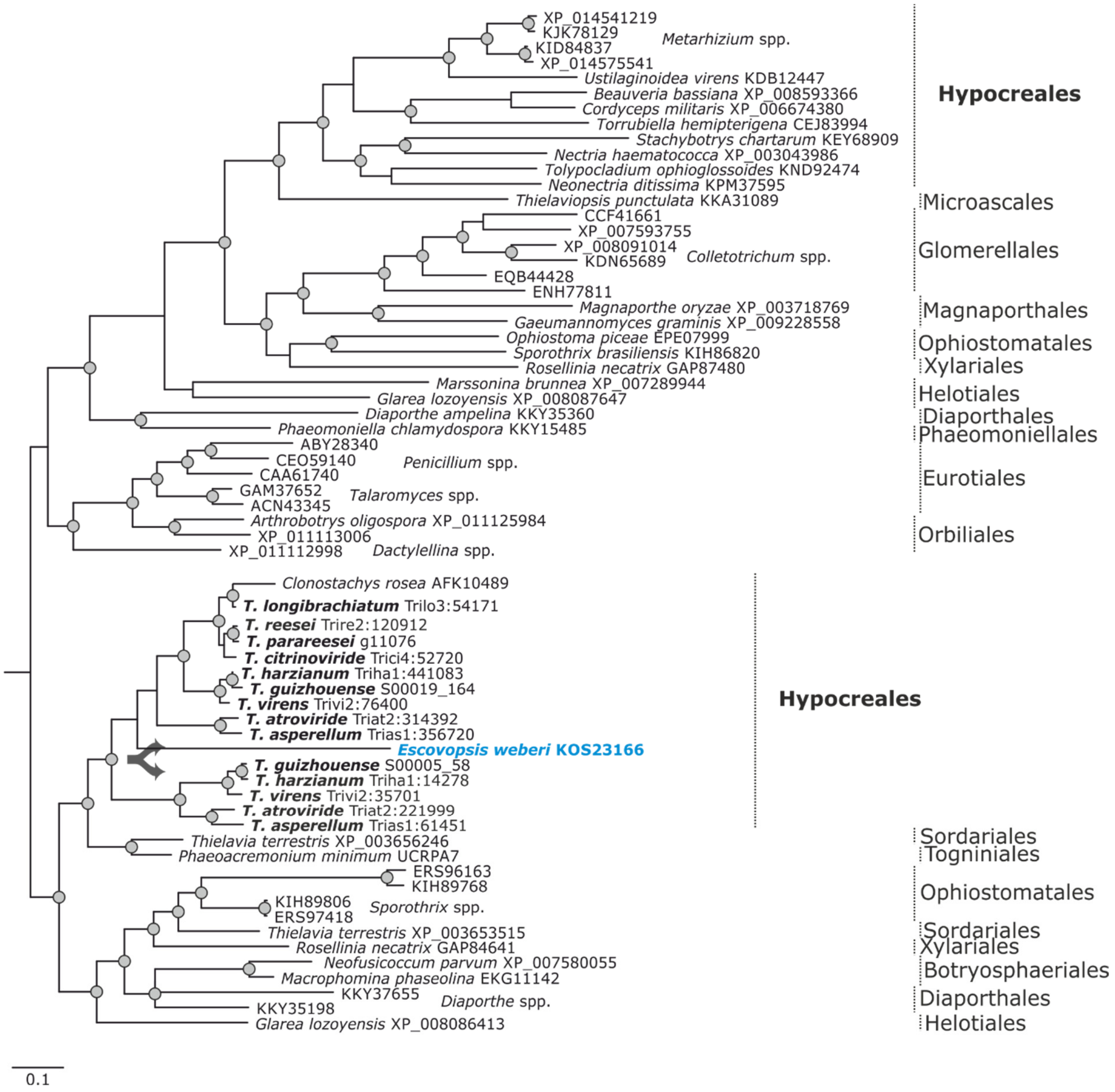

Phylogram based on Dayhoff amino acid substitution model using an alignment containing 116172 characters. Bayesian analysis was run for 10 million mcmc generations and a strict consensus tree was obtained by summarizing 75000 trees, after burning first 25% of obtained 100,000 trees. Mean tree length and variance are 3.029779E+01 and 8.9287420E-02, respectively. Posterior probabilities more than 95% are marked with circular nodes. Dashed vertical bars and non-dashed vertical bars represents the taxonomic order and taxonomic class in the phylum Ascomycota, respectively. The bifurcated arrow indicates duplication as inferred by NOTUNG.

GH5 EC 3.2.1.4 Endo-ß-1,4-glucanase Clade C

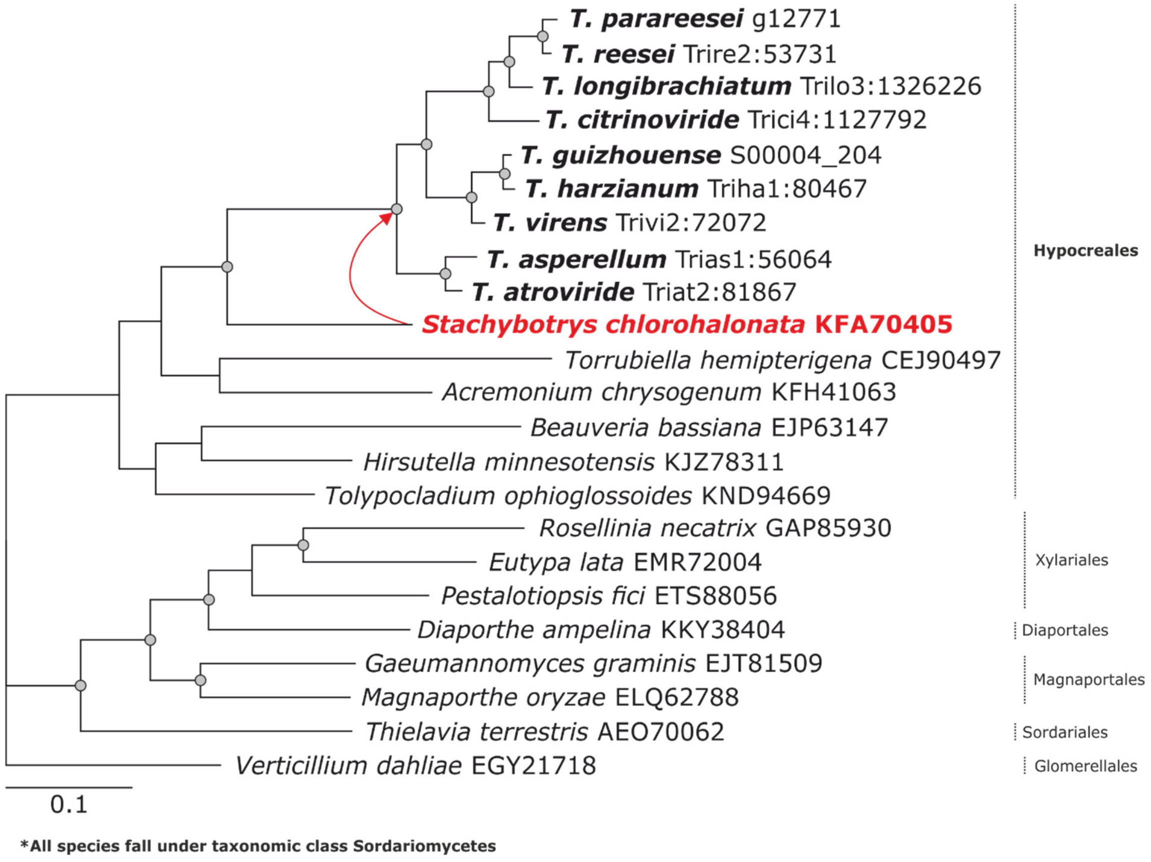

Phylogram based on Dayhoff amino acid substitution model using an alignment containing 9867 characters. Bayesian analysis was run for 1 million mcmc generations and a strict consensus tree was obtained by summarizing 7500 trees, after burning first 25% of obtained 10,000 trees. Mean tree length and variance are 3.404872E+00 and 9.6194180E-03, respectively. Posterior probabilities more than 95% are marked with circular nodes. Dashed vertical bars represents the taxonomic order. LGT inferred by Phylogeny and NOTUNG is shown with a red arrow to the node and the respective donor is marked in red.

GH12 EC 3.2.1.4 Endo-ß-1,4-glucanase Clade A

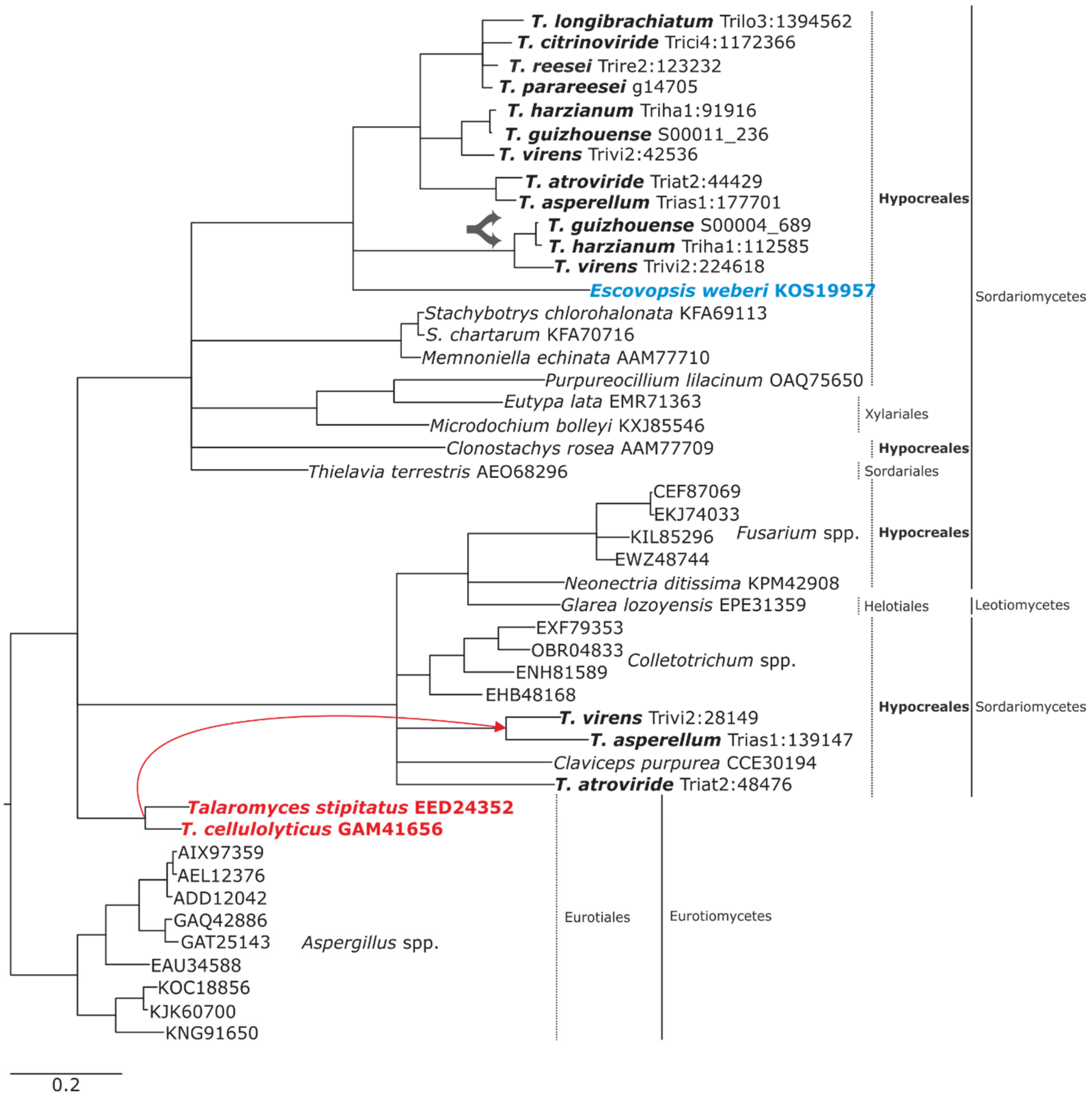

Phylogram based on Dayhoff amino acid substitution model using an alignment containing 11638 characters. Bayesian analysis was run for 1 million mcmc generations and a strict consensus tree was obtained by summarizing 7500 trees, after burning first 25% of obtained 10,000 trees. Mean tree length and variance are 7.751170E+00 and 3.915011e-002, respectively. Clades with Posterior probabilities less than 95% are collapsed. Dashed vertical bars and non-dashed vertical bars represents the taxonomic order and taxonomic class in the phylum Ascomycota, respectively. LGT inferred by Phylogeny and NOTUNG is shown with a red arrow to the node and the respective donor is marked in red. The bifurcated arrow indicates duplication as inferred by NOTUNG.

GH12 EC 3.2.1.4 Endo-ß-1,4-glucanase Clade B

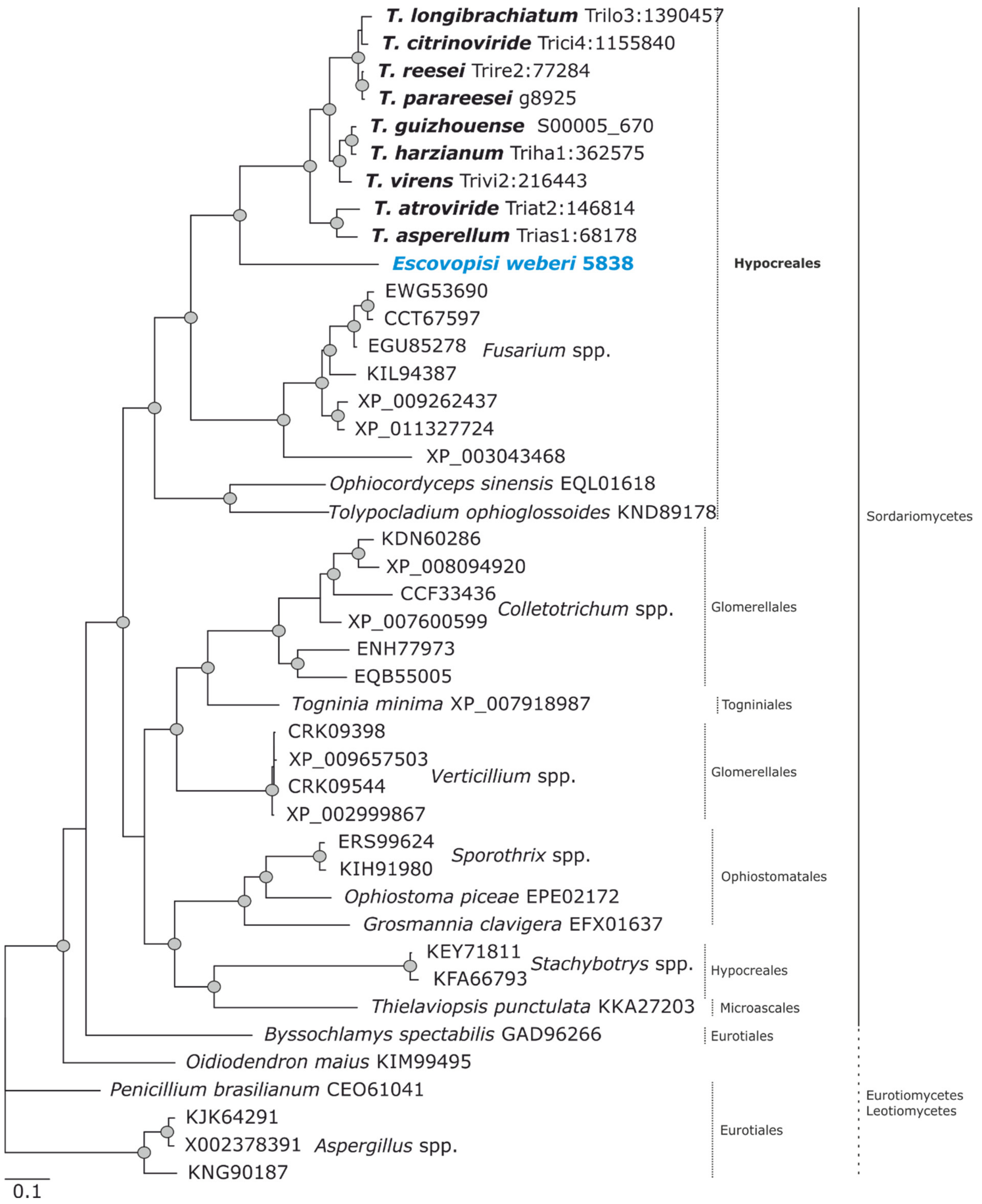

Phylogram based on Dayhoff amino acid substitution model using an alignment containing 15910 characters. Bayesian analysis was run for 1 million mcmc generations and a strict consensus tree was obtained by summarizing 7500 trees, after burning first 25% of obtained 10,000 trees. Mean tree length and variance are 7.371628E+00 and 2.7724420E-02, respectively. Posterior probabilities more than 95% are marked with circular nodes. Dashed vertical bars and non-dashed vertical bars represents the taxonomic order and taxonomic class in the phylum Ascomycota, respectively.

GH45 EC 3.2.1.4 Endo-ß-1,4-glucanase

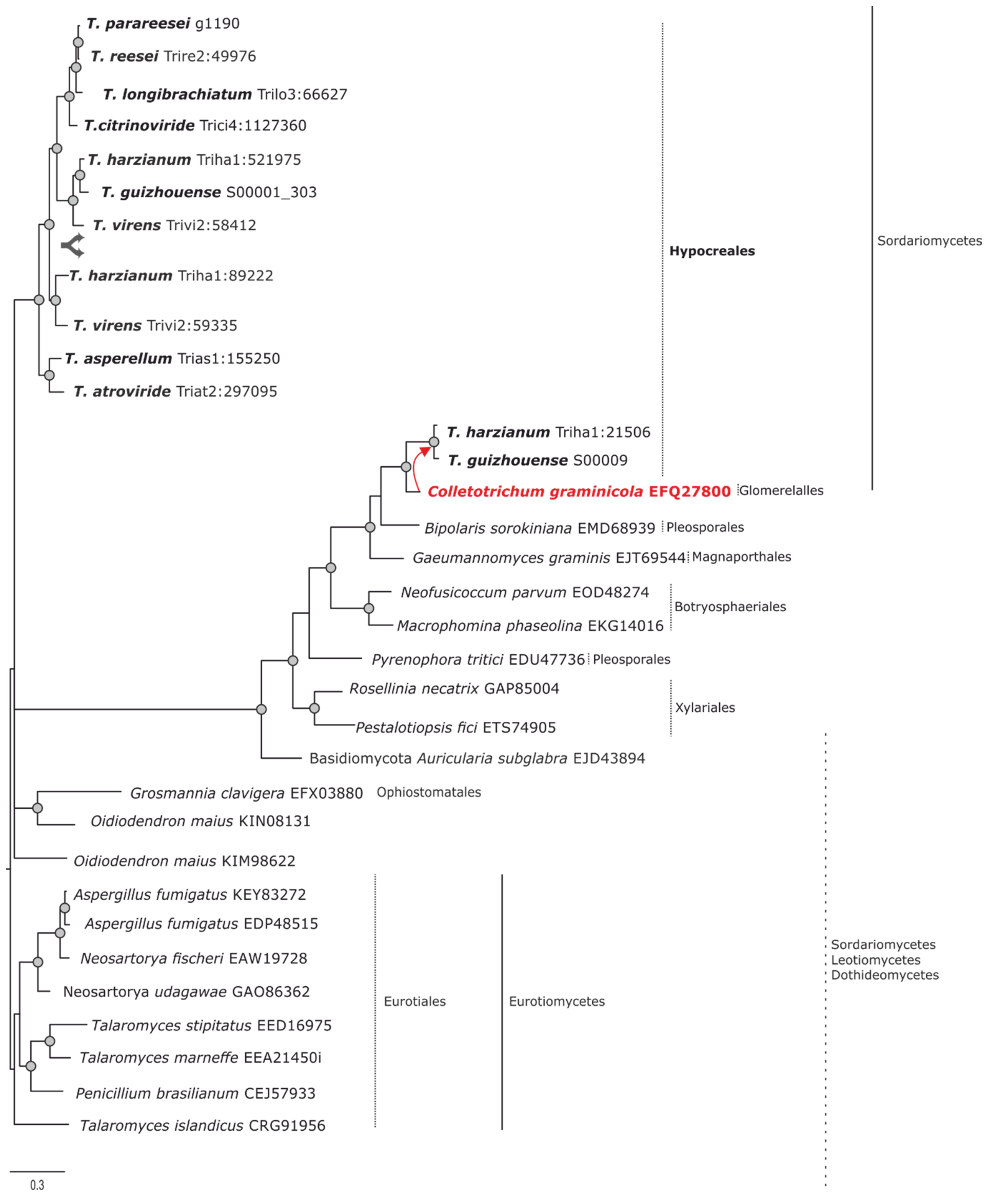

Phylogram based on Dayhoff amino acid substitution model using an alignment containing 11220 characters. Bayesian analysis was run for 1 million mcmc generations and a strict consensus tree was obtained by summarizing 7500 trees, after burning first 25% of obtained 10,000 trees. Mean tree length and variance are 8.157591E+00 and 5.7262160E-02, respectively. Posterior probabilities more than 95% are marked with circular nodes. Dashed vertical bars and non-dashed vertical bars represents the taxonomic order and taxonomic class in the phylum Ascomycota, respectively. LGT inferred by Phylogeny and NOTUNG is shown with a red arrow to the node and the respective donor is marked in red. The bifurcated arrow indicates duplication as inferred by NOTUNG.

GH1 EC 3.2.1.21 ß-1,4-glucosidase Clade A

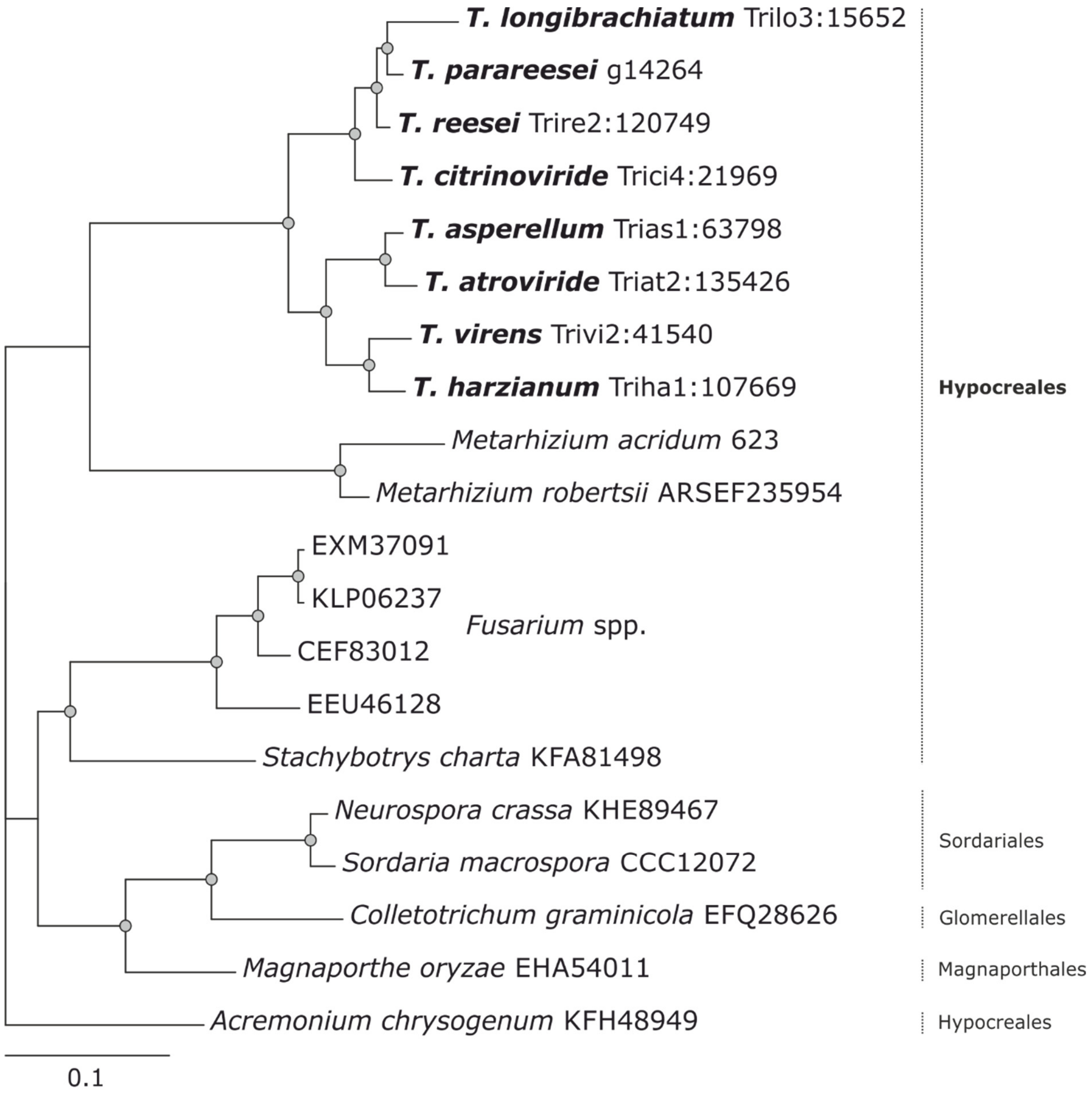

***All species fall under taxonomic class Sordariomycetes**

Phylogram based on Dayhoff amino acid substitution model using an alignment containing 9620 characters. Bayesian analysis was run for 1 million mcmc generations and a strict consensus tree was obtained by summarizing 7500 trees, after burning first 25% of obtained 10,000 trees. Mean tree length and variance are 1.568965E+00 and 3.6838590E-03, respectively. Posterior probabilities more than 95% are marked with circular nodes. Dashed vertical bars represents the taxonomic order.

GH1 EC 3.2.1.21 ß-1,4-glucosidase Clade B

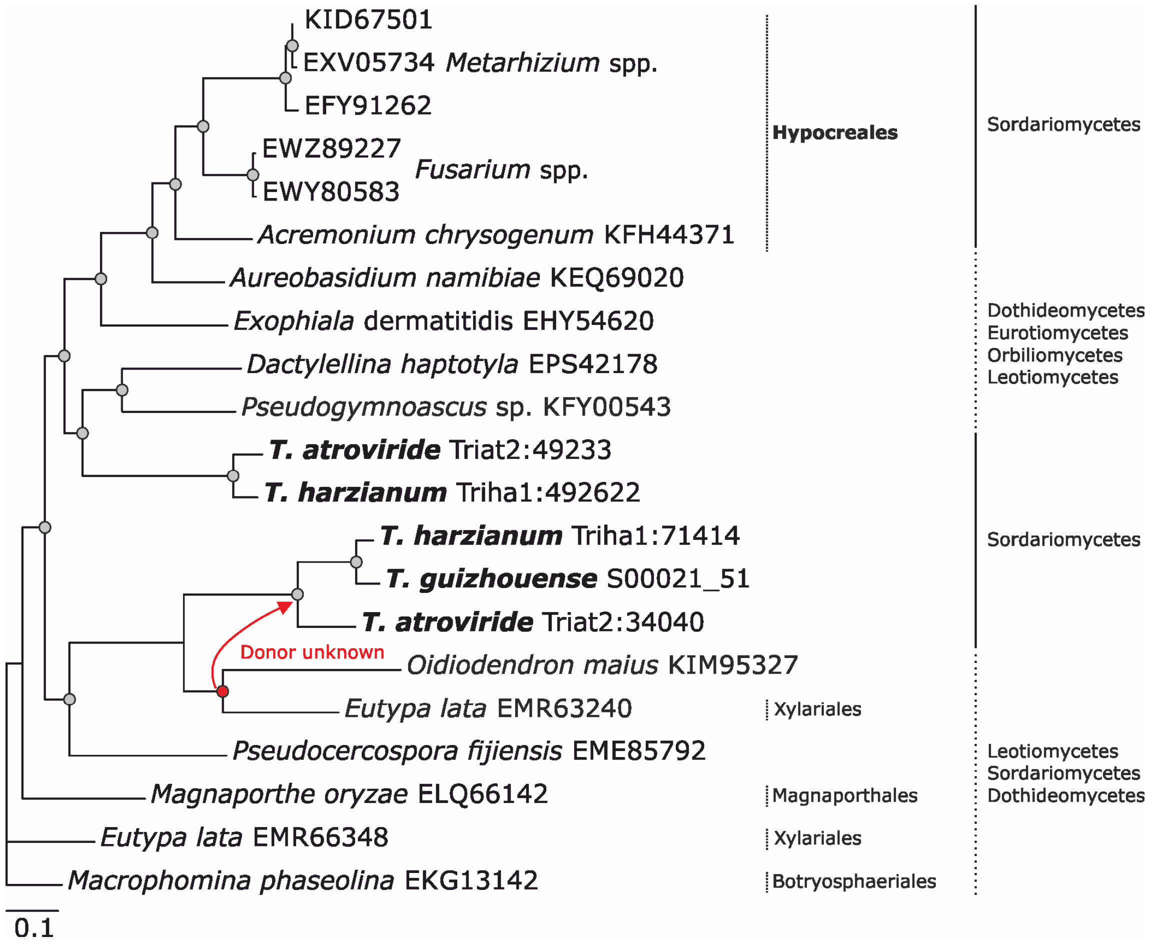

Phylogram based on Dayhoff amino acid substitution model using an alignment containing 14007 characters. Bayesian analysis was run for 1 million mcmc generations and a strict consensus tree was obtained by summarizing 7500 trees, after burning first 25% of obtained 10,000 trees. Mean tree length and variance are 4.467266E+00 and 8.8859970E-03, respectively. Posterior probabilities more than 95% are marked with circular nodes. Dashed vertical bars and non-dashed vertical bars represents the taxonomic order and taxonomic class in the phylum Ascomycota, respectively. LGT inferred by Phylogeny and NOTUNG is shown with a red arrow to the node and the respective donor is marked in red.

GH1 EC 3.2.1.21 ß-1,4-glucosidase Clade C

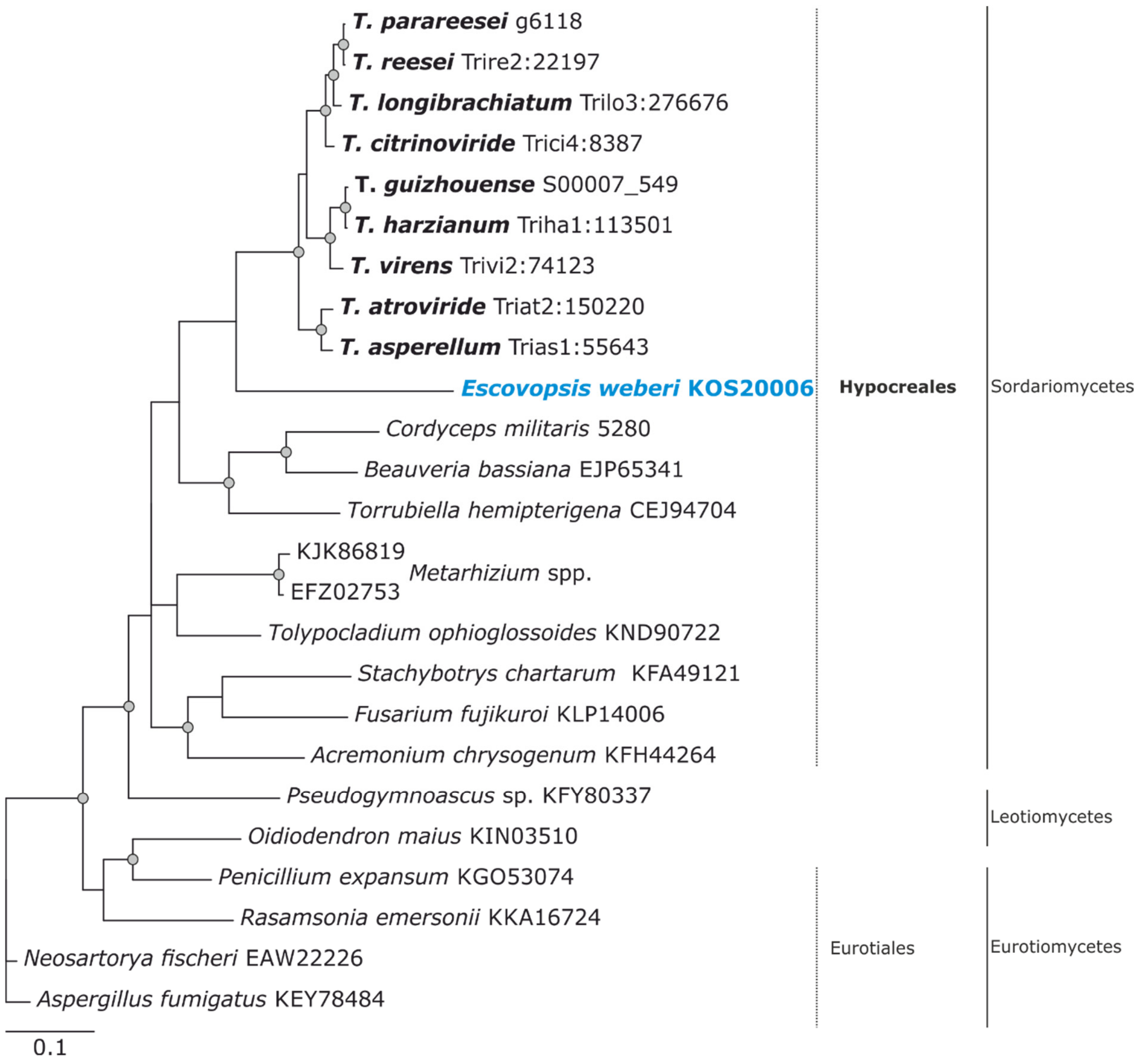

Phylogram based on Dayhoff amino acid substitution model using an alignment containing 12150 characters. Bayesian analysis was run for 1 million mcmc generations and a strict consensus tree was obtained by summarizing 7500 trees, after burning first 25% of obtained 10,000 trees. Mean tree length and variance are 2.625839E+00 and 6.1282930E-03, respectively. Posterior probabilities more than 95% are marked with circular nodes. Dashed vertical bars and non-dashed vertical bars represents the taxonomic order and taxonomic class in the phylum Ascomycota, respectively.

GH3 EC 3.2.1.21 ß-1,4-glucosidase

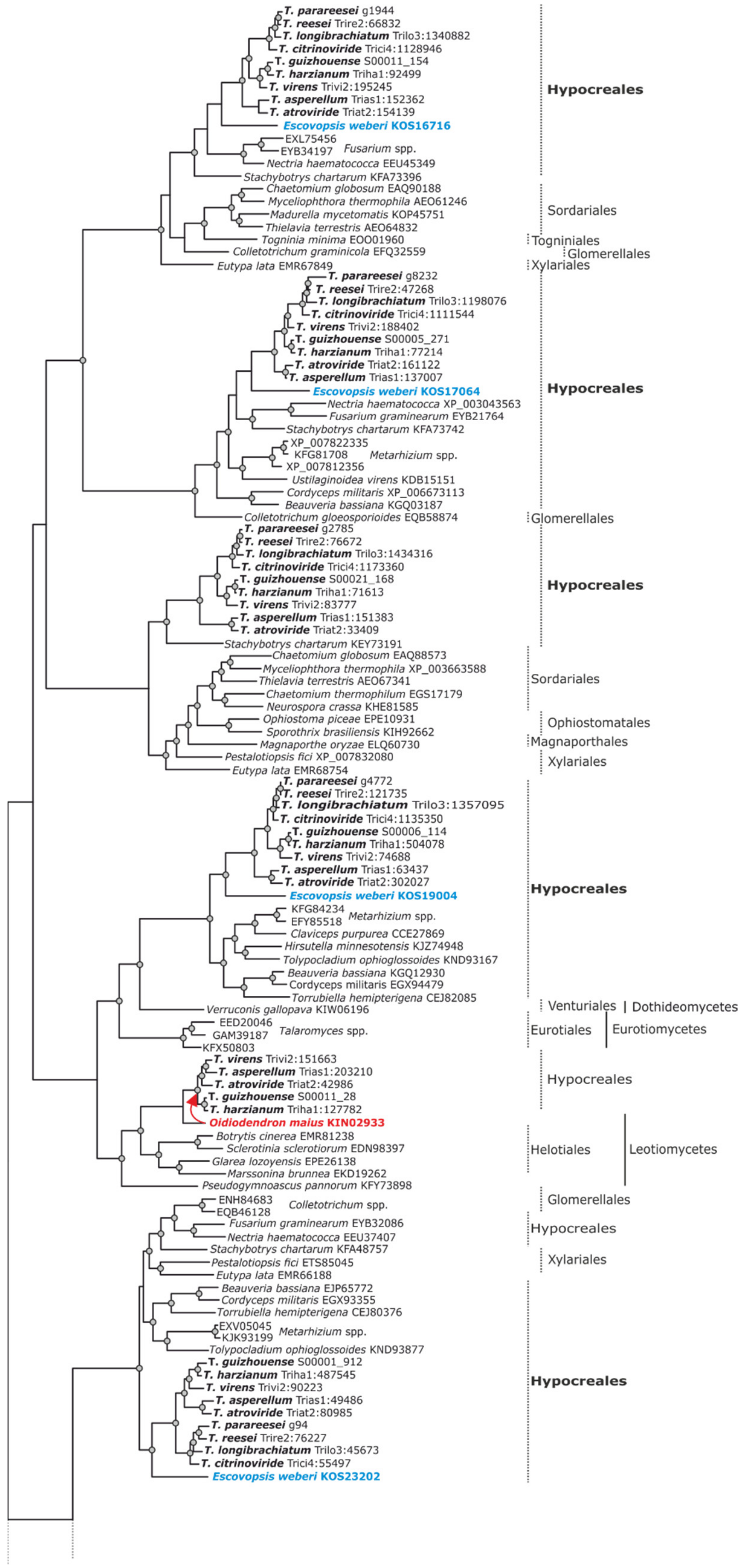

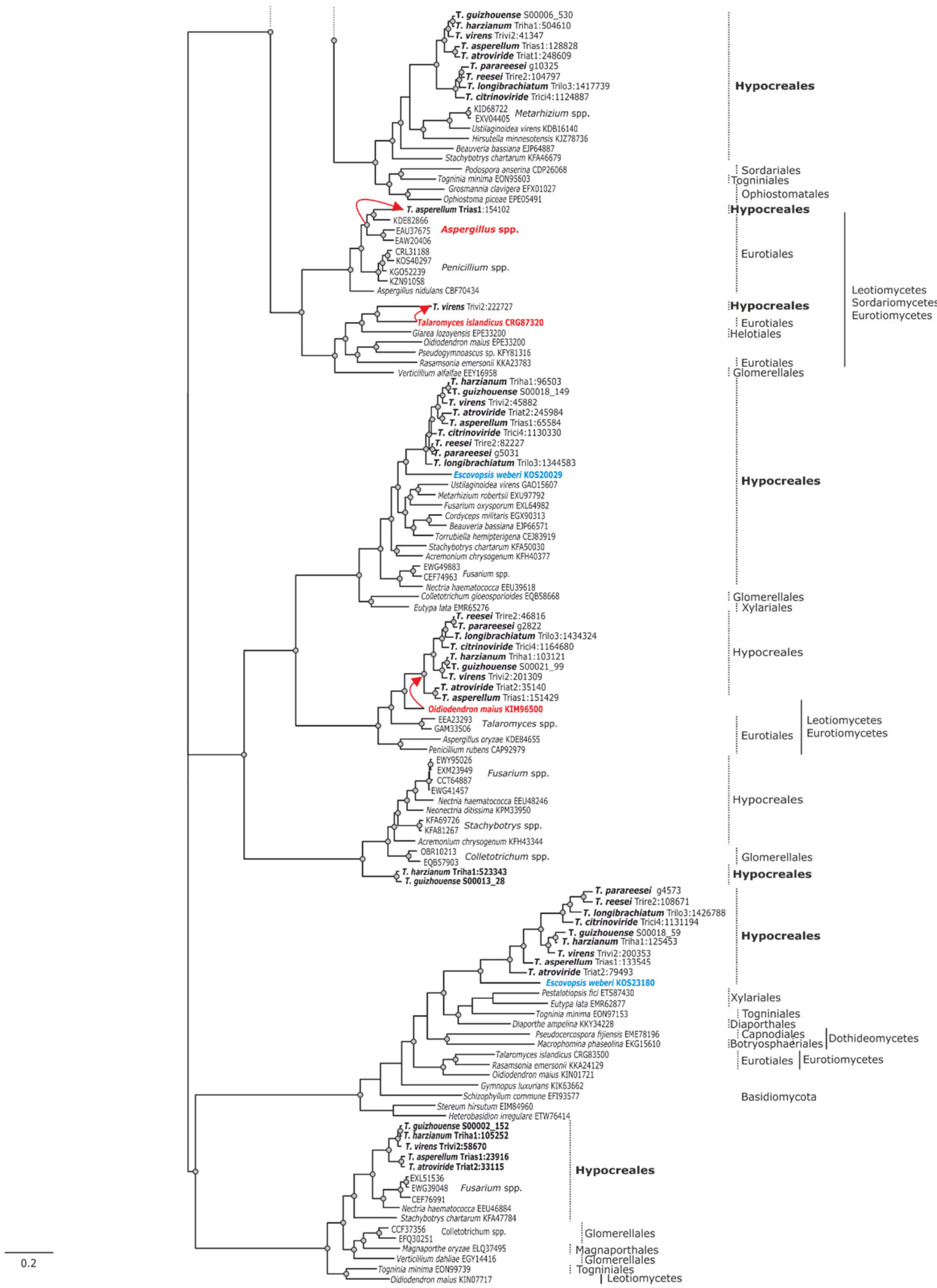

Phylogram based on Dayhoff amino acid substitution model using an alignment containing 209655 characters. Bayesian analysis was run for 3 million mcmc generations and a strict consensus tree was obtained by summarizing 22500 trees, after burning first 25% of obtained 100,000 trees. Mean tree length and variance are 2.077053E+01 and 3.2554480E-02, respectively. Posterior probabilities more than 95% are marked with circular nodes. Dashed vertical bars and non-dashed vertical bars represents the taxonomic order and taxonomic class in the phylum Ascomycota, respectively. All non-labelled taxonomic classes are from class Sordariomycetes. LGT inferred by Phylogeny and NOTUNG is shown with a red arrow to the node and the respective donor is marked in red.

GH10 EC 3.2.1.8 Endo-ß-1,4-xylanase

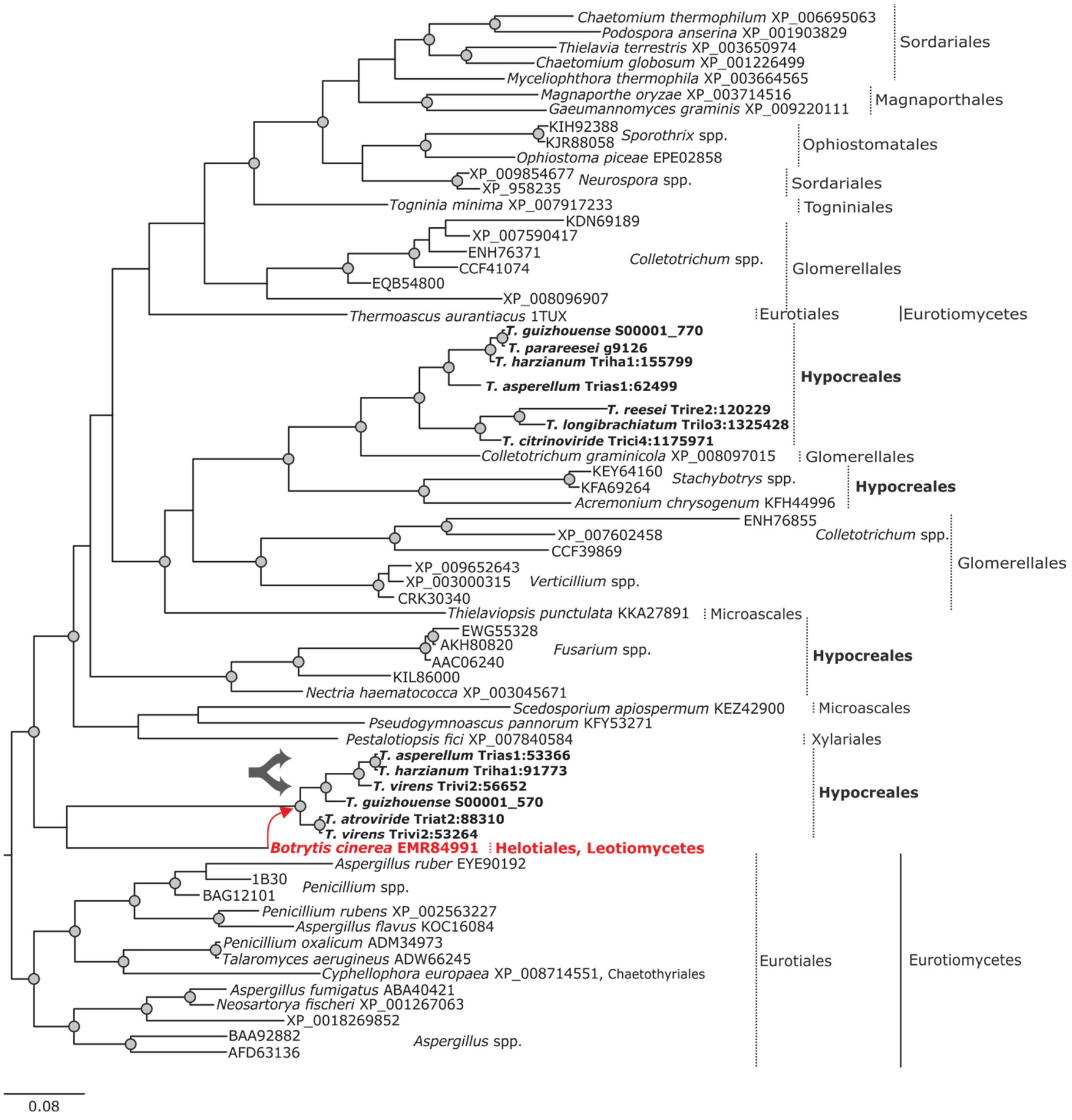

Phylogram based on Dayhoff amino acid substitution model using an alignment containing 21172 characters. Bayesian analysis was run for 1 million mcmc generations and a strict consensus tree was obtained by summarizing 7500 trees, after burning first 25% of obtained 10,000 trees. Mean tree length and variance are 8.903780E+00 and 3.1751560E-02, respectively. Posterior probabilities more than 95% are marked with circular nodes. Dashed vertical bars represents the taxonomic order. All the species belongs to the taxonomic class in the phylum Ascomycota, Sordariomycetes unless marked otherwise. LGT inferred by Phylogeny and NOTUNG is shown with a red arrow to the node and the respective donor is marked in red. The bifurcated arrow indicates duplication as inferred by NOTUNG.

GH11 EC 3.2.1.8 Endo-ß-1,4-xylanase

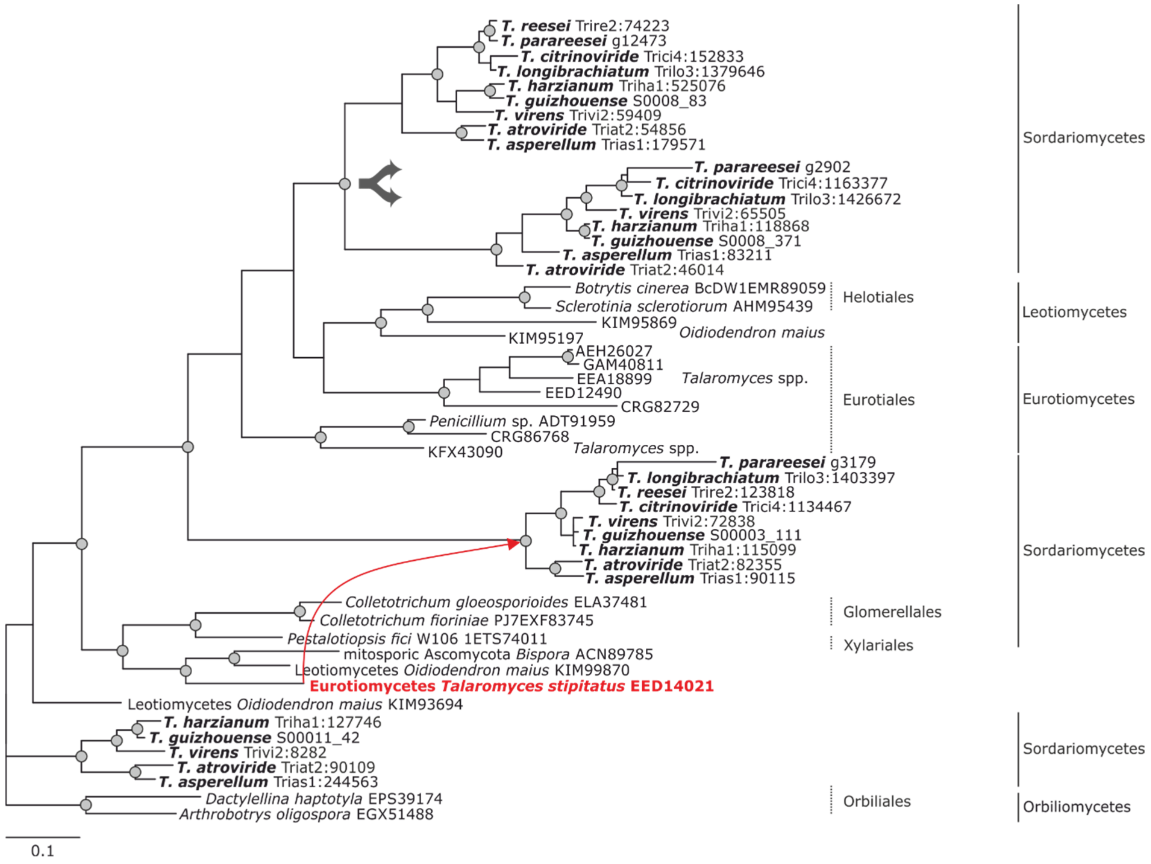

Phylogram based on Dayhoff amino acid substitution model using an alignment containing 18370 characters. Bayesian analysis was run for 0.66 million mcmc generations and a strict consensus tree was obtained by summarizing 4950 trees, after burning first 25% of obtained 6600 trees. Mean tree length and variance are 6.798910E+00 and 3.0536650E-02, respectively. Posterior probabilities more than 95% are marked with circular nodes. Dashed vertical bars and non-dashed vertical bars represents the taxonomic order and taxonomic class in the phylum Ascomycota, respectively. LGT inferred by Phylogeny and NOTUNG is shown with a red arrow to the node and the respective donor is marked in red. The bifurcated arrow indicates duplication as inferred by NOTUNG.

GH30 EC 3.2.1.8 Endo-ß-1,4-xylanase Clade A

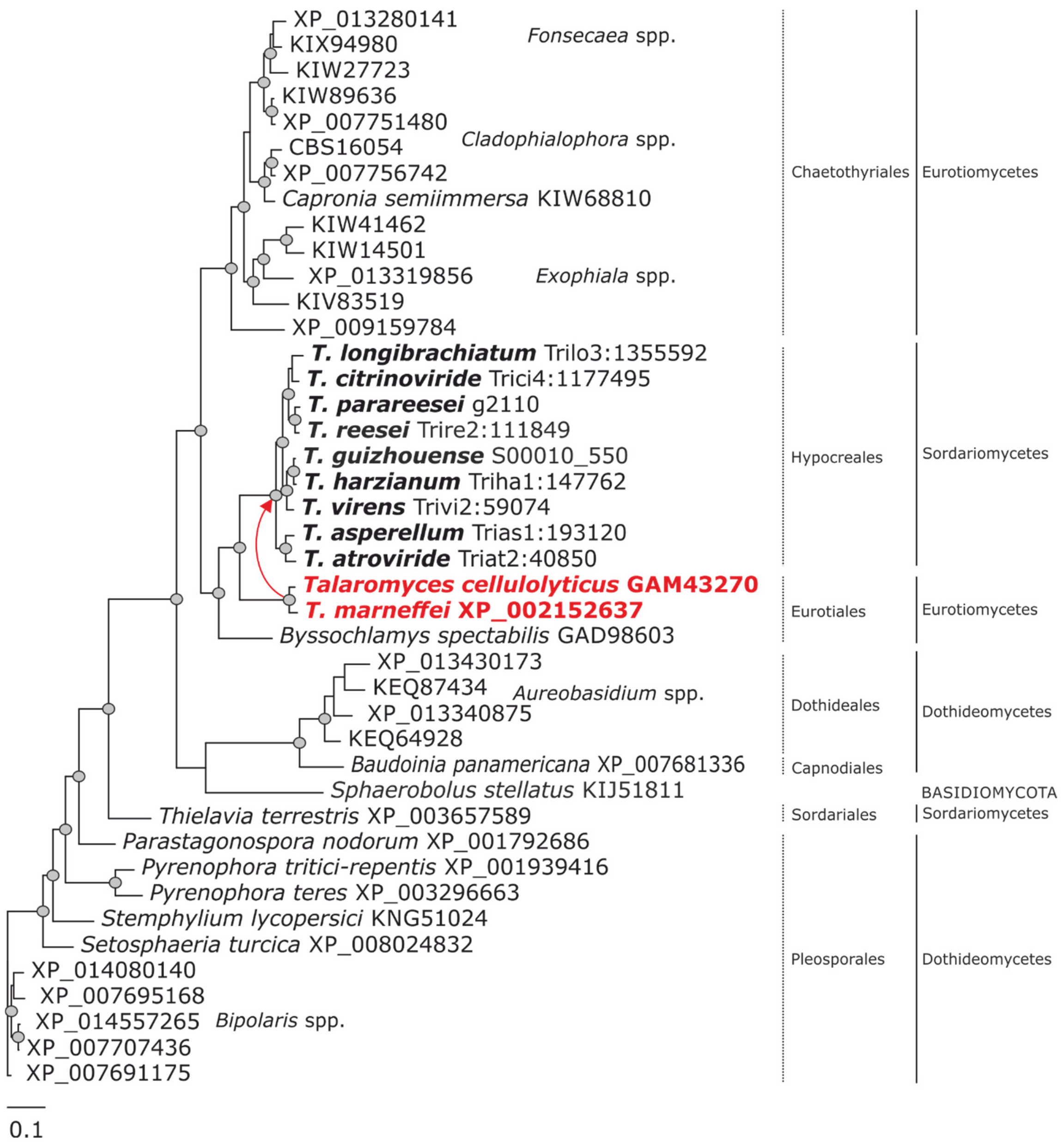

Phylogram based on Dayhoff amino acid substitution model using an alignment containing 44730 characters. Bayesian analysis was run for 1 million mcmc generations and a strict consensus tree was obtained by summarizing 7500 trees, after burning first 25% of obtained 10,000 trees. Mean tree length and variance are 4.169406E+00 and 9.7577200E-03, respectively. Posterior probabilities more than 95% are marked with circular nodes. Dashed vertical bars and non-dashed vertical bars represents the taxonomic order and taxonomic class in the phylum Ascomycota, respectively. LGT inferred by Phylogeny and NOTUNG is shown with a red arrow to the node and the respective donor is marked in red. The bifurcated arrow indicates duplication as inferred by NOTUNG.

GH30 EC 3.2.1.8 Endo-ß-1,4-xylanase Clade B

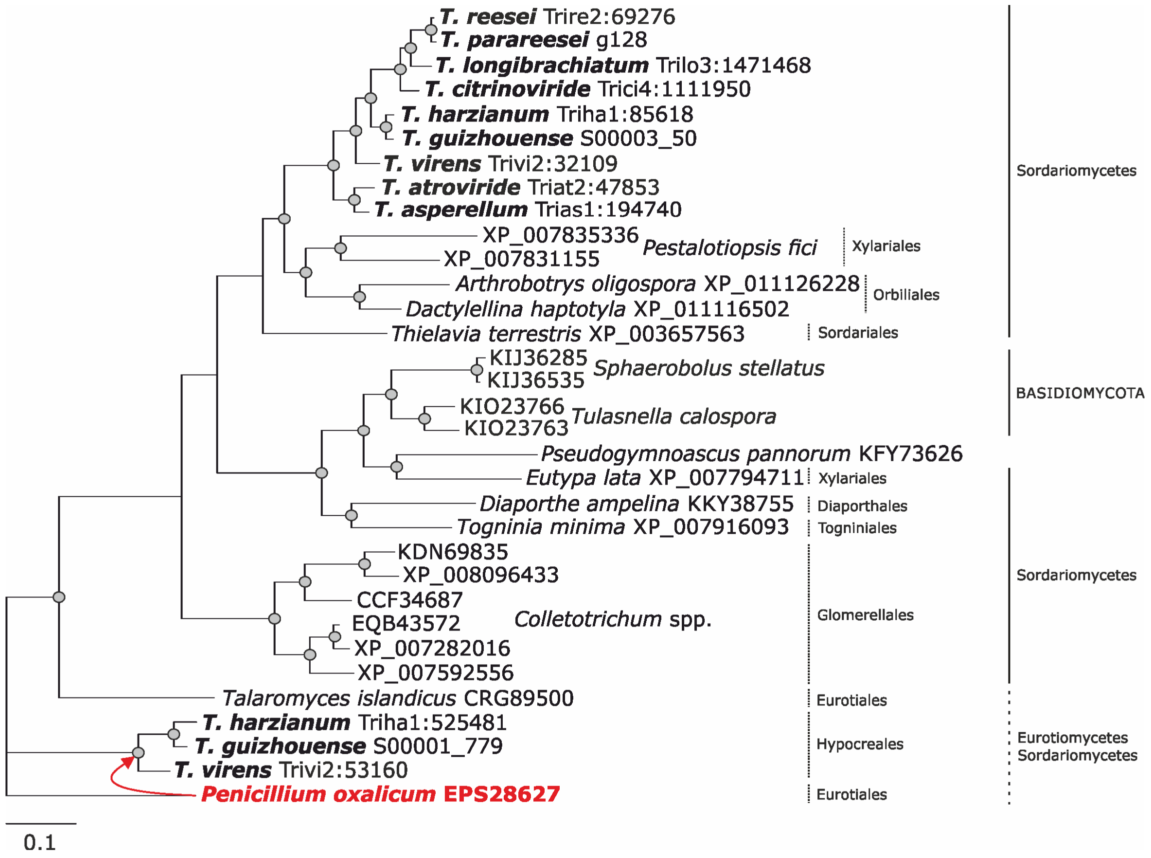

Phylogram based on Dayhoff amino acid substitution model using an alignment containing 15873 characters. Bayesian analysis was run for 1 million mcmc generations and a strict consensus tree was obtained by summarizing 7500 trees, after burning first 25% of obtained 10,000 trees. Mean tree length and variance are 4.491939E+00 and 1.0864500E-02, respectively. Posterior probabilities more than 95% are marked with circular nodes. Dashed vertical bars and non-dashed vertical bars represents the taxonomic order and taxonomic class in the phylum Ascomycota, respectively. LGT inferred by Phylogeny and NOTUNG is shown with a red arrow to the node and the respective donor is marked in red.

GH3 EC 3.2.1.37 Xylan-1,4-ß-xylosidase

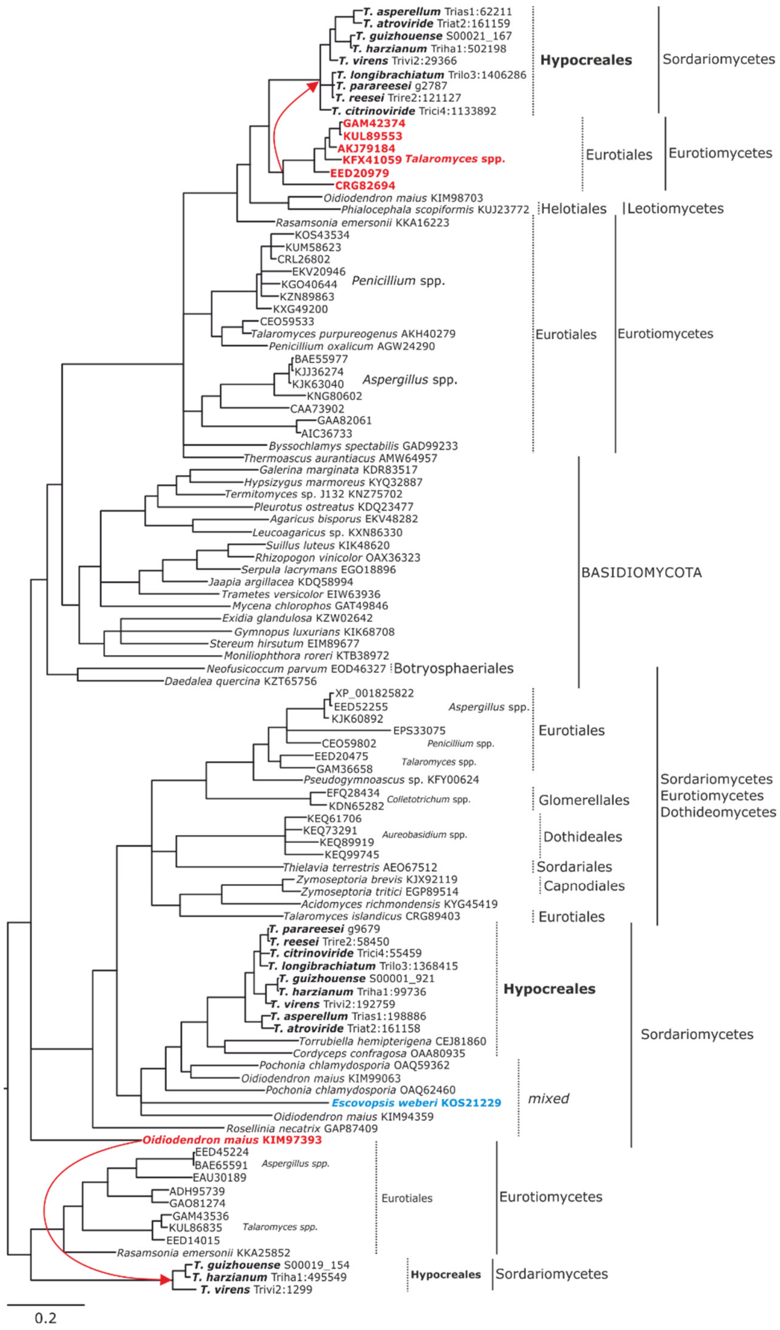

Phylogram based on Dayhoff amino acid substitution model using an alignment containing 82264 characters. Bayesian analysis was run for 2 million mcmc generations and a strict consensus tree was obtained by summarizing 15000 trees, after burning first 25% of obtained 20,000 trees. Mean tree length and variance are 1.676796E+01 and 2.6915590E-02, respectively. Clades with Posterior probabilities less than 95% are collapsed. Dashed vertical bars and non-dashed vertical bars represents the taxonomic order and taxonomic class. LGT inferred by Phylogeny and NOTUNG is shown with a red arrow to the node and the respective donor is marked in red.

GH74 EC3.2.1.151 Xyloglucanase

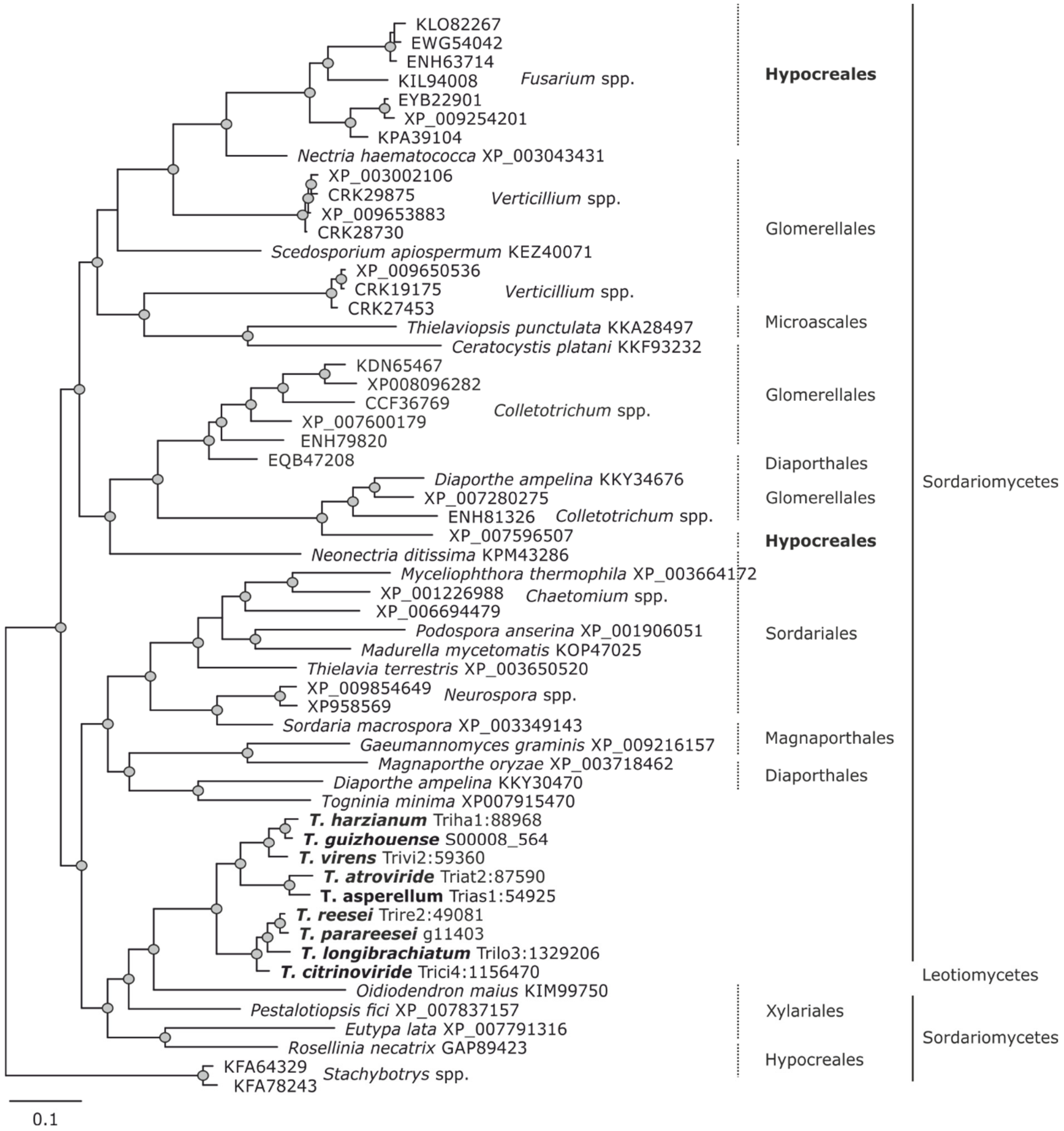

Phylogram based on Dayhoff amino acid substitution model using an alignment containing 80880 characters. Bayesian analysis was run for 1 million mcmc generations and a strict consensus tree was obtained by summarizing 7500 trees, after burning first 25% of obtained 10,000 trees. Mean tree length and variance are 1.334523E+01 and 1.8721190E-02, respectively. Posterior probabilities more than 95% are marked with circular nodes. Dashed vertical bars and non-dashed vertical bars represents the taxonomic order and taxonomic class in the phylum Ascomycota, respectively.

GH5 EC 3.2.1.78 Endo-ß-1,4-mannanase

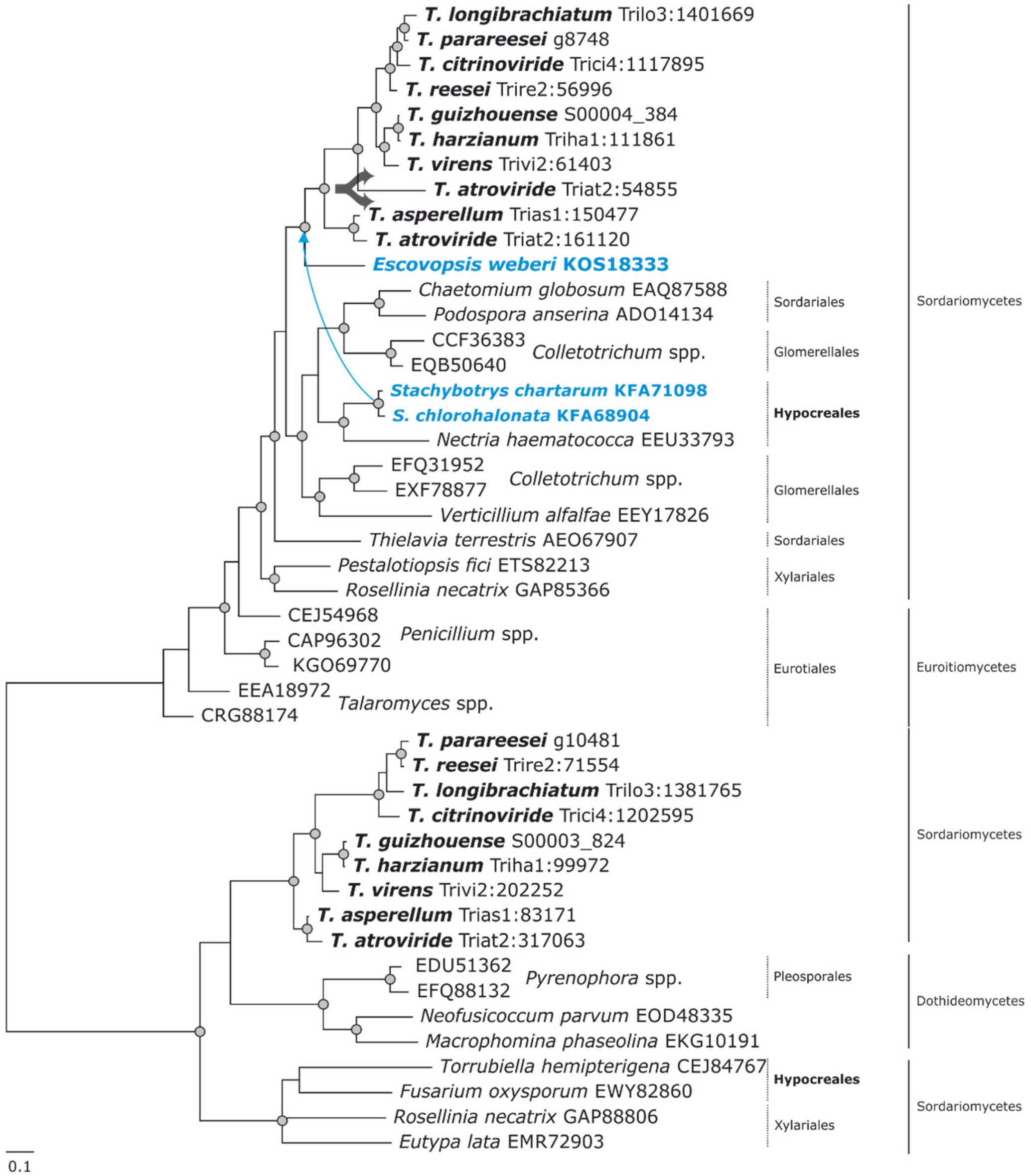

Phylogram based on Dayhoff amino acid substitution model using an alignment containing 30130 characters. Bayesian analysis was run for 1 million mcmc generations and a strict consensus tree was obtained by summarizing 7500 trees, after burning first 25% of obtained 10,000 trees. Mean tree length and variance are 1.147689E+01 and 4.8778060E-02, respectively. Posterior probabilities more than 95% are marked with circular nodes. Dashed vertical bars and non-dashed vertical bars represents the taxonomic order and taxonomic class in the phylum Ascomycota, respectively. LGT inferred by Phylogeny and NOTUNG is shown with a blue arrow to the node and the respective donor is marked in blue. The bifurcated arrow indicates duplication as inferred by NOTUNG.

GH26 EC 3.2.1.78 Endo-ß-1,4-mannanase

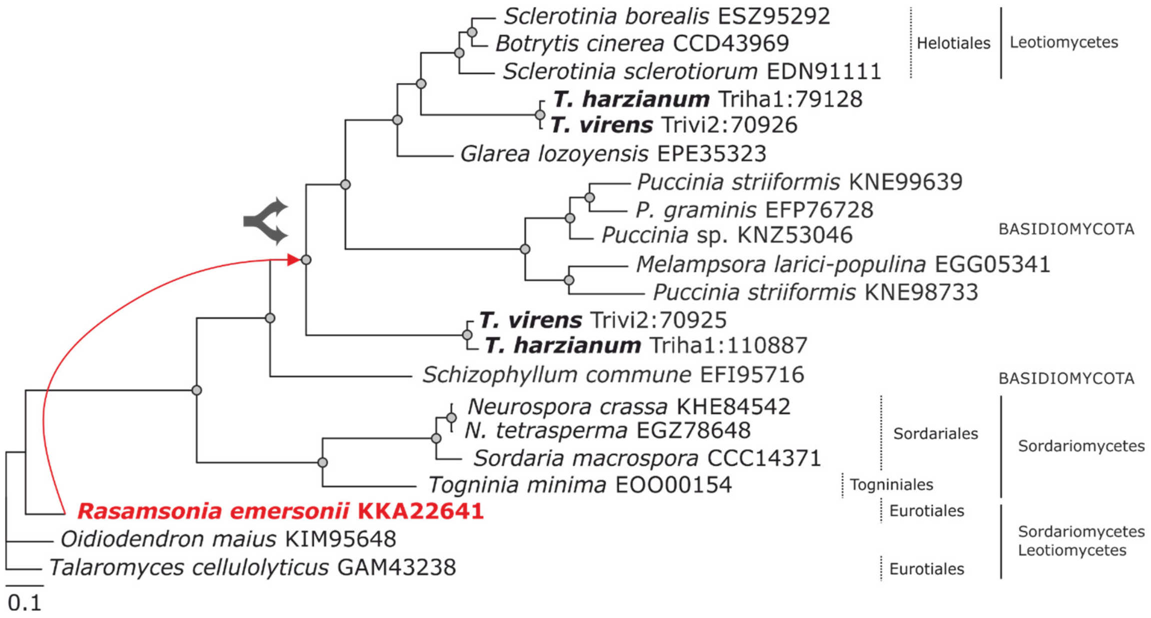

Phylogram based on Dayhoff amino acid substitution model using an alignment containing 6930 characters. Bayesian analysis was run for 1 million mcmc generations and a strict consensus tree was obtained by summarizing 7500 trees, after burning first 25% of obtained 10,000 trees. Mean tree length and variance are 5.565739E+00 and 2.7990880E-02, respectively. Posterior probabilities more than 95% are marked with circular nodes. Dashed vertical bars and non-dashed vertical bars represents the taxonomic order and taxonomic class in the phylum Ascomycota, respectively. LGT inferred by Phylogeny and NOTUNG is shown with a red arrow to the node and the respective donor is marked in red. The bifurcated arrow indicates duplication as inferred by NOTUNG.

GH27 EC 3.2.1.22 α-1,4-galactosidase

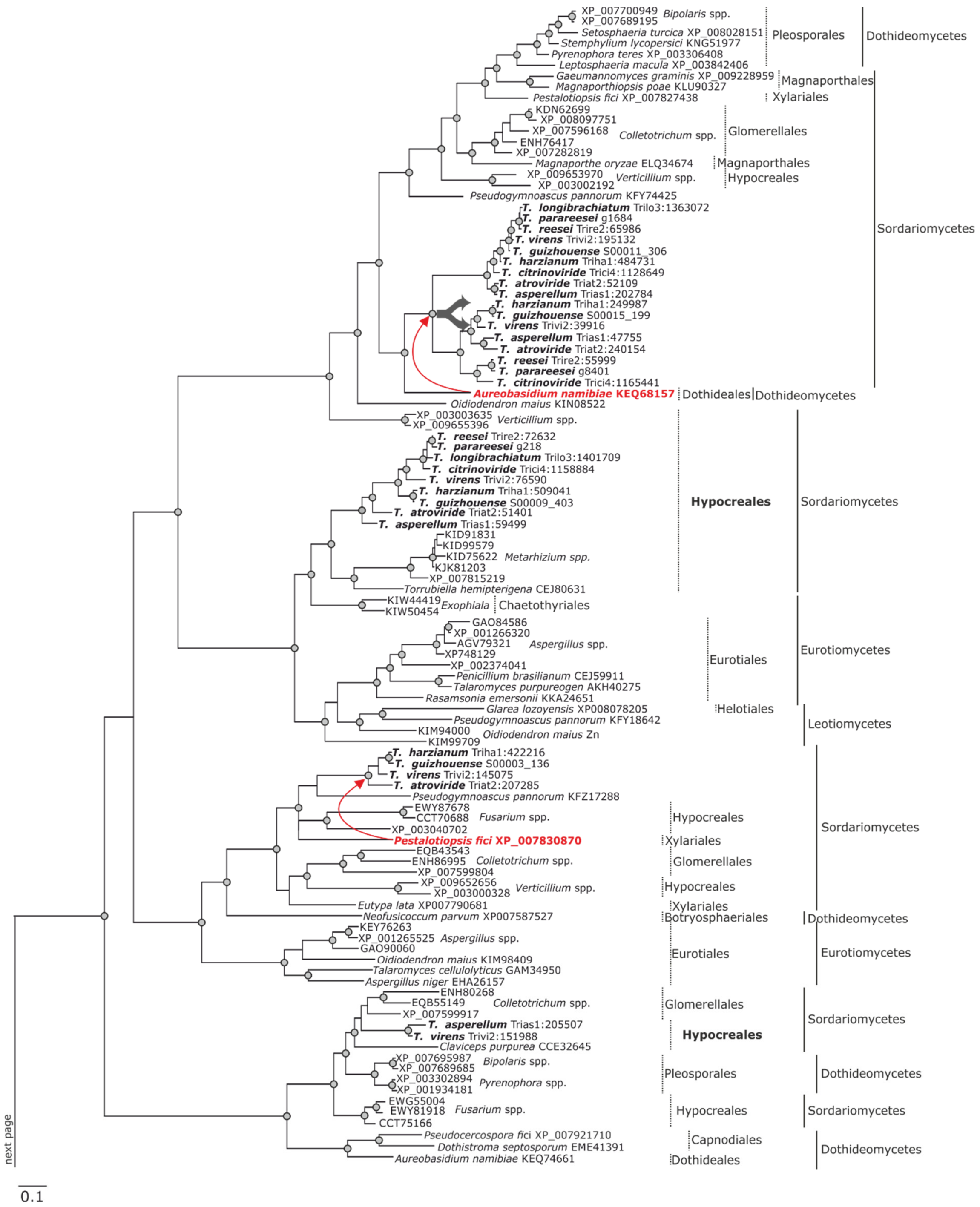

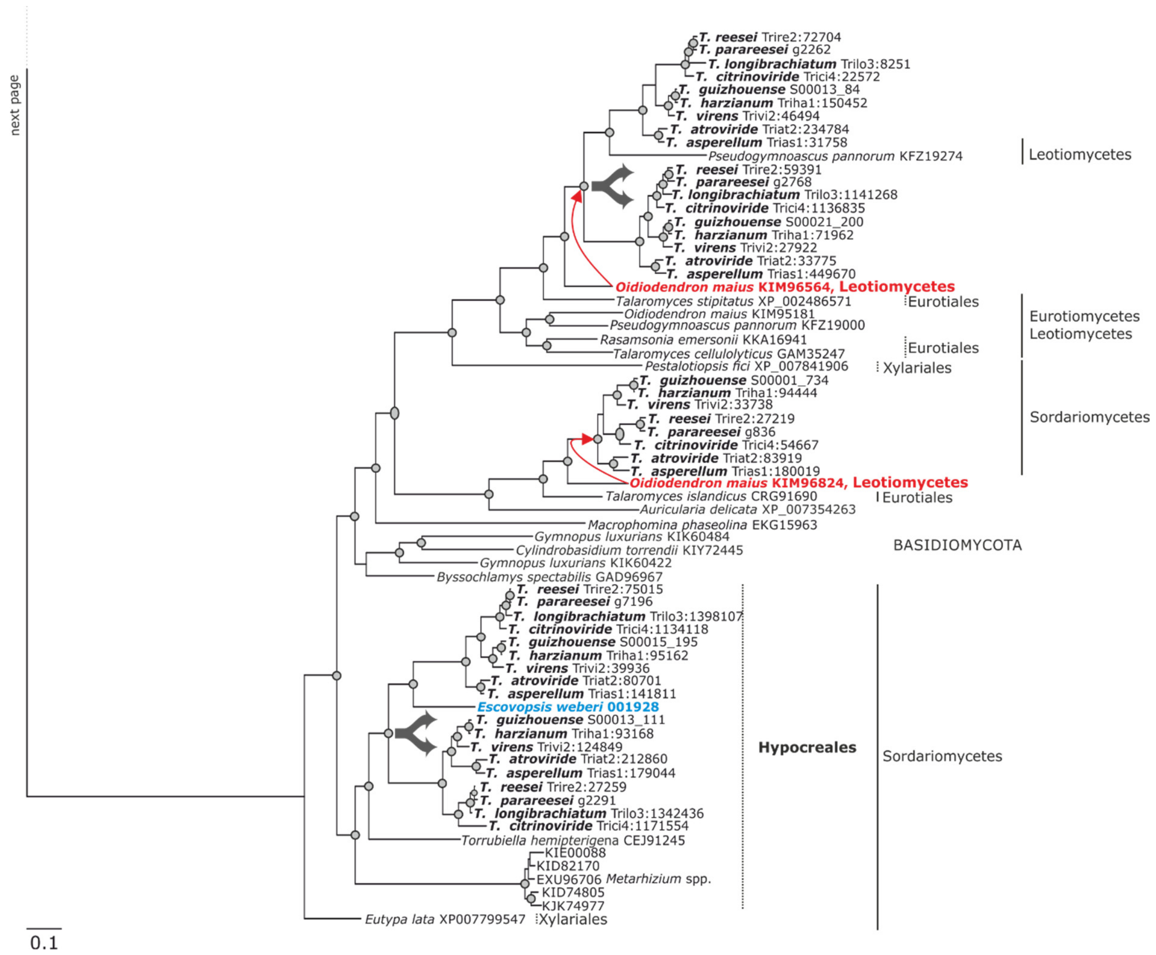

Phylogram based on Dayhoff amino acid substitution model using an alignment containing 148596 characters. Bayesian analysis was run for 5 million mcmc generations and a strict consensus tree was obtained by summarizing 37500 trees, after burning first 25% of obtained 50,000 trees. Mean tree length and variance are 3.133589E+01 and 9.2971280E-02, respectively. Posterior probabilities more than 95% are marked with circular nodes. Dashed vertical bars and non-dashed vertical bars represents the taxonomic order and taxonomic class in the phylum Ascomycota, respectively. LGT inferred by Phylogeny and NOTUNG is shown with a red arrow to the node and the respective donor is marked in red. The bifurcated arrow indicates duplication as inferred by NOTUNG.

GH36 EC 3.2.1.22 α-1,4-galactosidases clade A

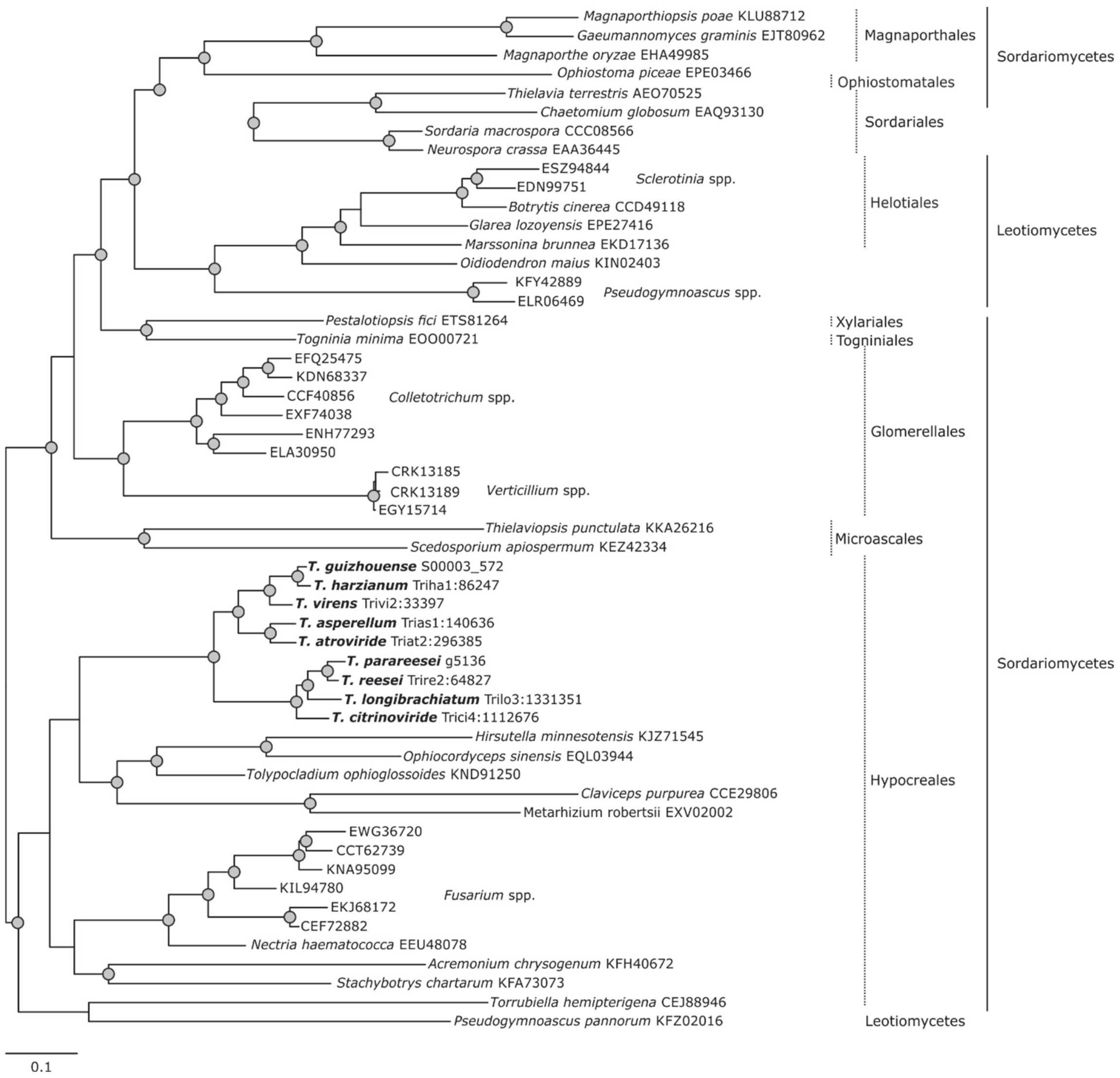

Phylogram based on Dayhoff amino acid substitution model using an alignment containing 33220 characters. Bayesian analysis was run for 1 million mcmc generations and a strict consensus tree was obtained by summarizing 7500 trees, after burning first 25% of obtained 10,000 trees. Mean tree length and variance are 4.453243e+000 and 6.667453e-003, respectively. Posterior probabilities more than 95% are marked with circular nodes. Dashed vertical bars and non-dashed vertical bars represents the taxonomic order and taxonomic class in the phylum Ascomycota, respectively.

GH36 EC 3.2.1.22 α-1,4-galactosidases clade B

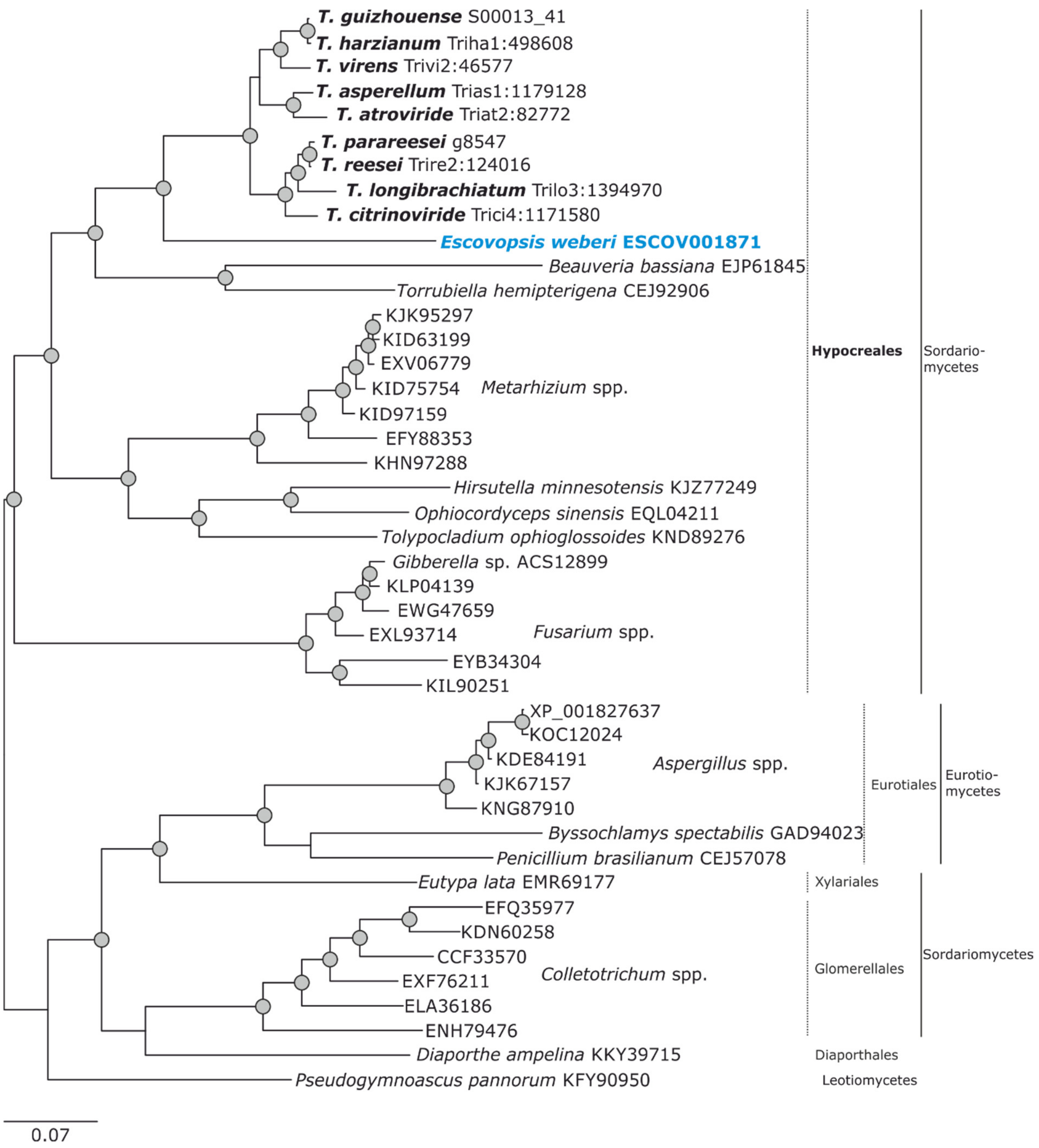

Phylogram based on Dayhoff amino acid substitution model using an alignment containing 51030 characters. Bayesian analysis was run for 1 million mcmc generations and a strict consensus tree was obtained by summarizing 7500 trees, after burning first 25% of obtained 10,000 trees. Mean tree length and variance are 1.242005e+001 and 1.914977e-002, respectively. Posterior probabilities more than 95% are marked with circular nodes. Dashed vertical bars and non-dashed vertical bars represents the taxonomic order and taxonomic class in the phylum Ascomycota, respectively.

GH43 EC 3.2.1.55, EC 3.2.1.37 α-L-arabinofuranosidase and ß-xylosidase

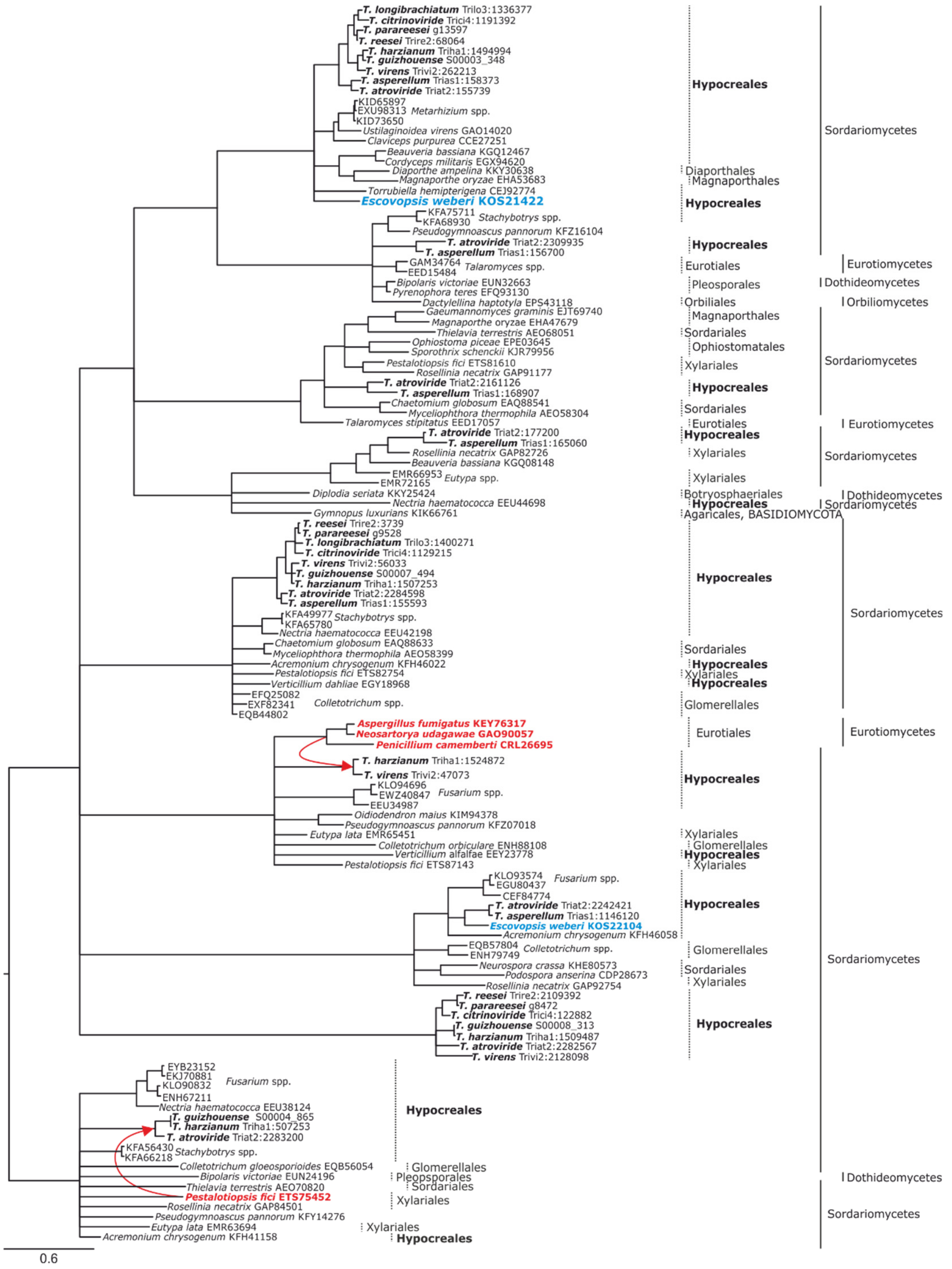

Phylogram based on Dayhoff amino acid substitution model using an alignment containing 99630 characters. Bayesian analysis was run for 3 million mcmc generations and a strict consensus tree was obtained by summarizing 22500 trees, after burning first 25% of obtained 30,000 trees. Mean tree length and variance are 3.687042E+01 and 2.1489000E-01, respectively. Clades with Posterior probabilities less than 95% are collapsed. Dashed vertical bars and non-dashed vertical bars represents the taxonomic order and taxonomic class. LGT inferred by Phylogeny and NOTUNG is shown with a red arrow to the node and the respective donor is marked in red. The bifurcated arrow indicates duplication as inferred by NOTUNG.

GH51 EC 3.2.1.55 α-L-arabinofuranosidase

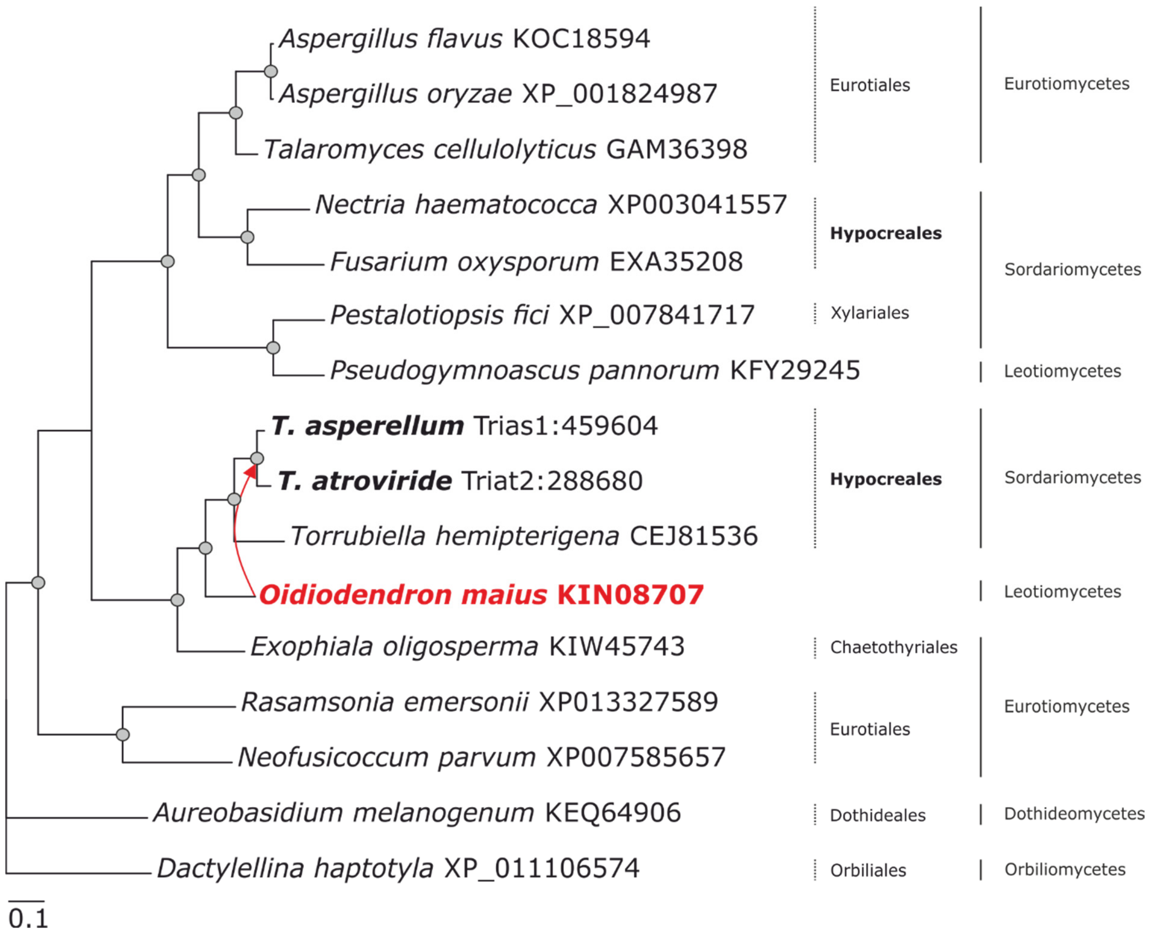

Phylogram based on Dayhoff amino acid substitution model using an alignment containing 10736 characters. Bayesian analysis was run for 1 million mcmc generations and a strict consensus tree was obtained by summarizing 7500 trees, after burning first 25% of obtained 10,000 trees. Mean tree length and variance are 1.940448E+01 and 4.3743370E-02, respectively. Posterior probabilities more than 95% are marked with circular nodes. Dashed vertical bars and non-dashed vertical bars represents the taxonomic order and taxonomic class in the phylum Ascomycota, respectively. LGT inferred by Phylogeny and NOTUNG is shown with a red arrow to the node and the respective donor is marked in red.

GH54 EC 3.2.1.55 α-L-arabinofuranosidase

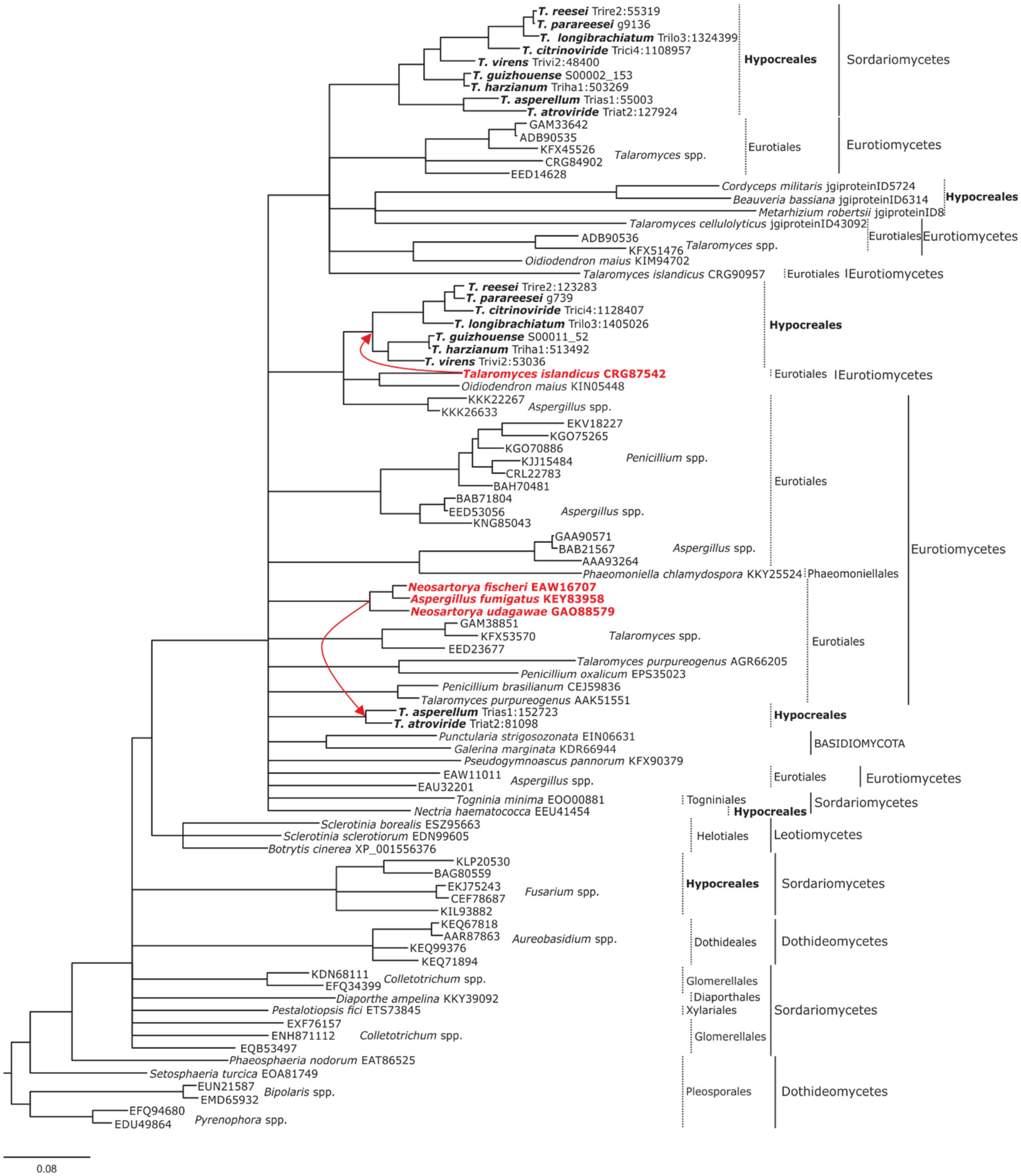

Phylogram based on Dayhoff amino acid substitution model using an alignment containing 47070 characters. Bayesian analysis was run for 3 million mcmc generations and a strict consensus tree was obtained by summarizing 22500 trees, after burning first 25% of obtained 30,000 trees. Mean tree length and variance are 8.226706E+00 and 1.7511440E-02, respectively. Clades with Posterior probabilities less than 95% are collapsed. Dashed vertical bars and non-dashed vertical bars represents the taxonomic order and taxonomic class. LGT inferred by Phylogeny and NOTUNG is shown with a red arrow to the node and the respective donor is marked in red.

GH62 EC 3.2.1.55 α-L-arabinofuranosidase

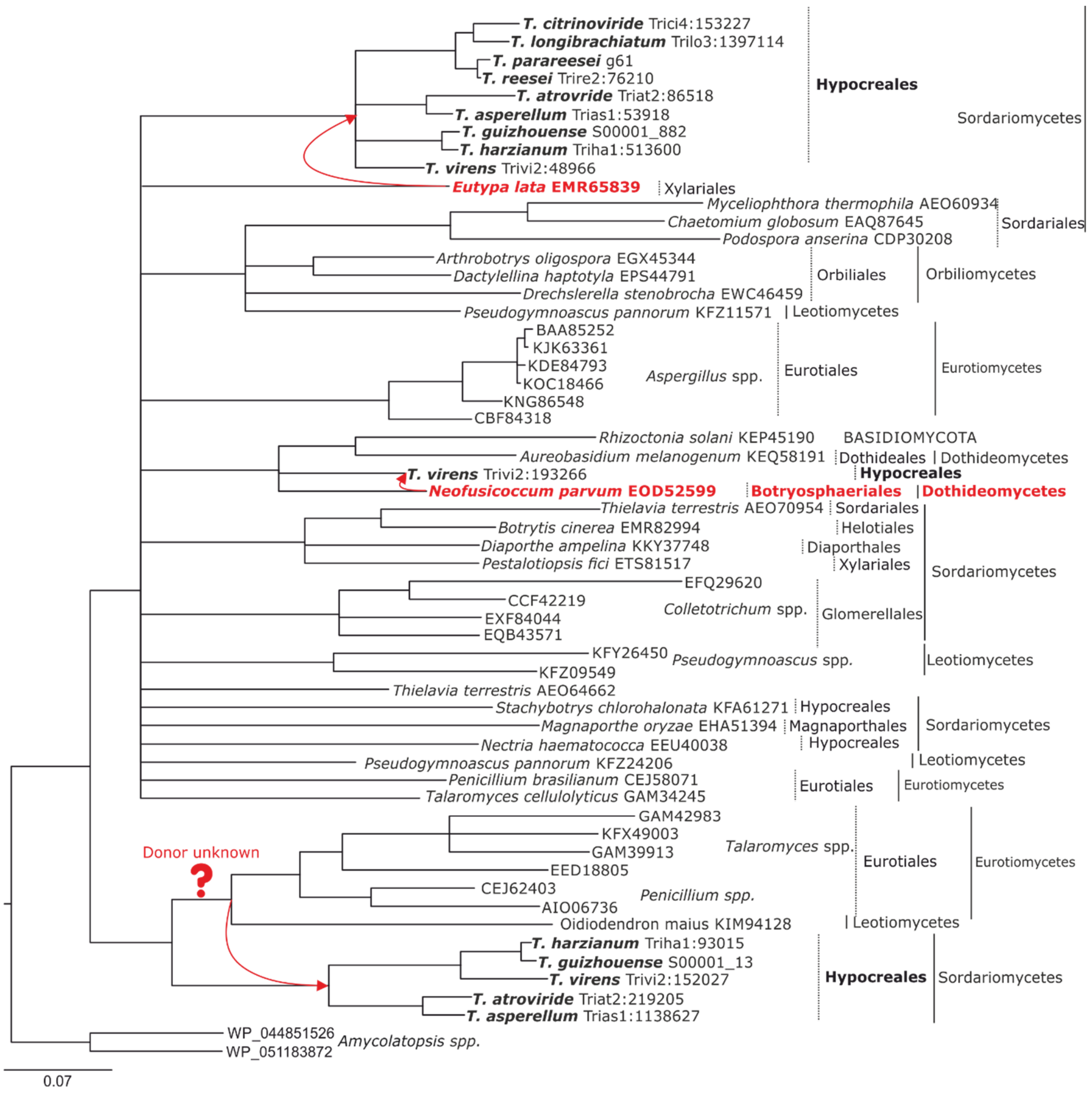

Phylogram based on Dayhoff amino acid substitution model using an alignment containing 18154 characters. Bayesian analysis was run for 1 million mcmc generations and a strict consensus tree was obtained by summarizing 7500 trees, after burning first 25% of obtained 10,000 trees. Mean tree length and variance are 6.723306e+000 and 2.384449e-002, respectively. Clades with Posterior probabilities less than 95% are collapsed. Dashed vertical bars and non-dashed vertical bars represents the taxonomic order and taxonomic class. LGT inferred by Phylogeny and NOTUNG is shown with a red arrow to the node and the respective donor is marked in red.

GH67 EC 3.2.1.131 (xylan) α-1,2-glucuronidase

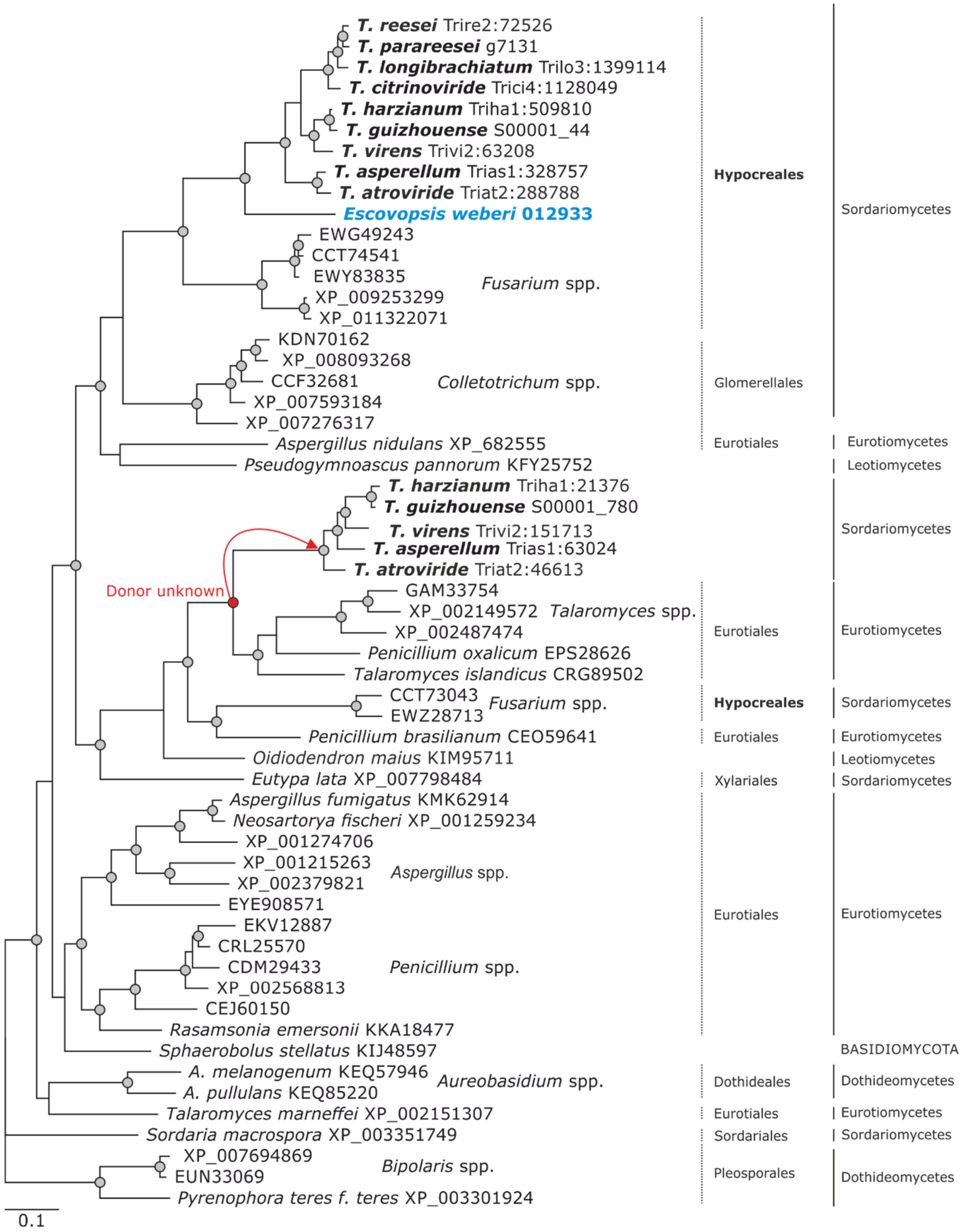

Phylogram based on Dayhoff amino acid substitution model using an alignment containing 50763 characters. Bayesian analysis was run for 1 million mcmc generations and a strict consensus tree was obtained by summarizing 7500 trees, after burning first 25% of obtained 10,000 trees. Mean tree length and variance are 1.058915E+01 and 1.4302340E-02, respectively.Clades with Posterior probabilities less than 95% are marked with circular nodes. Dashed vertical bars and non-dashed vertical bars represents the taxonomic order and taxonomic class. LGT inferred by Phylogeny and NOTUNG is shown with a red arrow to the node and the respective donor is marked in red.

GH115 EC 3.2.1.- α-(4-O-methyl)-glucuronidase

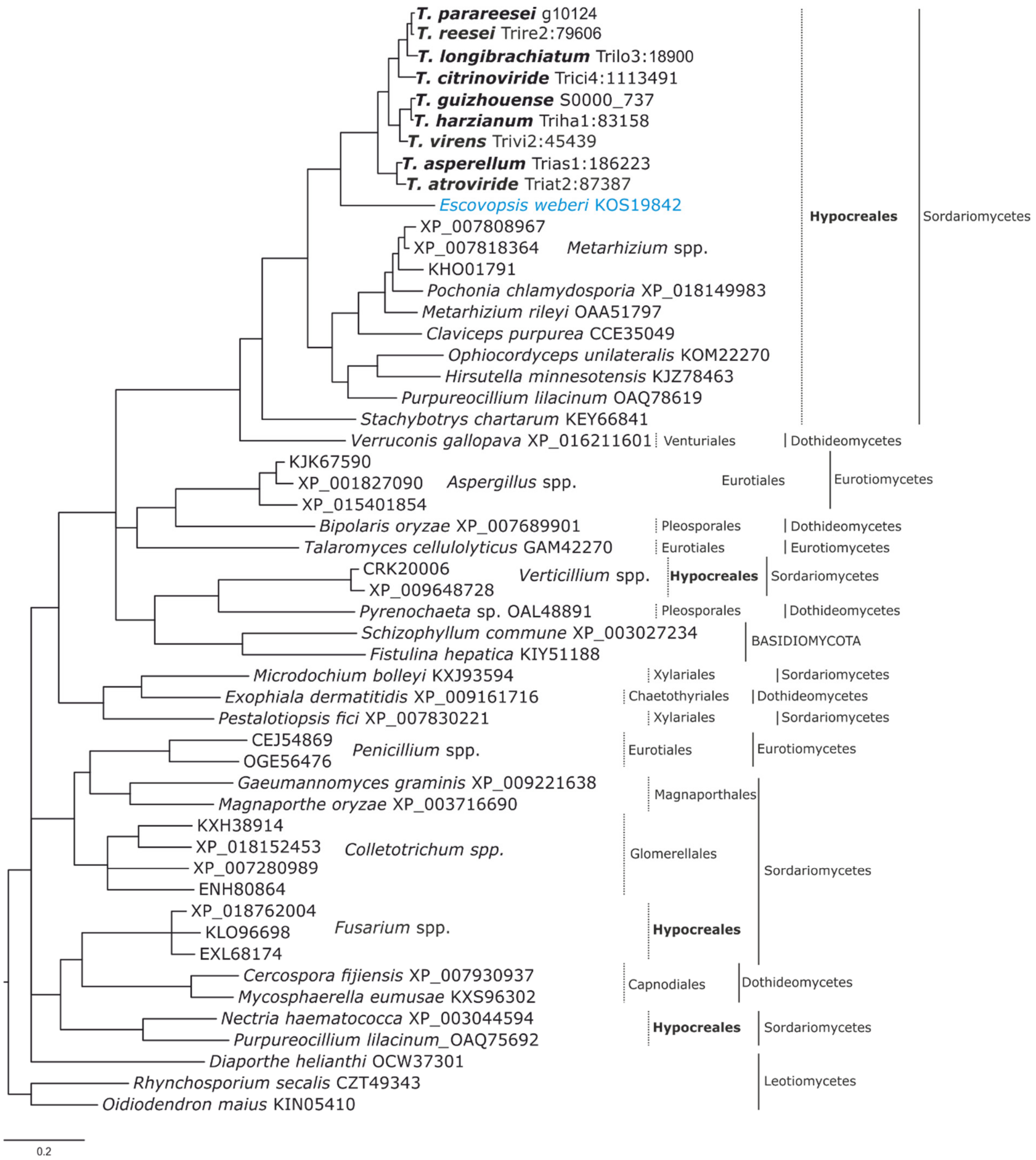

Phylogram based on Dayhoff amino acid substitution model using an alignment containing 57408 characters. Bayesian analysis was run for 1 million mcmc generations and a strict consensus tree was obtained by summarizing 7500 trees, after burning first 25% of obtained 10,000 trees. Mean tree length and variance are 1.70984e+01 and 1.5339040e-02 respectively. Posterior probabilities more than 95% are marked with circular nodes. Dashed vertical bars and non-dashed vertical bars represents the taxonomic order and taxonomic class in the phylum Ascomycota, respectively.

GH95 EC 3.2.1.51 α-L-fucosidase

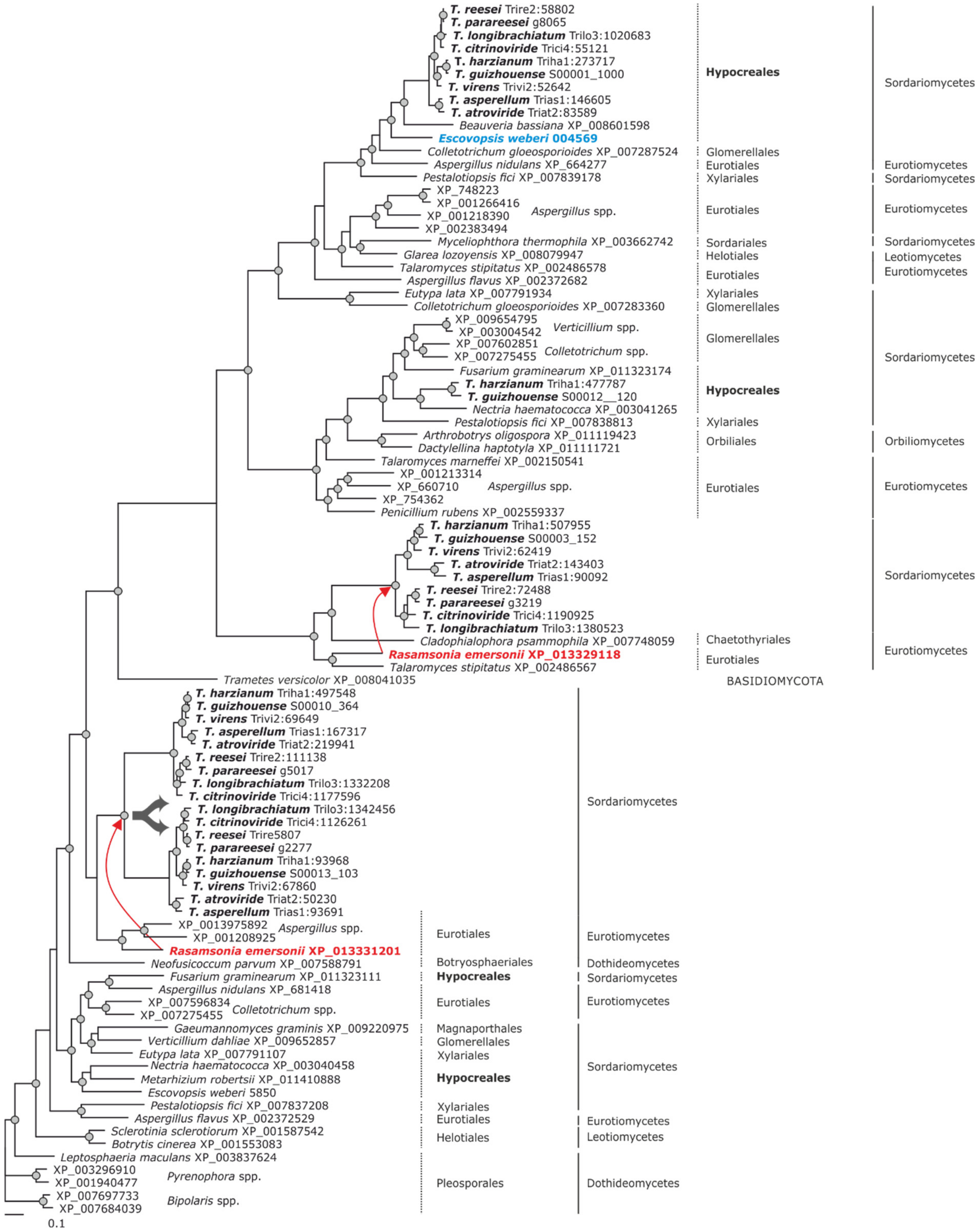

Phylogram based on Dayhoff amino acid substitution model using an alignment containing 86762 characters. Bayesian analysis was run for 1 million mcmc generations and a strict consensus tree was obtained by summarizing 7500 trees, after burning first 25% of obtained 10,000 trees. Mean tree length and variance are 2.349982E+01 and 4.1706740E-02, respectively. Clades with Posterior probabilities less than 95% are marked with circular nodes. LGT inferred by Phylogeny and NOTUNG is shown with a red arrow to the node and the respective donor is marked in red. The bifurcated arrow indicates duplication as inferred by NOTUNG.

GH28 EC 3.2.1.67, EC 3.2.1.171 Exo-polygalacturonase and rhamnogalacturonase

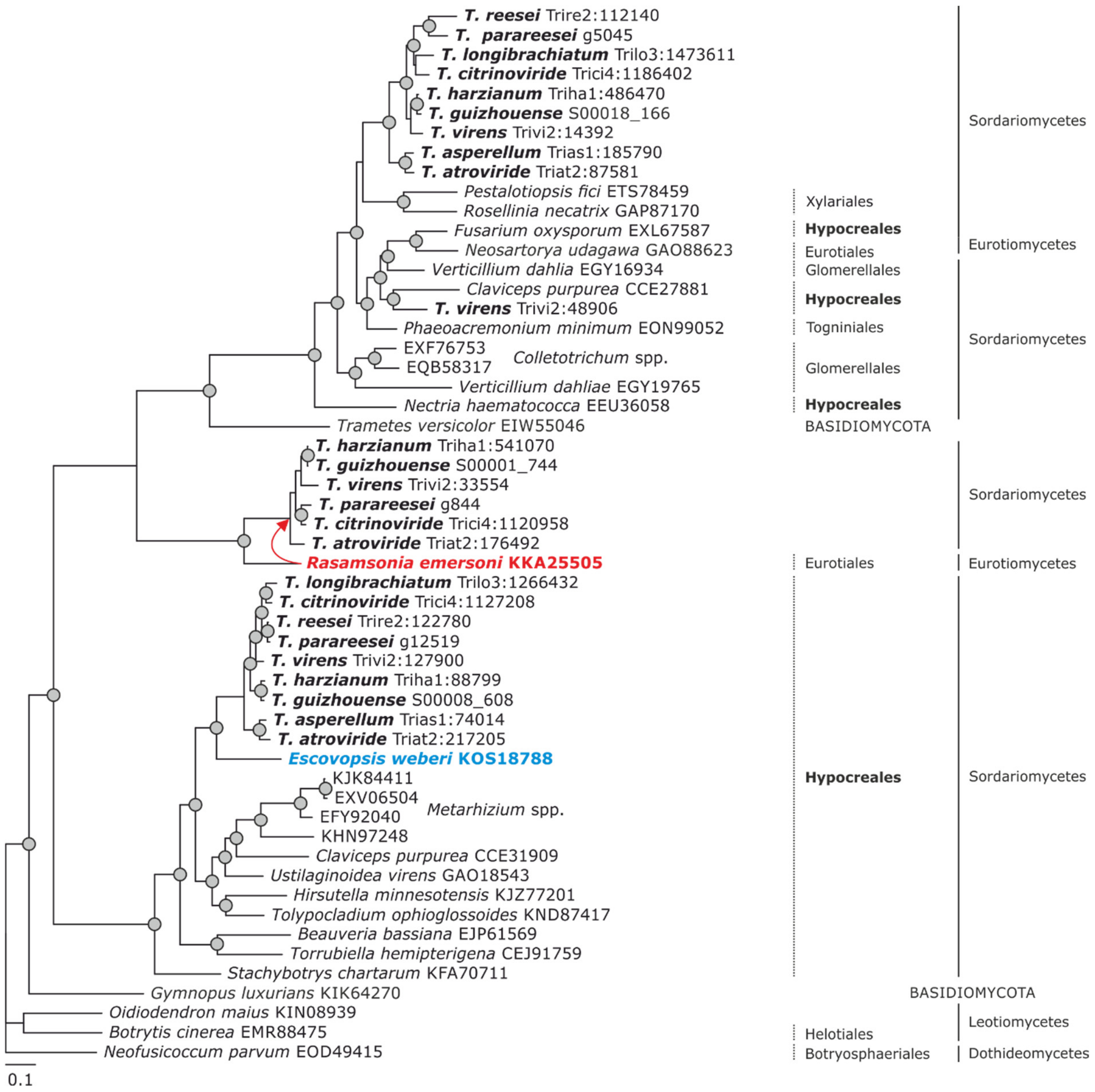

Phylogram based on Dayhoff amino acid substitution model using an alignment containing 22626 characters. Bayesian analysis was run for 1 million mcmc generations and a strict consensus tree was obtained by summarizing 7500 trees, after burning first 25% of obtained 10,000 trees. Mean tree length and variance are 1.046359E+01 and 3.3770210E-02, respectively. Clades with Posterior probabilities less than 95% are marked with circular nodes. Dashed vertical bars and non-dashed vertical bars represents the taxonomic order and taxonomic class. LGT inferred by Phylogeny and NOTUNG is shown with a red arrow to the node and the respective donor is marked in red.

GH28 EC 3.2.1.15 Polygalacturonase

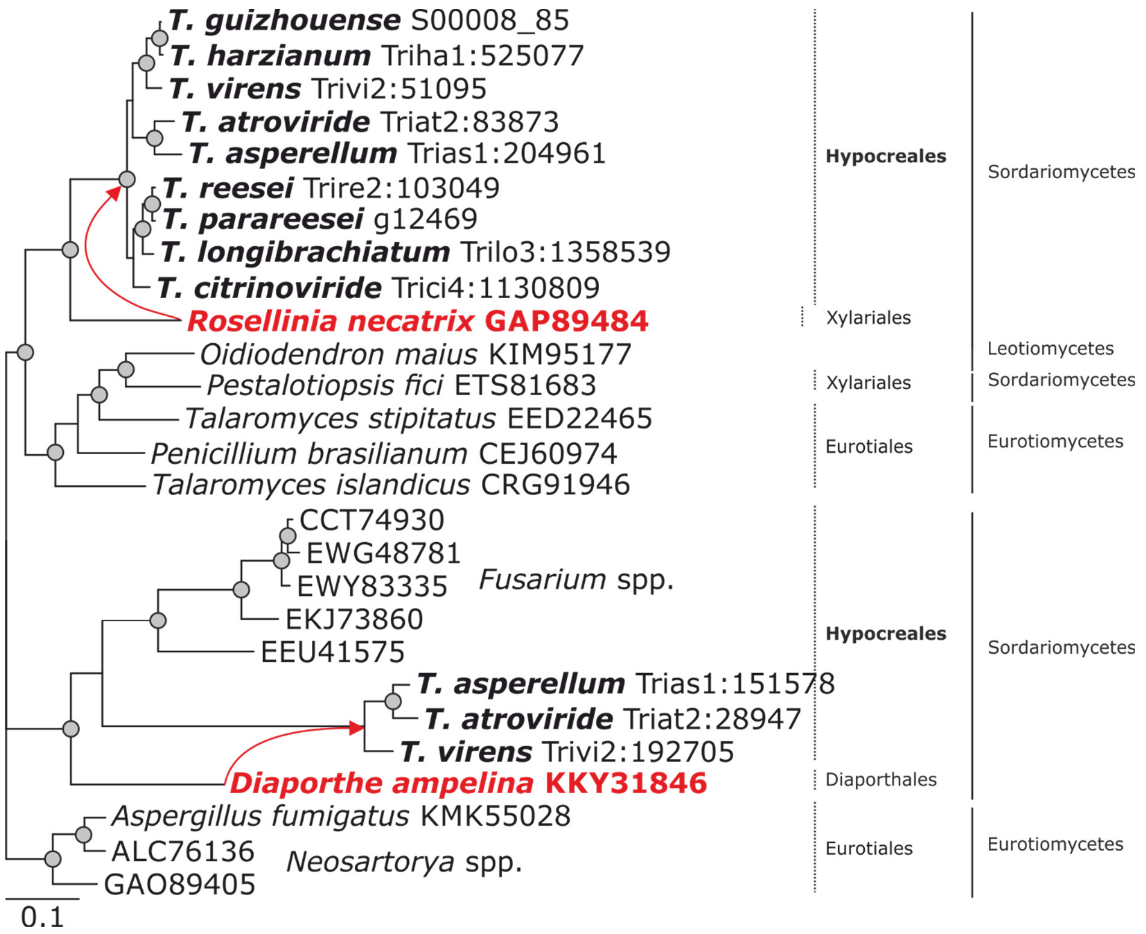

Phylogram based on Dayhoff amino acid substitution model using an alignment containing 10611 characters. Bayesian analysis was run for 1 million mcmc generations and a strict consensus tree was obtained by summarizing 7500 trees, after burning first 25% of obtained 10,000 trees. Mean tree length and variance are 2.800150E+00 and 8.0533830E-03, respectively. Clades with Posterior probabilities less than 95% are marked with circular nodes. Dashed vertical bars and non-dashed vertical bars represents the taxonomic order and taxonomic class. LGT inferred by Phylogeny and NOTUNG is shown with a red arrow to the node and the respective donor is marked in red.

GH28 EC 3.2.1.- Exo-xylogalacturan hydrolase

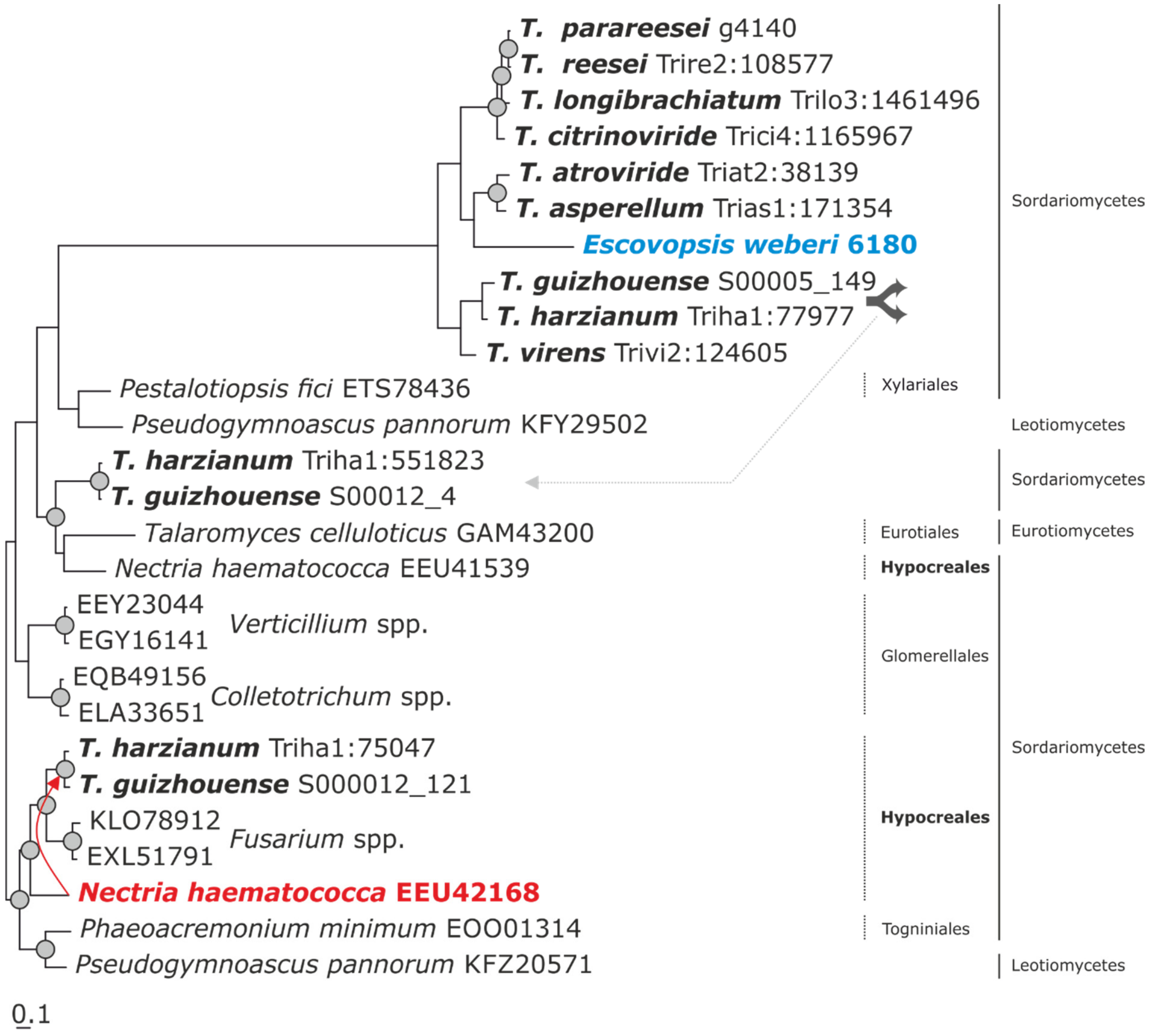

Phylogram based on Dayhoff amino acid substitution model using an alignment containing 21349 characters. Bayesian analysis was run for 1 million mcmc generations and a strict consensus tree was obtained by summarizing 7500 trees, after burning first 25% of obtained 10,000 trees. Mean tree length and variance are 1.138844E+01 and 8.8116490E-02, respectively. Clades with Posterior probabilities less than 95% are marked with circular nodes. Dashed vertical bars and non-dashed vertical bars represents the taxonomic order and taxonomic class. LGT inferred by Phylogeny and NOTUNG is shown with a red arrow to the node and the respective donor is marked in red. The bifurcated arrow indicates duplication as inferred by NOTUNG.

GH78 EC 3.2.1.40 α-L-rhamnosidase

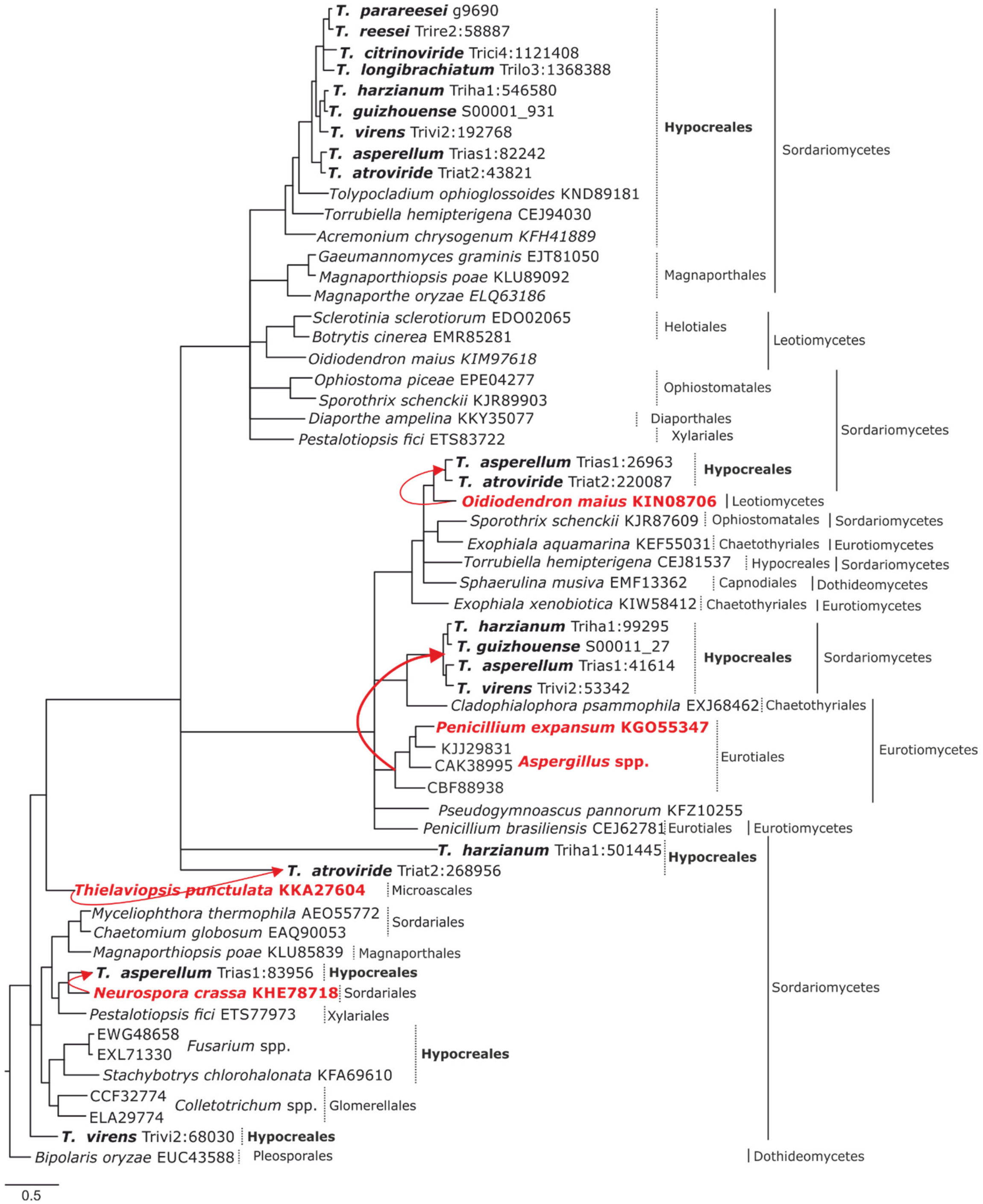

Phylogram based on Dayhoff amino acid substitution model using an alignment containing 62700 characters. Bayesian analysis was run for 1 million mcmc generations and a strict consensus tree was obtained by summarizing 7500 trees, after burning first 25% of obtained 10,000 trees. Mean tree length and variance are 1.058915E+01 and 1.4302340E-02, respectively. Clades with Posterior probabilities less than 95% are collapsed. Dashed vertical bars and non-dashed vertical bars represents the taxonomic order and taxonomic class. LGT inferred by Phylogeny and NOTUNG is shown with a red arrow to the node and the respective donor is marked in red. The bifurcated arrow indicates duplication as inferred by NOTUNG.

PL1 EC 4.2.2.2 Pectate lyase

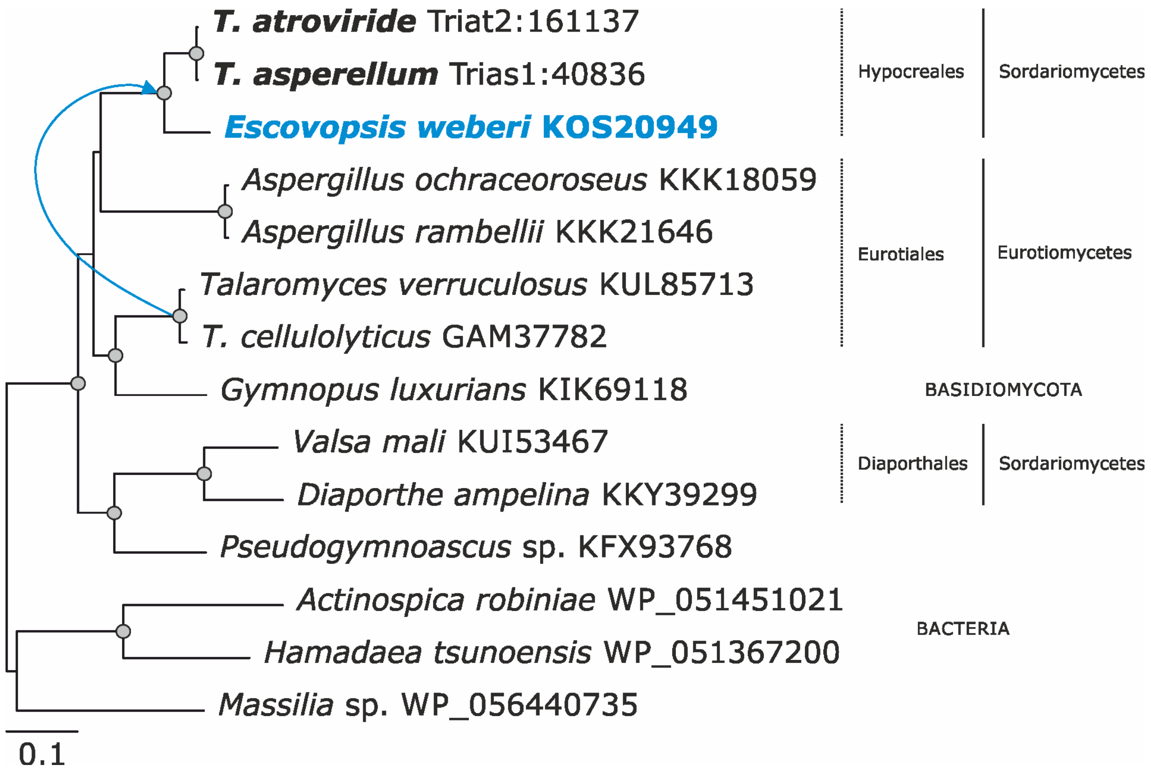

Phylogram based on Dayhoff amino acid substitution model using an alignment containing 4858 characters. Bayesian analysis was run for 1 million mcmc generations and a strict consensus tree was obtained by summarizing 7500 trees, after burning first 25% of obtained 10,000 trees. Mean tree length and variance are 2.165486E+00 and 7.1493260E-03, respectively. Clades with Posterior probabilities less than 95% are marked with circular nodes. Dashed vertical bars and non-dashed vertical bars represents the taxonomic order and taxonomic class.

GH35 EC 3.2.1.23 ß-galactosidase

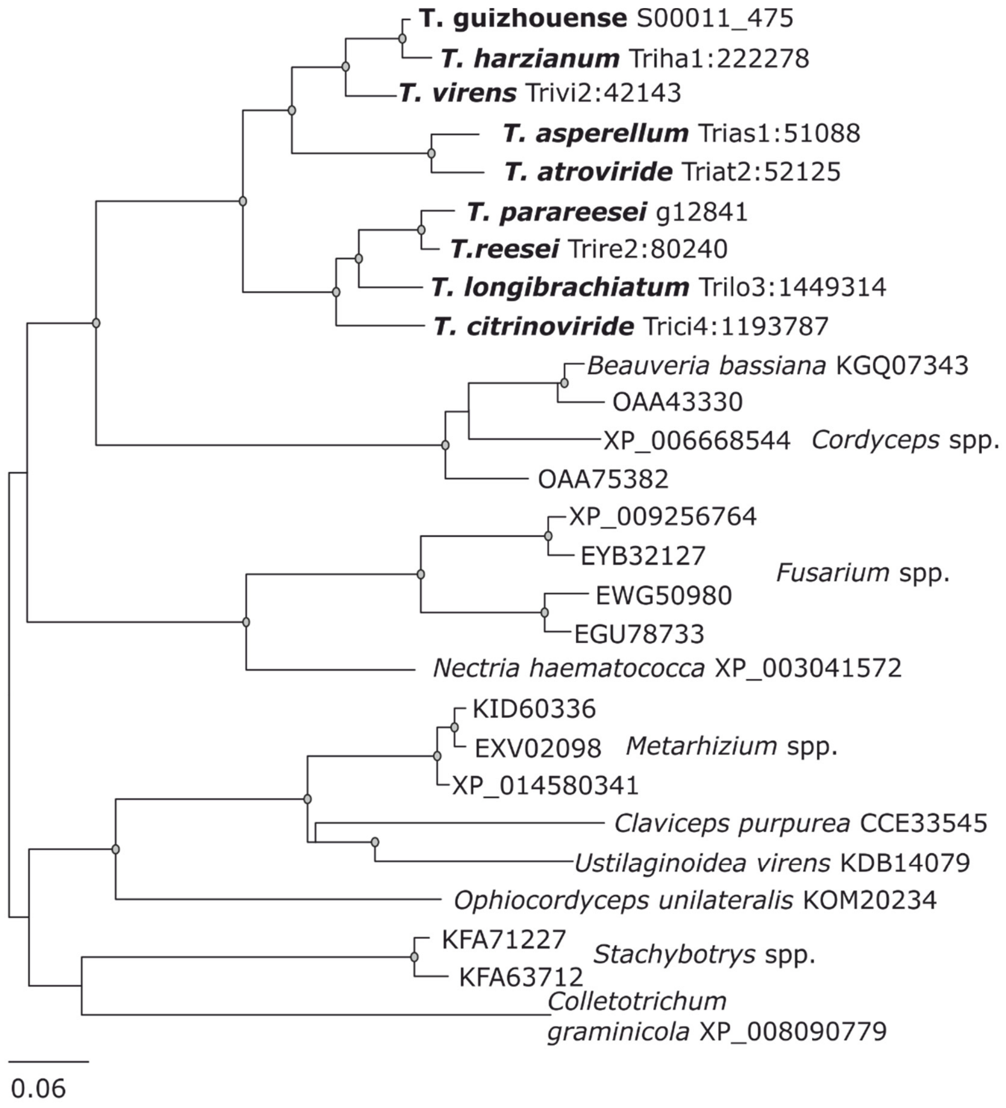

***All species fall under taxonomic class Sordariomycetes and taxonomic order Hypocreales, except *Colletotrichum graminicola* which is from order Glomerellales**

Phylogram based on Dayhoff amino acid substitution model using an alignment containing 24794 characters. Bayesian analysis was run for 1 million mcmc generations and a strict consensus tree was obtained by summarizing 7500 trees, after burning first 25% of obtained 10,000 trees. Mean tree length and variance are 3.587438E+00 and 4.1878620E-03, respectively. Clades with Posterior probabilities less than 95% are marked with circular nodes. Dashed vertical bars and non-dashed vertical bars represents the taxonomic order and taxonomic class.

GH79 EC 3.2.1.31 β-(4-O-methyl)-glucuronidase

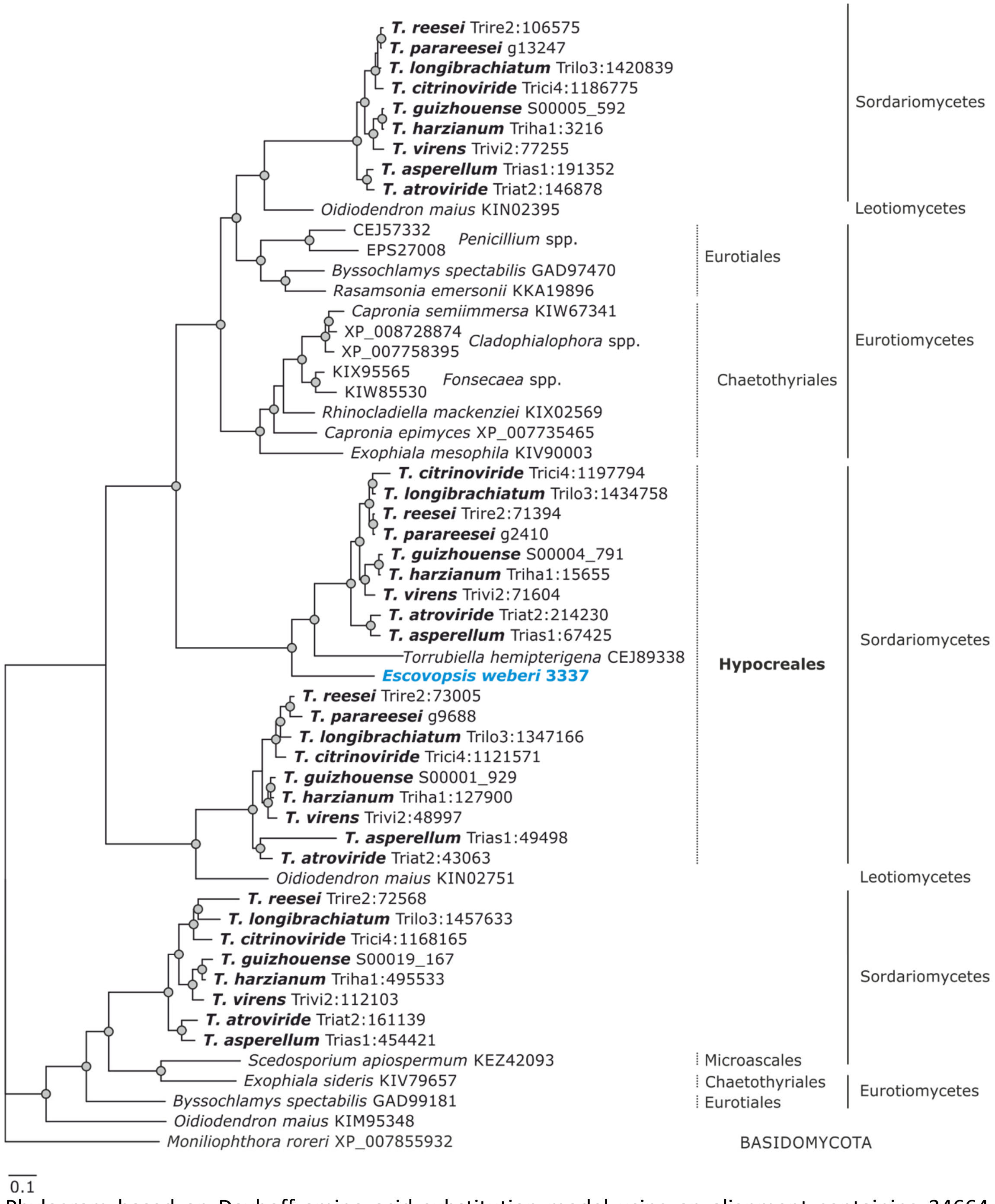

Phylogram based on Dayhoff amino acid substitution model using an alignment containing 34664 characters. Bayesian analysis was run for 1 million mcmc generations and a strict consensus tree was obtained by summarizing 7500 trees, after burning first 25% of obtained 10,000 trees. Mean tree length and variance are 1.069127E+01 and 2.7815940E-02, respectively. Clades with Posterior probabilities less than 95% are marked with circular nodes. Dashed vertical bars and non-dashed vertical bars represents the taxonomic order and taxonomic class.

GH88 EC 3.2.1 Δ-4,5-unsaturated β-glucuronyl hydrolase

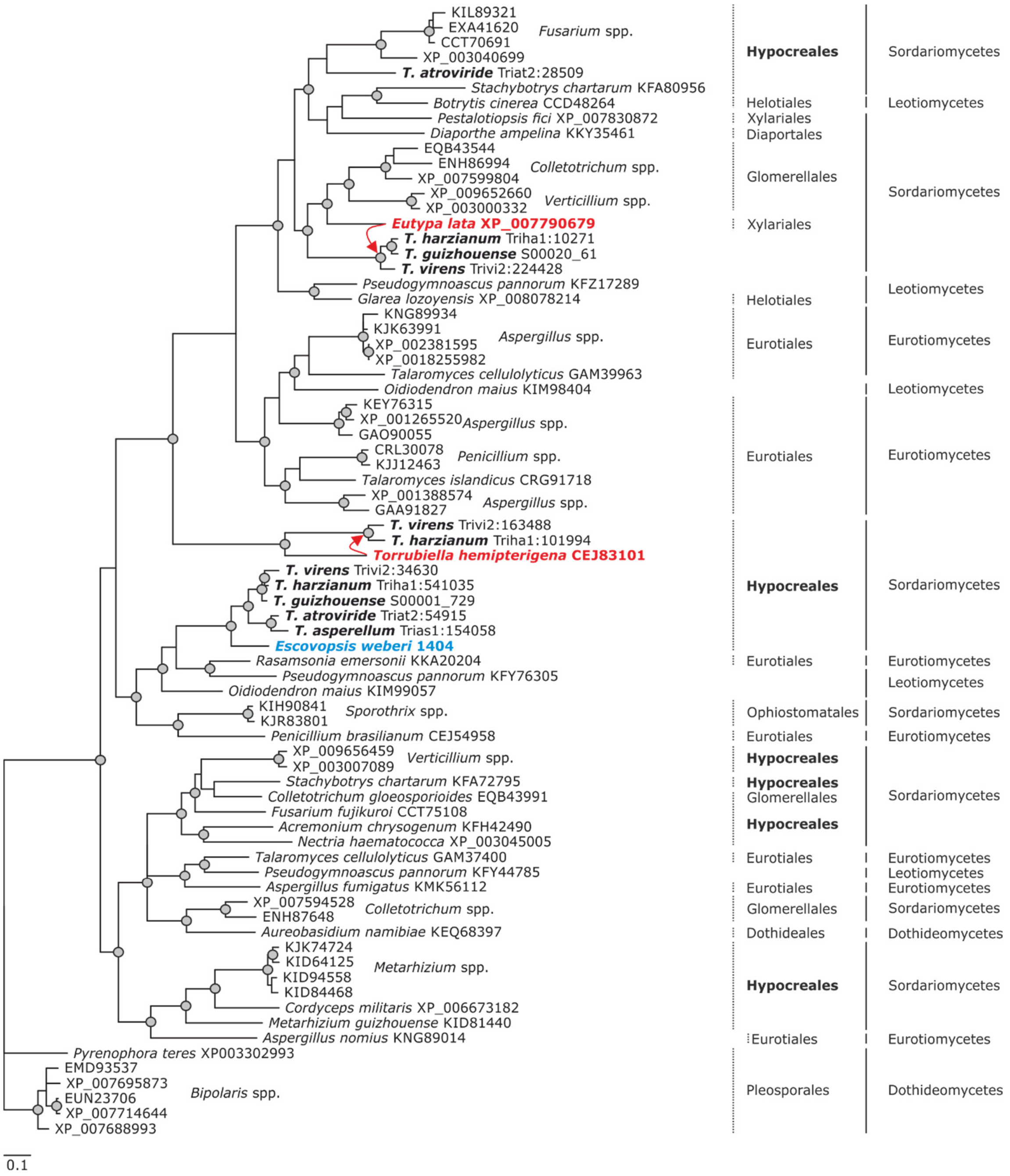

Phylogram based on Dayhoff amino acid substitution model using an alignment containing 9984 characters. Bayesian analysis was run for 1 million mcmc generations and a strict consensus tree was obtained by summarizing 7500 trees, after burning first 25% of obtained 10,000 trees. Mean tree length and variance are 2.334795E+00 and 6.8740380E-03, respectively. Clades with Posterior probabilities less than 95% are marked with circular nodes. Dashed vertical bars and non-dashed vertical bars represents the taxonomic order and taxonomic class. LGT inferred by Phylogeny and NOTUNG is shown with a red arrow to the node and the respective donor is marked in red.

GH105 EC 3.2.1.172 Unsaturated rhamnogalacturonyl hydrolase

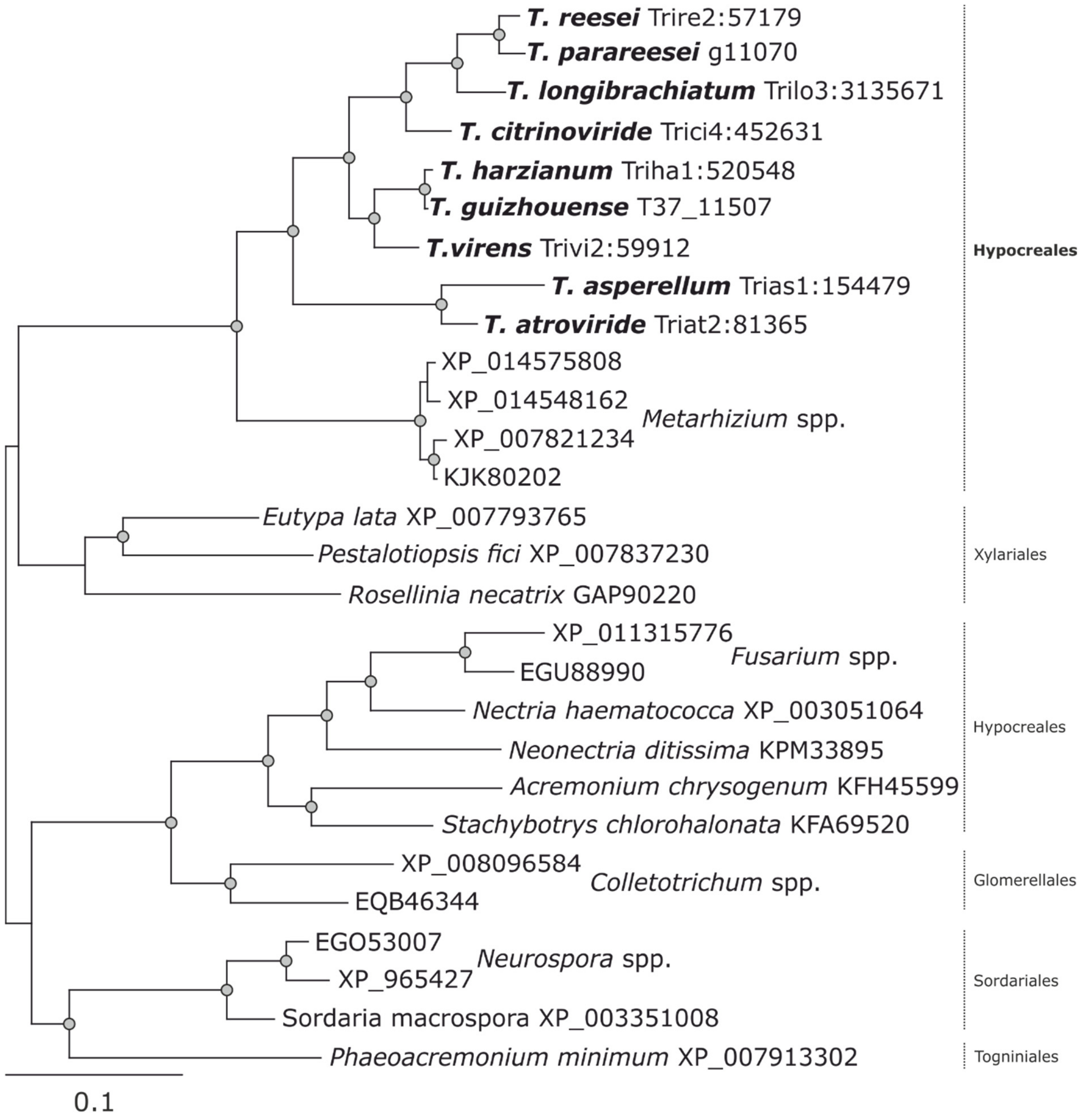

***All species fall under taxonomic class Sordariomycetes**

Phylogram based on Dayhoff amino acid substitution model using an alignment containing 10752 characters. Bayesian analysis was run for 1 million mcmc generations and a strict consensus tree was obtained by summarizing 7500 trees, after burning first 25% of obtained 10,000 trees. Mean tree length and variance are 2.390426E+00 and 7.1870230E-03, respectively. Clades with Posterior probabilities less than 95% are marked with circular nodes. Dashed vertical bars and non-dashed vertical bars represents the taxonomic order and taxonomic class.

GH121 EC 3.2.1.- ß-L-arabinobiosidase

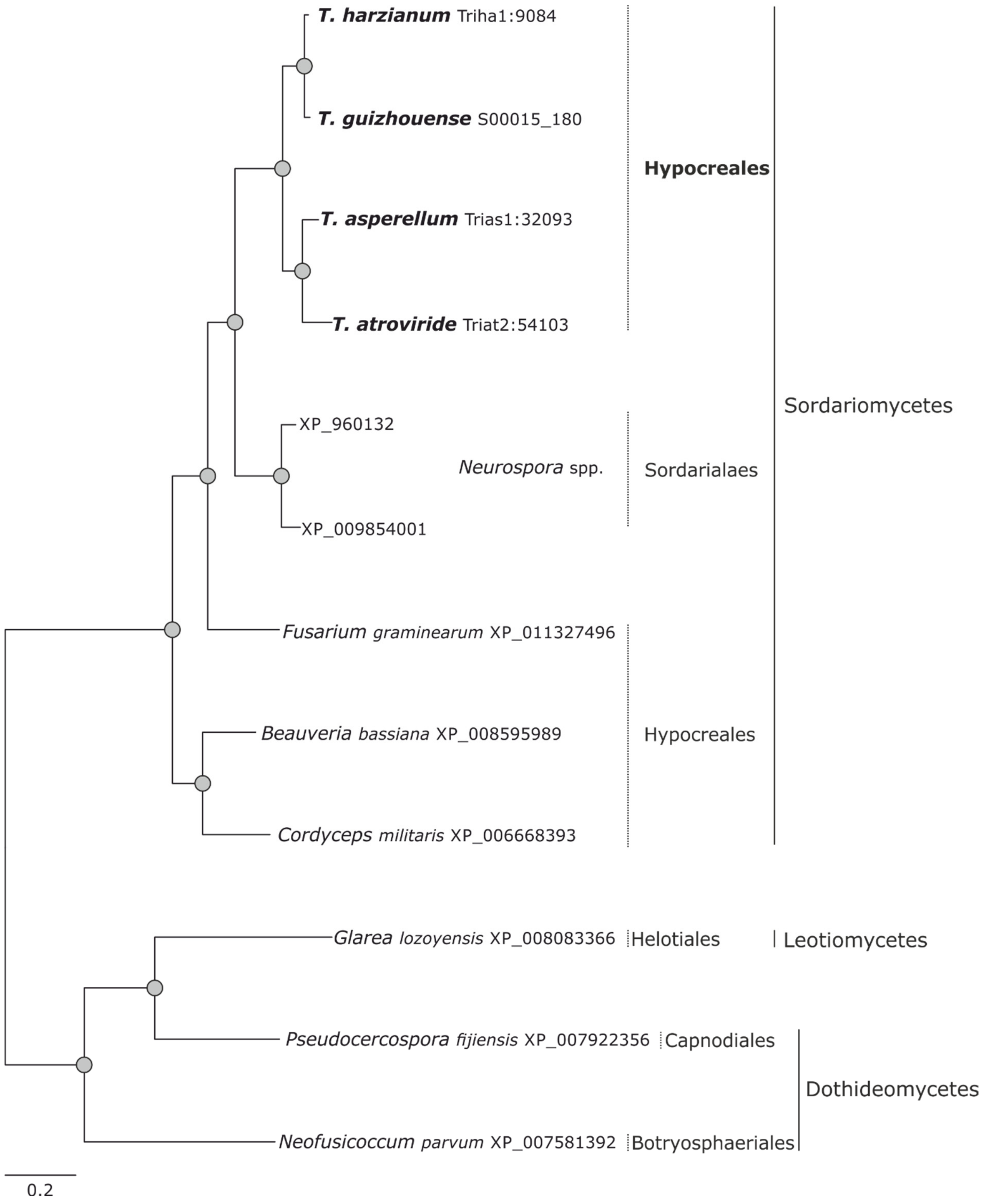

Phylogram based on Dayhoff amino acid substitution model using an alignment containing 10932 characters. Bayesian analysis was run for 1 million mcmc generations and a strict consensus tree was obtained by summarizing 7500 trees, after burning first 25% of obtained 10,000 trees. Mean tree length and variance are 3.748802e+000 and 8.399156e-003, respectively. Clades with Posterior probabilities less than 95% are marked with circular nodes. Dashed vertical bars and non-dashed vertical bars represents the taxonomic order and taxonomic class in the phylum Ascomycota respectively.

GH127 EC 3.2.1.185 β-L-arabinofuranosidase Clade A

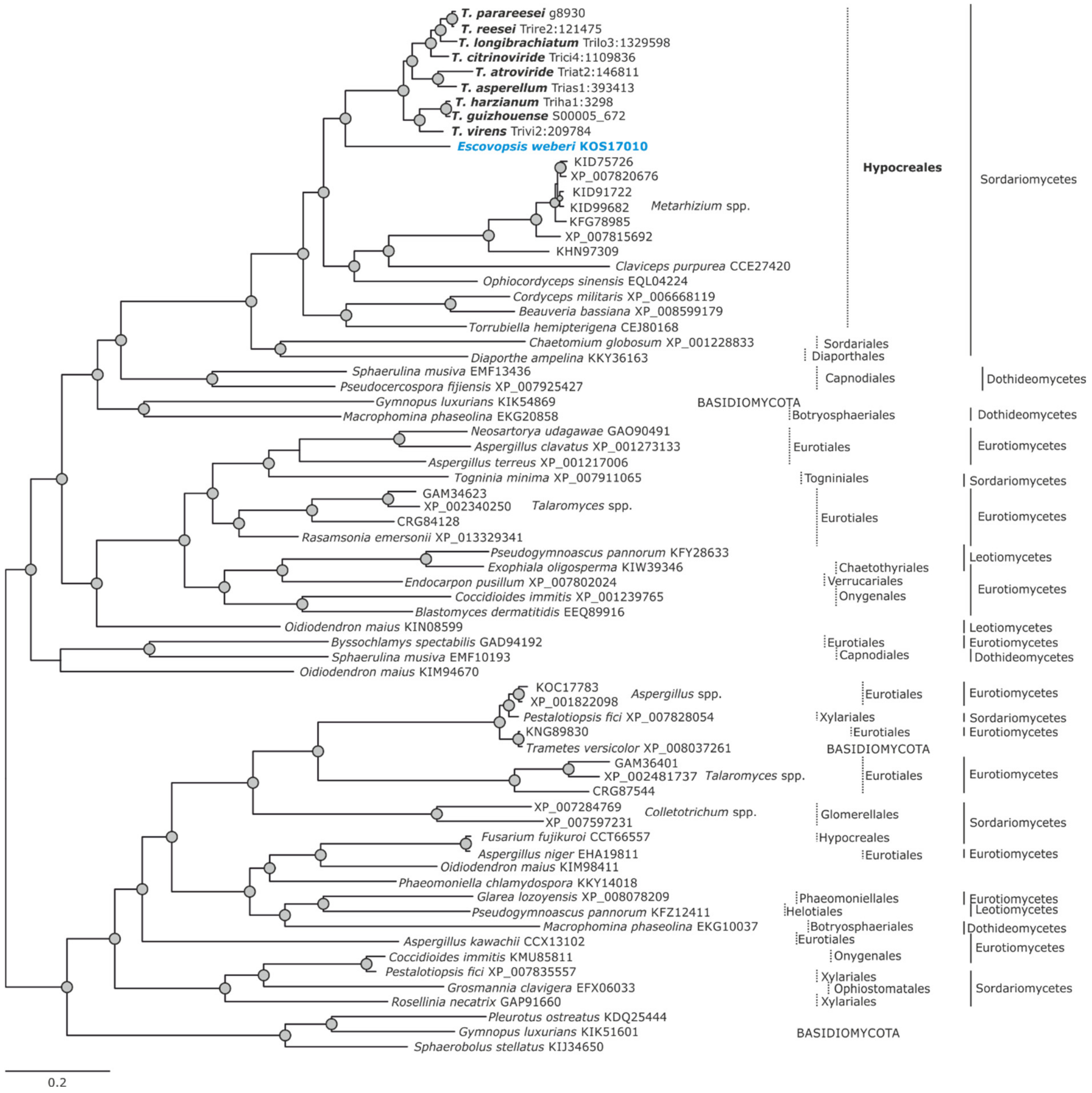

Phylogram based on Dayhoff amino acid substitution model using an alignment containing 56140 characters. Bayesian analysis was run for 1 million mcmc generations and a strict consensus tree was obtained by summarizing 7500 trees, after burning first 25% of obtained 10,000 trees. Mean tree length and variance are 1.939592e+001 and 3.952966e-002, respectively. Clades with Posterior probabilities less than 95% are marked with circular nodes. Dashed vertical bars and non-dashed vertical bars represents the taxonomic order and taxonomic class.

GH127 EC 3.2.1.185 β-L-arabinofuranosidase Clade B

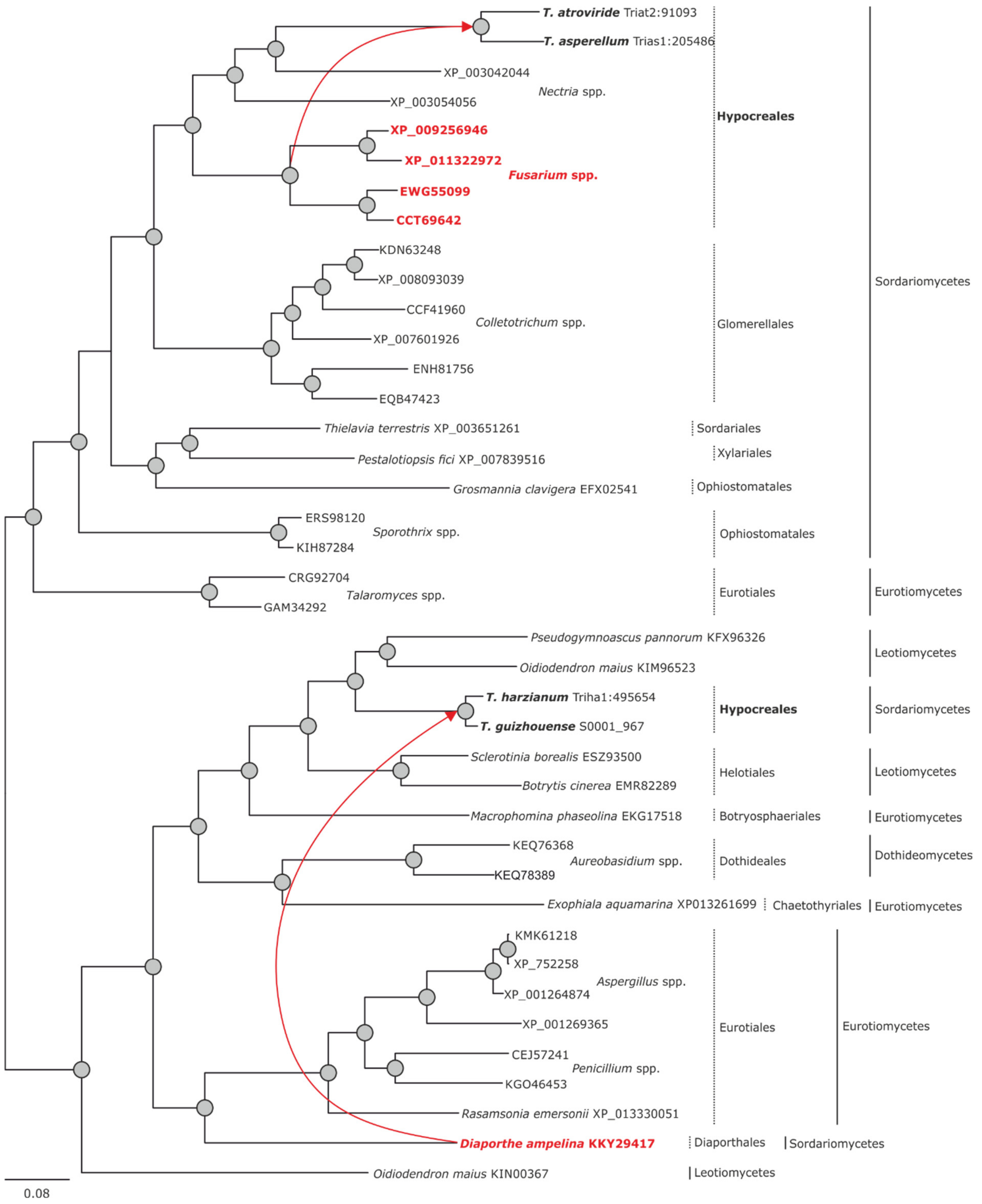

Phylogram based on Dayhoff amino acid substitution model using an alignment containing 27680 characters. Bayesian analysis was run for 1 million mcmc generations and a strict consensus tree was obtained by summarizing 7500 trees, after burning first 25% of obtained 10,000 trees. Clades with Posterior probabilities less than 95% are marked with circular nodes. Dashed vertical bars and non-dashed vertical bars represents the taxonomic order and taxonomic class. LGT inferred by Phylogeny and NOTUNG is shown with a red arrow to the node and the respective donor is marked in red.

AA9 (formerly "GH61") Copper-dependent lytic polysaccharide monooxygenase

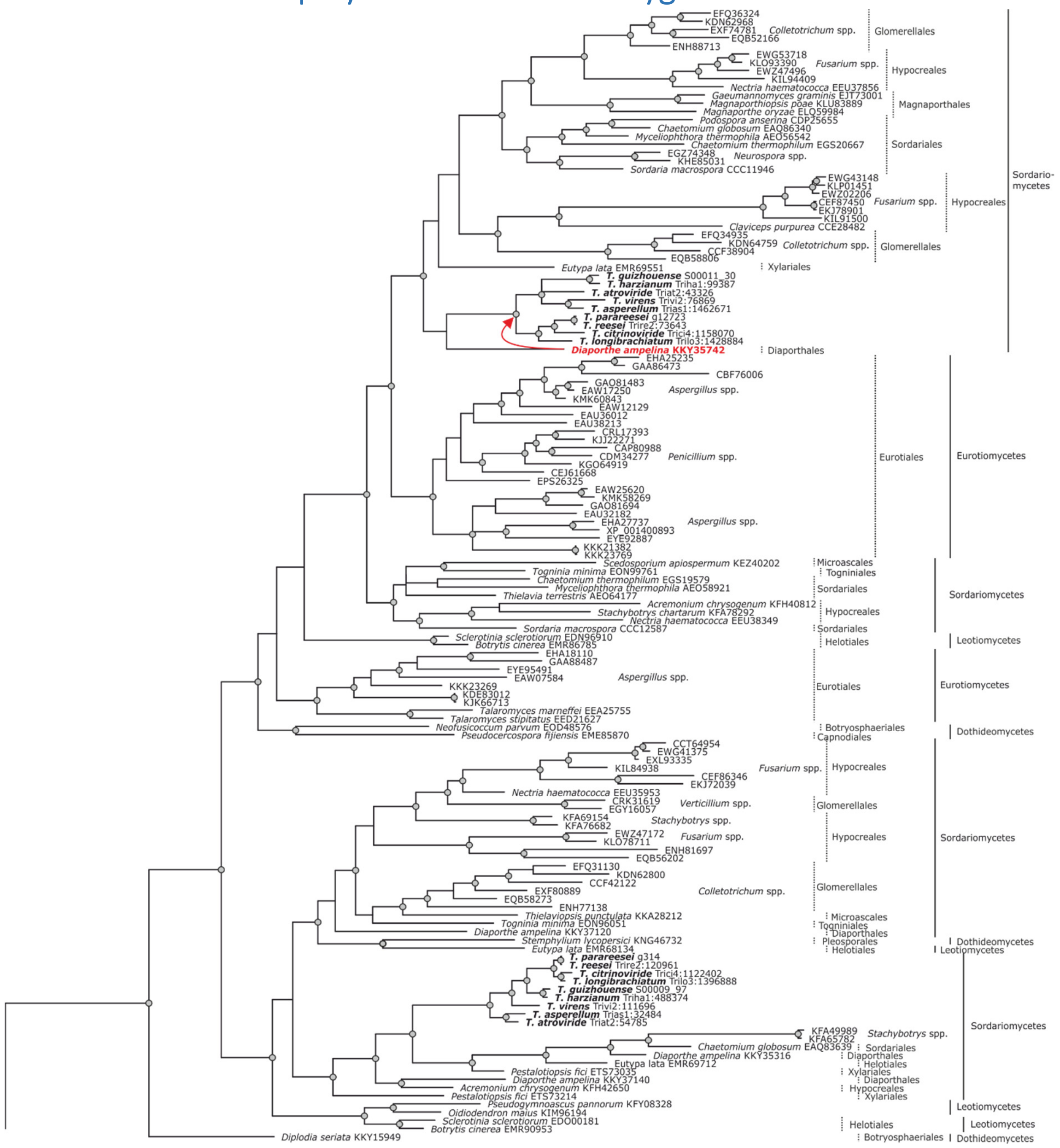

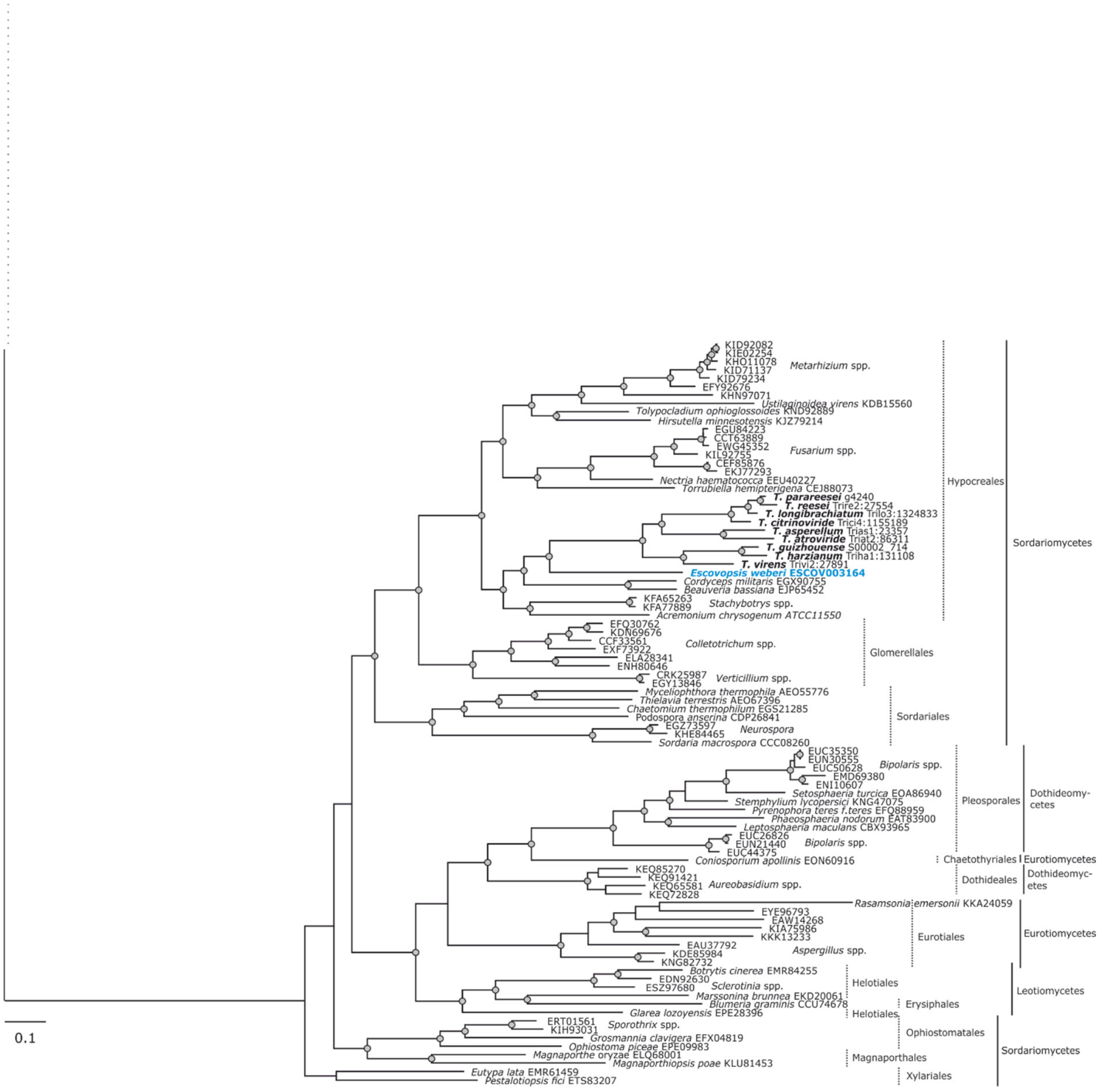

Phylogram based on Dayhoff amino acid substitution model using an alignment containing 129950 characters. Bayesian analysis was run for 1 million mcmc generations and a strict consensus tree was obtained by summarizing 7500 trees, after burning first 25% of obtained 10,000 trees. Mean tree length and variance are 5.586972E+01 and 2.1853650E-01, respectively. Clades with Posterior probabilities less than 95% are marked with circular nodes. Dashed vertical bars and non-dashed vertical bars represents the taxonomic order and taxonomic class.

Swollenin

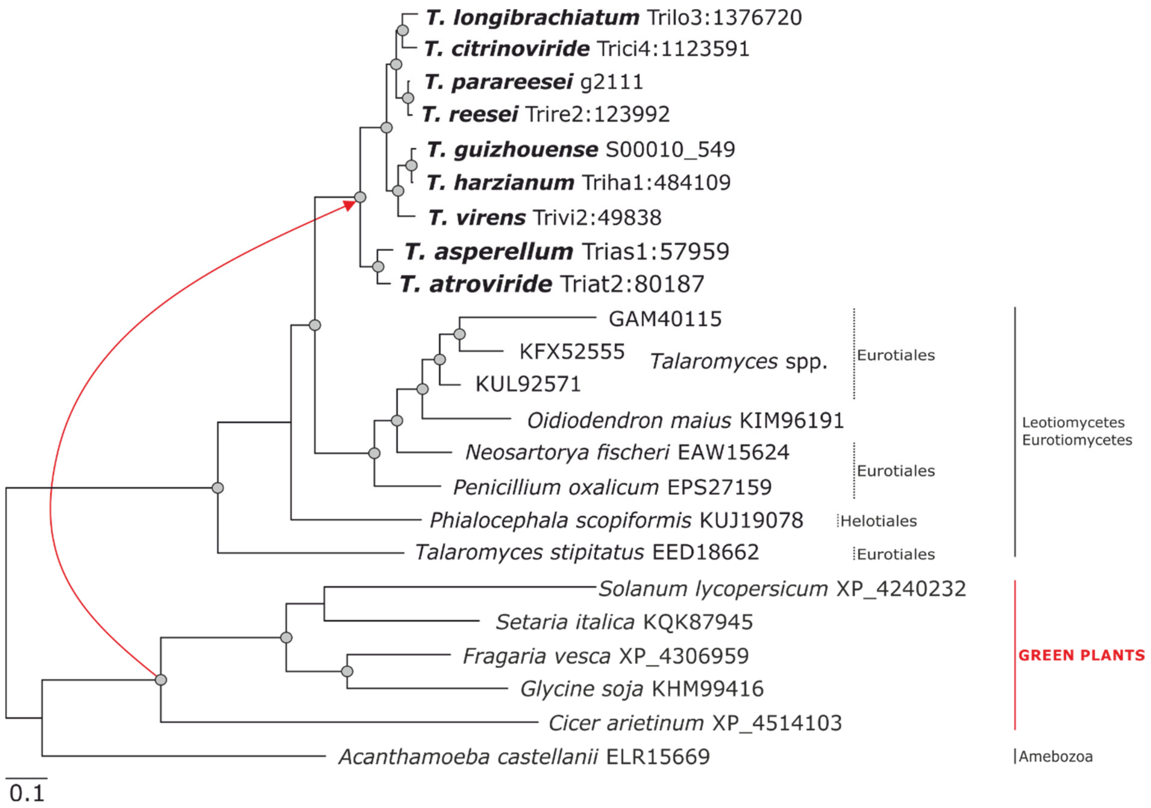

Phylogram based on Dayhoff amino acid substitution model using an alignment containing 12144 characters. Bayesian analysis was run for 1 million mcmc generations and a strict consensus tree was obtained by summarizing 7500 trees, after burning first 25% of obtained 10,000 trees. Mean tree length and variance are 7.653121E+00 and 4.9981080E-02, respectively. Clades with Posterior probabilities less than 95% are marked with circular nodes. Dashed vertical bars and non-dashed vertical bars represents the taxonomic order and taxonomic class. LGT inferred by Phylogeny and NOTUNG is shown with a red arrow to the node and the respective donor is marked in red.

pcwdCAZyme Regulatory Proteins

XYR1

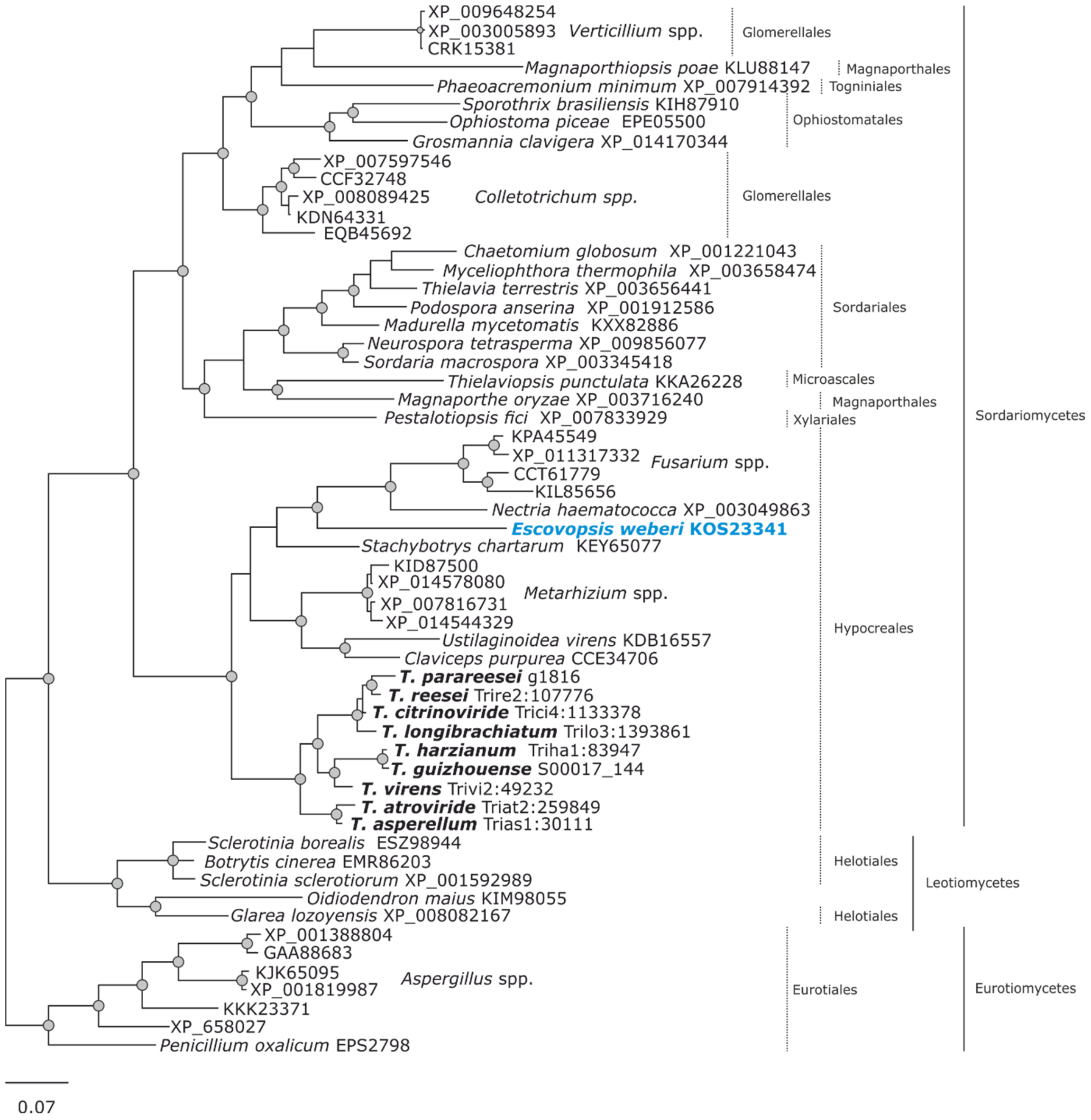

Phylogram based on Dayhoff amino acid substitution model using an alignment containing 18753 characters. Bayesian analysis was run for 1 million mcmc generations and a strict consensus tree was obtained by summarizing 7500 trees, after burning first 25% of obtained 10,000 trees. Mean tree length and variance are 5.781707E+00 and 2.0201740E-02, respectively. Clades with Posterior probabilities less than 95% are marked with circular nodes. Dashed vertical bars and non-dashed vertical bars represents the taxonomic order and taxonomic class.

ACE2

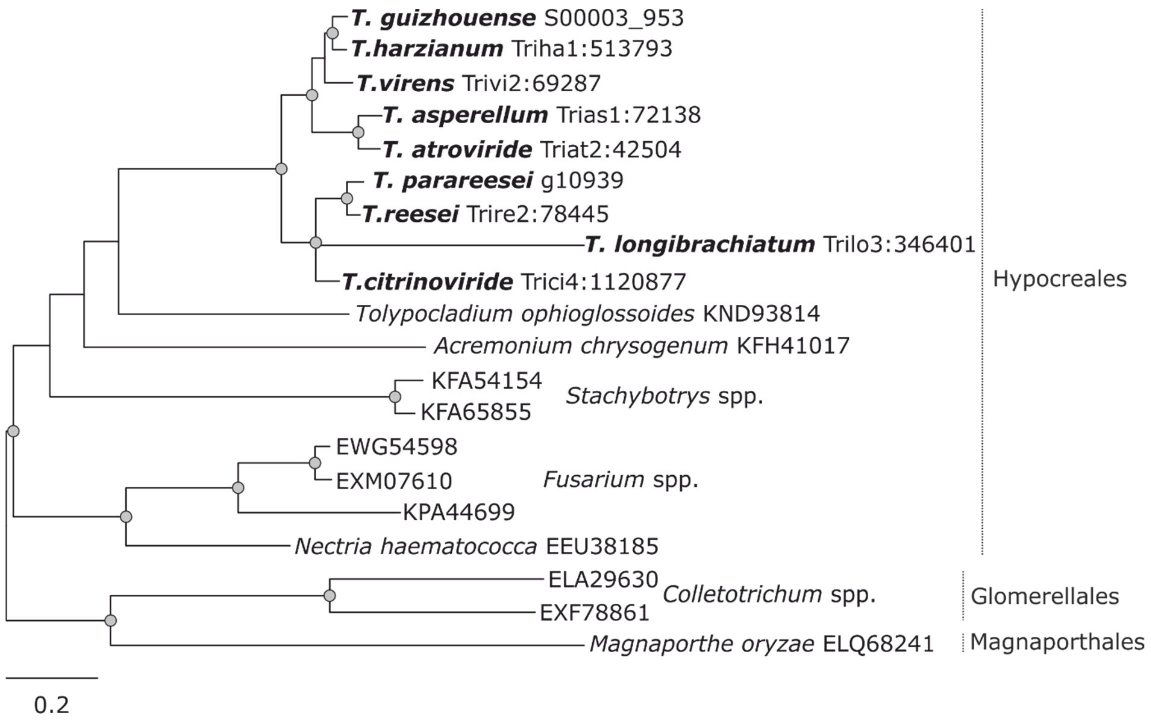

***All species fall under taxonomic class Sordariomycetes**

Phylogram based on Dayhoff amino acid substitution model using an alignment containing 5910 characters. Bayesian analysis was run for 1 million mcmc generations and a strict consensus tree was obtained by summarizing 7500 trees, after burning first 25% of obtained 10,000 trees. Mean tree length and variance are 8.181301E+00 and 5.4077250E-02, respectively. Posterior probabilities more than 95% are marked with circular nodes. Dashed vertical bars represents the taxonomic order.

ACE3

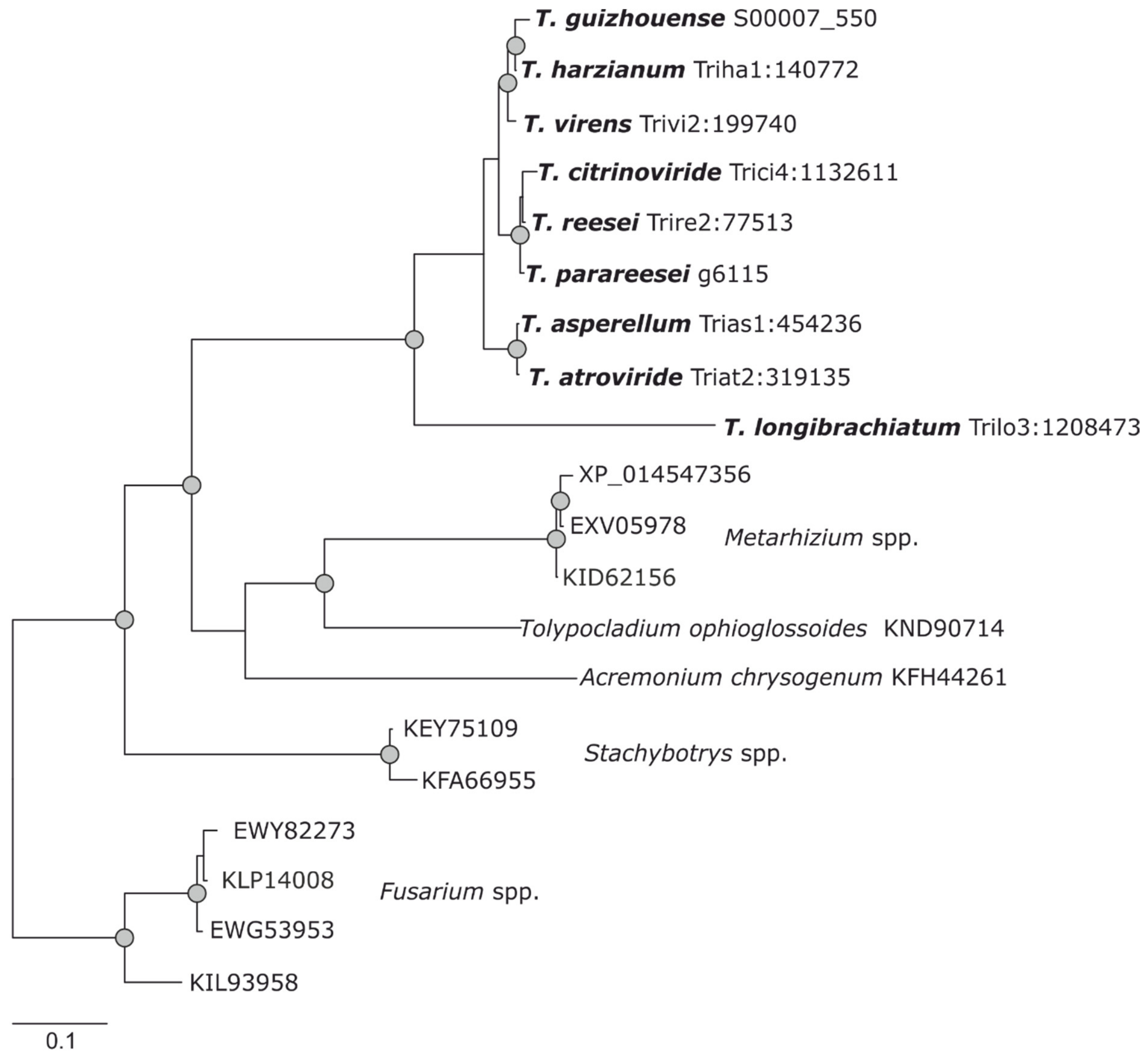

***All species fall under taxonomic class Sordariomycetes and taxonomic order Hypocreales**

Phylogram based on Dayhoff amino acid substitution model using an alignment containing 12155 characters. Bayesian analysis was run for 1 million mcmc generations and a strict consensus tree was obtained by summarizing 7500 trees, after burning first 25% of obtained 10,000 trees. Mean tree length and variance are 2.543828E+00 and 9.7324330E-03, respectively. Posterior probabilities more than 95% are marked with circular nodes.
