# Supplementary material for: Massive lateral transfer of genes encoding plant cell wall-degrading enzymes to the mycoparasitic fungus Trichoderma from its plant-associated hosts
Source: PLoS Genet. 2018 Apr 9;14(4):e1007322. doi: 10.1371/journal.pgen.1007322 (PMC5908196; doi:10.1371/journal.pgen.1007322)
Supplement: S3 Fig — A. Allomycoparasitism of Trichoderma spp. and E. weberi on Lentinula edodes. B: Allomycoparasitism of Trichoderma and E. weberi on Leucoagaricus gongylophorus. The dashed lines indicate growth of the host fungus as deduced from back sides of the plates. C: Set up for the microscopic investigation of Trichoderma (right) parasitism on Pestalotiopsis fici (left). 2 x 2 cm agar plugs were located between a sterile microscopy glass slide and a 5 x 2.5 cm sterile glass cover slip and aseptically inoculated with spores of two partner fungi, respectively, using a microbiological needle. Inoculated cultures were maintained at 28°C in wet chamber until hyphal contact. Microscopic investigation was done for hyphae on the cover slip surface. D. Antagonism of selected Trichoderma species on Penicillium spp. Dashed line indicates growth of the opponent fungus. (PDF) [file pgen.1007322.s011.pdf]

S3 Figure: Mycoparasitism of *Trichoderma*.

A. Allomycoparasitism of *Trichoderma* spp. and *E. weberi* on *Lentinula edodes.*

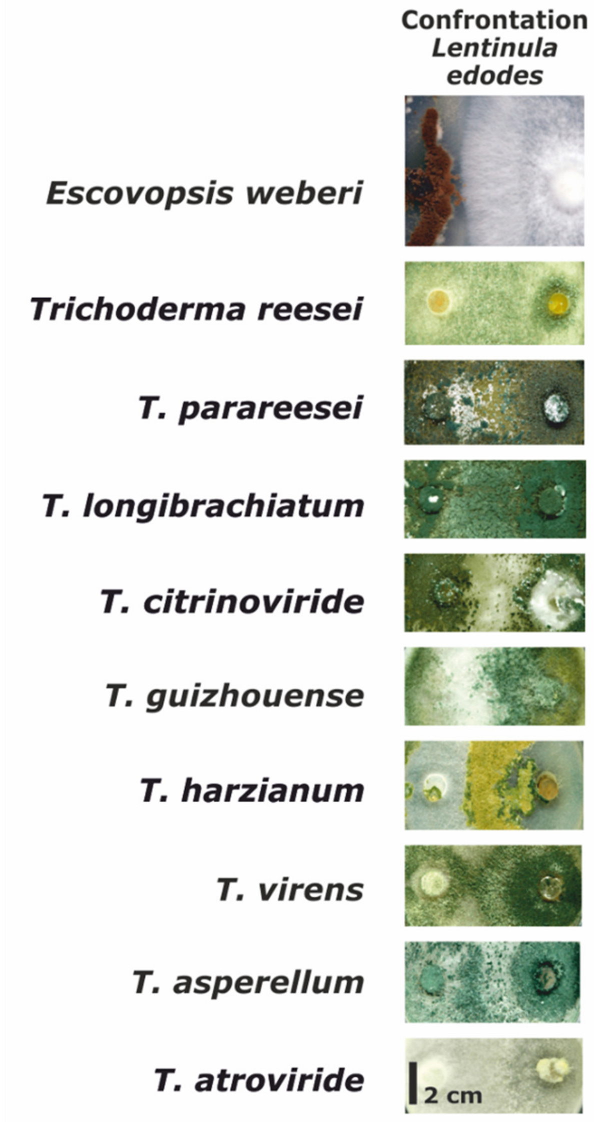

B. Allomycoparasitism of *Trichoderma* and *E. weberi* on *Leucoagaricus gongylophorus*. The dashed lines indicate growth of the host fungus as deduced from back sides of the plates.

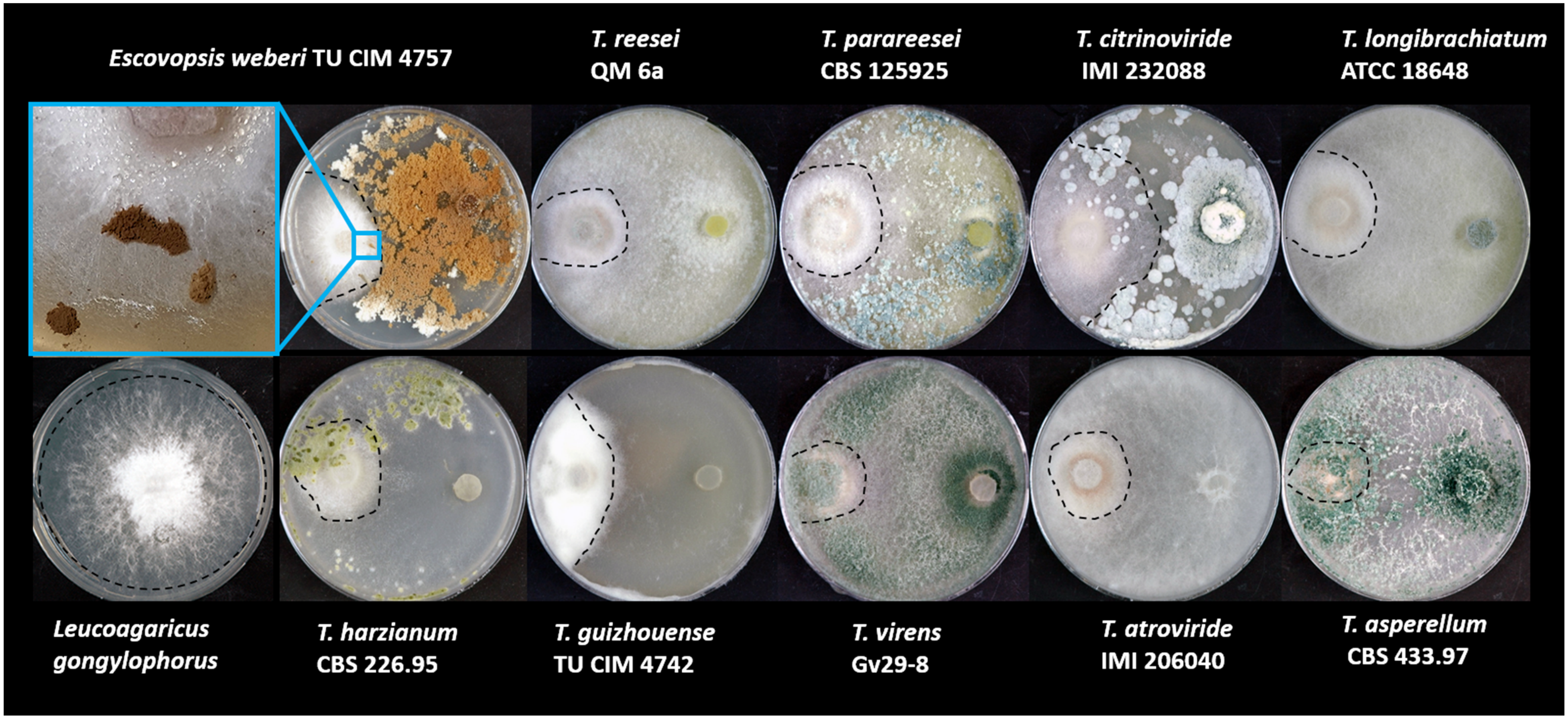

C. Set up for the microscopic investigation of *Trichoderma* (right) parasitism on *Pestalotiopsis fici* (left). 2 cm2 agar plugs were located between a sterile microscopy glass slide and a 5 x 2.5 cm sterile glass cover slip and aseptically inoculated with spores of two partner fungi, respectively, using a microbiological needle. Inoculated cultures were maintained at 28˚C in wet chamber until hyphal contact. Microscopic investigation was done for hyphae on the cover slip surface.

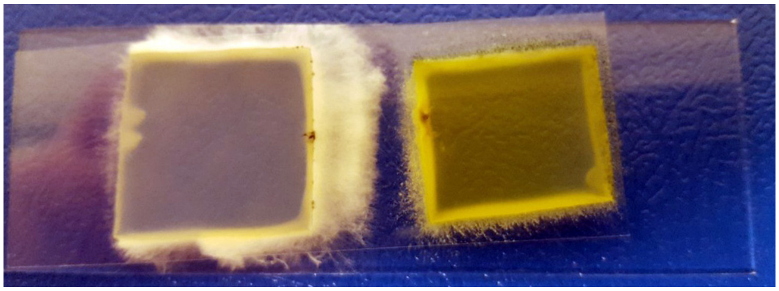

D. Antagonism of selected *Trichoderma* species on *Penicillium* spp. Dashed line indicates growth of the host fungus.

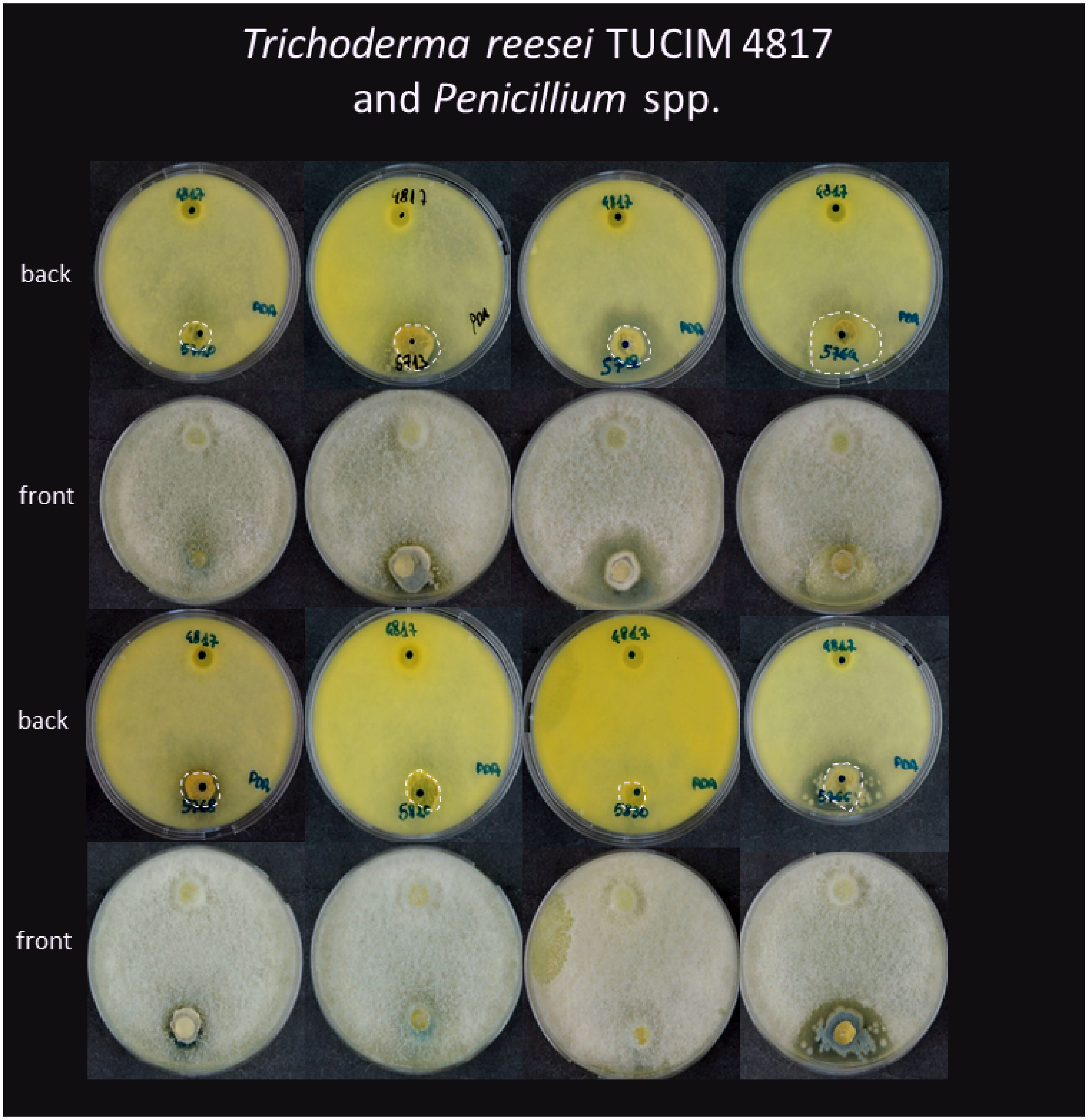

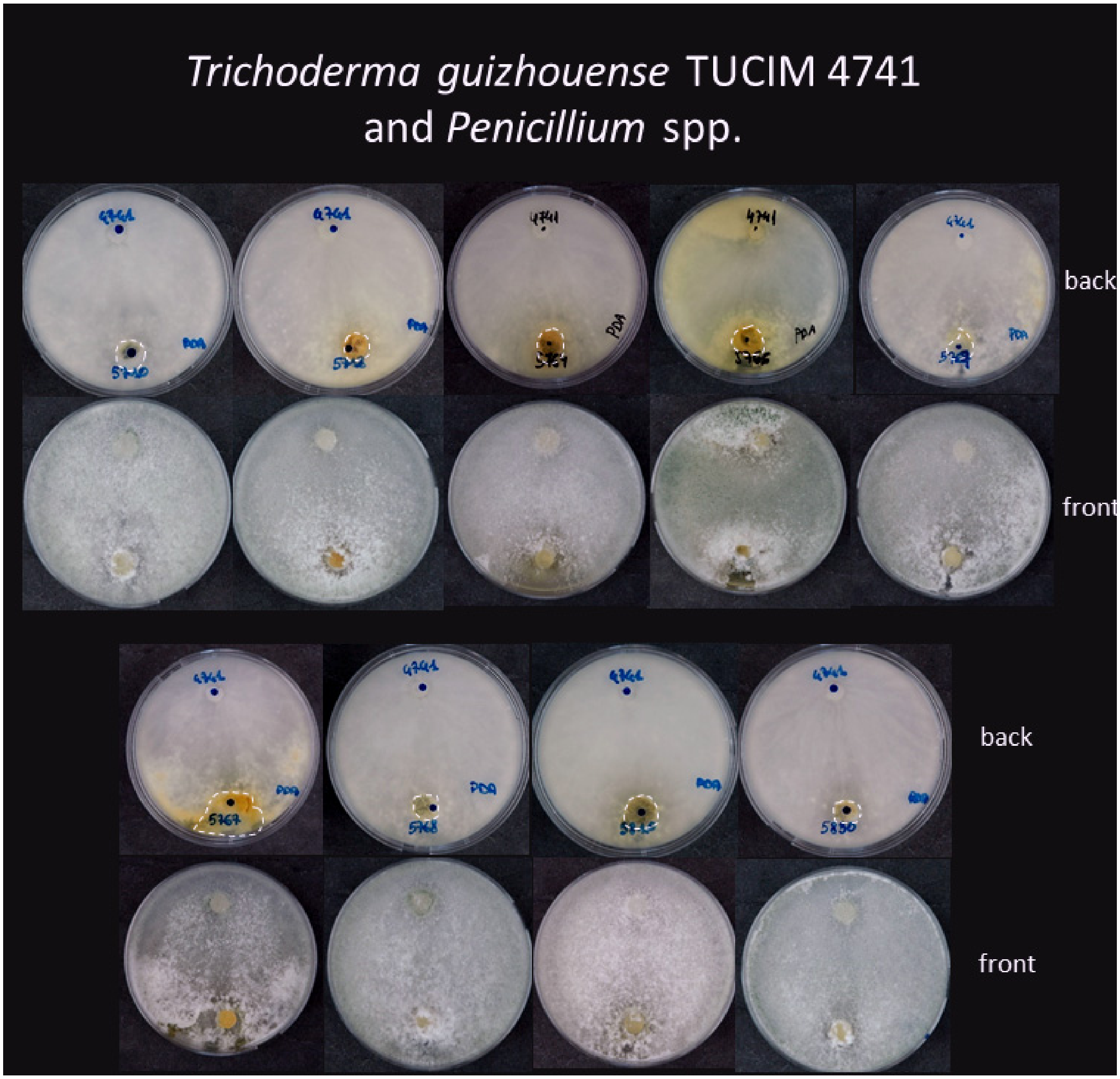
Trichoderma guizhouense TUCIM 4741
and *Penicillium* spp.
back
front
back
front
